# Increasing both the public health potential of basic research and the scientist satisfaction. An international survey of bio-scientists

**DOI:** 10.12688/f1000research.7683.2

**Published:** 2016-06-01

**Authors:** Carmen Sorrentino, Andrea Boggio, Stefano Confalonieri, David Hemenway, Giorgio Scita, Andrea Ballabeni

**Affiliations:** 1 IFOM - The FIRC Institute of Molecular Oncology, Milan, Italy; 2 Department of History and Social Science, Bryant University, Smithfield, RI, USA; 3 Department of Health Policy and Management, Harvard T.H. Chan School of Public Health, Boston, MA, USA; 4 Department of Oncology and Hemato-Oncology-DIPO, School of Medicine, University of Milan, Milan, Italy

**Keywords:** Basic research, Public health, Scientist satisfaction, Policy, Nudges, Grant, Basic bibliography

## Abstract

Basic scientific research generates knowledge that has intrinsic value which is independent of future applications. Basic research may also lead to practical benefits, such as a new drug or diagnostic method. Building on our previous study of basic biomedical and biological researchers at Harvard, we present findings from a new survey of similar scientists from three countries. The goal of this study was to design policies to enhance both the public health potential and the work satisfaction and test scientists’ attitudes towards these factors. The present survey asked about the scientists’ motivations, goals and perspectives along with their attitudes concerning  policies designed to increase both the practical (i.e. public health) benefits of basic research as well as their own personal satisfaction. Close to 900 basic investigators responded to the survey; results corroborate the main findings from the previous survey of Harvard scientists. In addition, we find that most bioscientists disfavor present policies that require a discussion of the public health potential of their proposals in grants but generally favor softer policies aimed at increasing the quality of work and the potential practical benefits of basic research. In particular, bioscientists are generally supportive of those policies entailing the organization of more meetings between scientists and the general public, the organization of more academic discussion about the role of scientists in the society, and the implementation of a “basic bibliography” for each new approved drug.

## Introduction

Basic research has been crucial for the improvement of the human condition, including both research inspired solely by scientific curiosity and research driven by a vision of future applications. While basic knowledge is inherently valuable, all basic knowledge does not have the same potential for practical benefits. Of course, it is often difficult *a priori* as well as *a posteriori* to determine which knowledge will have, or has had, a greater impact on society *,* and some knowledge may never have any utility, neither direct nor indirect, in producing any practical outcome ^1– 17^. However, although we cannot know the future practical benefits of basic research, it is generally possible to make rough estimates of the potential.

We created a survey to assess the attitudes and beliefs of basic scientific researchers concerning policies that might incentivize bioscientists to engage in basic research with a higher likelihood of creating public health benefits, without compromising the “basic” nature of their research. We focused on policies based on soft incentives (what behavioral economists call “nudges”) ^18^ because we believed that, if properly tailored to basic scientists motivations and goals ^19^, soft policies could effectively stir (some) basic scientists towards research with a greater potential of creating larger public health benefits without overly constraining their research or decreasing their work satisfaction.

To determine which “nudges” might be effective, it is key to have a good grasp of what motivates basic scientists and of the intellectual framework in which they operate. To explore these questions, we previously conducted a study (Study 1) at a single institution (Harvard University and affiliated institutions in the Boston (MA-USA) area) to collect preliminary data and refine our hypotheses ^20^. We found that the vast majority of the biological/biomedical scientists at Harvard University believe that, although is it often difficult to assess the potential future health benefits to society from basic research proposals, or actual research findings, some degree of estimation is possible. These bioscientists also supported the idea that softer policies are preferable to stricter ones for increasing the societal benefits of research.

Study 1 showed that increasing people’s health and personal prestige are some of the strongest motivations for basic scientists. Moreover, it showed that basic scientists strongly support the idea of non-mandatory policies based on soft incentives to increase public health potential and work satisfaction. Based on these and other findings of Study 1, we designed this second study (Study 2) to obtain a larger sample of basic bioscientists, from multiple institutions and different countries. We used a slightly modified version of the Study 1 questionnaire. In particular, we added a few questions asking respondents to evaluate current policies used to evaluate/increase the public health potential, as well as six new soft policies that we developed after analyzing the results of Study 1. In the current paper, we present the results of Study 2.

Just under 900 basic bioscientists responded to the survey, completing an online questionnaire (see Methods section). Study 2 confirms the main findings of Study 1 with regard to motivations of basic scientists and how they conceptualize basic research. In particular, the vast majority of respondents reported being driven not only by curiosity or the desire of knowledge advancement but also by the aspiration of having an impact on people’s health. Respondents also think that basic scientists can ponder future practical benefits of their research without losing their “basic status” and that it is possible to roughly estimate the practical potential of basic research proposals. Finally, participants, especially principal investigators (PIs), disfavor current policies requiring the discussion of the potential societal impact in research proposals but favor the new policies we propose.

## Methods

The survey was an anonymous online questionnaire (see Questionnaire in the Data availability section) that was sent by email to scientists working at institutions where basic research in the biological/biomedical area is routinely conducted. The research instrument for Study 2 was a modified and expanded version of the questionnaire used in Study 1. In particular, we used a subset of the questions used in Study 1 and added questions on some current policies and on six policies that we propose.

Over seven thousand (7,786) scientists were contacted from over thirty institutions in four different geographical locations [Los Angeles-San Diego (CA-USA), London-Cambridge (UK), Milan (Italy), and New York City (NY-USA)]. Invited scientists from the Los Angeles-San Diego area were affiliated with Calibr (California Institute for Biomedical Research), Caltech (California Institute of Technology), Cedars-Sinai, Salk Institute, Sanford-Burnham Medical Research Institute, Scripps Research Institute, UCI (University of California Irvine), UCLA (University of California Los Angeles), UCR (University of California Riverside), UCSD (University of California San Diego) or USC (University of Southern California). Invited scientists from the London-Cambridge area were affiliated with Francis Crick Institute, ICL (Imperial College London), ICR (Institute of Cancer Research), King’s College, UCL (University College London), University of London Birkbeck, University of London Queen Mary, University of London St George’s or the University of Cambridge. Invited scientists from the Milan area were affiliated with Humanitas Research Hospital, IEO (European Institute of Oncology), IFOM (FIRC Institute of Molecular Oncology), INGM (National Institute of Molecular Genetics), Istituto Nazionale dei Tumori (National Institute of Tumors), Mario Negri Institute, San Raffaele Hospital, University of Milan or University of Milan-Bicocca. Invited scientists from the New York City area were affiliated with Albert Einstein College of Medicine, Columbia University, CSHL (Cold Spring Harbor Laboratories), CUNY ASRC (City University of New York Advanced Science Research Center), CUNY Queens (City University of New York Queens college), Memorial Sloan Kettering Cancer Center, Mount Sinai Hospital, NYU (New York University) or Rockefeller University.

Email addresses were taken from the publicly accessible websites of the institutes. Invitations were sent to individual emails. The invitations contained a standard text of invitation and brief explanation of the study; emails of invitations differed one from another only with regard to the name of the invited scientist in the salutation (“Dear xxx”).

Participation in the study was voluntary and entailed answering an online survey powered by Qualtrics software ( www.qualtrics.com) through the Harvard T.H. Chan School of Public Health. Respondents could skip any questions they wanted to. They were asked to confirm their status as basic researchers (see Results section). We invited all types of bioscientists, with regard to their position/role, except that we tried not to invite undergraduate students and non-PhD technicians.

We tried to keep the proportion of invitations sent to PIs at over 50%. We did not invite every single scientist at every institute but for each institute decided *a priori* the number of scientists and then used the alphabetical order. Respondents had the option of indicating their geographical location but not their institution. The differences in gender invitations were not deliberate but a reflection of the actual proportions of females and males. Graphs describing the statistics of the invitations are shown in Figures S1–S6.

The invitations were sent from August 24, 2015 to October 10, 2015. Force completion of the survey was set at 72 hours. The study was reviewed and approved by the Harvard T.H. Chan School of Public Health IRB (IRB15-2787) and by the FIRC Institute of Molecular Oncology Ethics Committee.

## Results

Questions and responses of the survey “QUESTIONNAIRE.pdf” contains the questions of the questionnaire. “QUESTIONNAIRE DATA.csv” contains the responses of the questionnaire. “FINAL REPORT.pdf” contains the summary of the responses of the questionnaire. Click here for additional data file. Copyright: © 2016 Sorrentino C et al. 2016 Data associated with the article are available under the terms of the Creative Commons Zero "No rights reserved" data waiver (CC0 1.0 Public domain dedication).

## Overview of the sample

Close to 900 (885) scientists responded to the survey. The overall response rate was 11.4%. The response rates for females and males were 12.8% and 10.0%, respectively (the response rates for females were higher in all geographical locations). The response rate for PIs was 10.5% (12.4% for female PIs and 9.6% for male PIs). Detailed response rates are shown in Figures S7–S11. There were 464 respondents who reported PI status, 219 post-docs, 94 PhD students and 109 other/unspecified roles.

More males (500) than females (359) participated in the study. The average age was 43.4 years old. 202 worked in the Los Angeles-San Diego area (CA-USA), 180 in the London-Cambridge area (UK), 223 in the Milan area (Italy), and 238 in the New York City area (NY-USA). From the question “Approximately, what percentage of your research do you consider to be basic?” the average level of involvement in basic research was 78.0% (2.7% of the respondents skipped this specific question). Only 3 respondents (0.3%) declared they were not involved in basic research at all (i.e. 0% of basic research). Questions were skipped in the range of 2.3% (question with the lowest skipping rate) to 13.7% (question with the highest skipping rate). The number of responses according to role, gender, geographical location and level of involvement in basic research are shown in Figures S12–S18. All responses are presented in the Final report in the Data availability section.

## The motivations of the basic scientists

**Figure 1.**
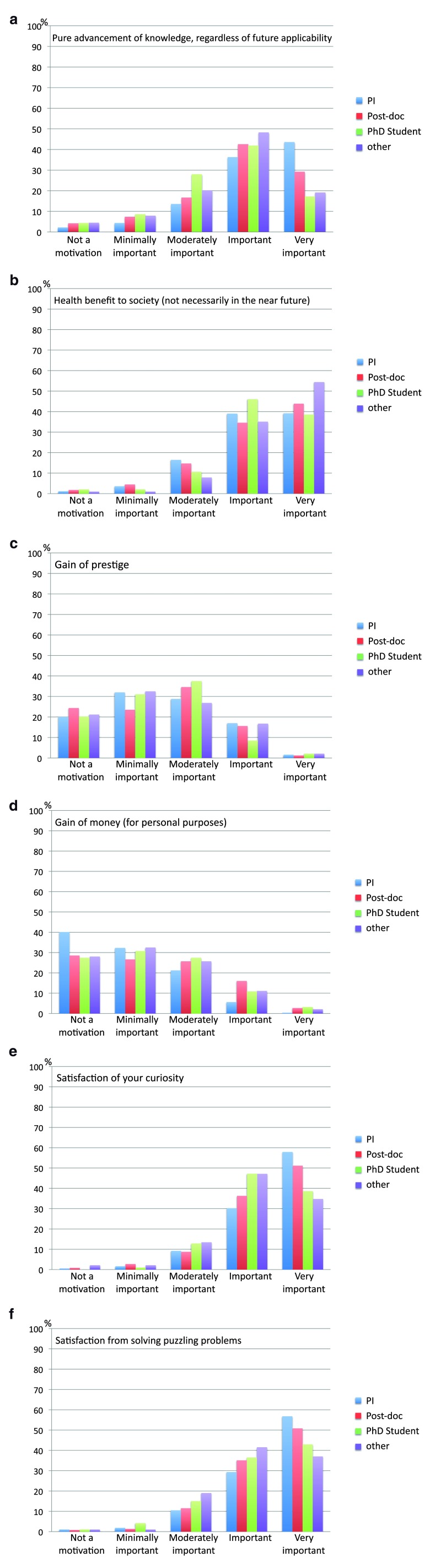
Your personal motivations as a scientist are from: ( **a**) Pure advancement of knowledge, regardless of future applicability, ( **b**) Health benefit to society (not necessarily in the near future), ( **c**) Gain of prestige, ( **d**) Gain of money (for personal purposes), ( **e**) Satisfaction of your curiosity, ( **f**) Satisfaction from solving puzzling problems.

We asked participants to rank their motivations for research by level of importance. “Health benefit to society”, “satisfaction of curiosity”, and “satisfaction from solving puzzling problems” were the most important motivations while “gain of prestige” and “gain of money” were less important motivations. “Pure advancement of knowledge” was a strong motivator, especially among principal investigators. These results confirmed the findings of Study 1 ( Figures 1a–f) (detailed data for all figures in this paper are shown in the associated tables in Supplementary material). For the motivations we also performed analyses based on the roles of the scientists. We observed that some differences were statistically significant. In particular, PIs are more motivated from “Pure advancement of knowledge” (p = 0.0042) and less motivated from “Gain of money” (p<0.0001) in comparison to post-docs. Moreover, PIs are more motivated from “Pure advancement of knowledge” (p<0.0001) and “Satisfaction of curiosity” (p = 0.0068) and less motivaed from “Gain of money” (p = 0.0142) in comparison to students.

**Figure 2.**
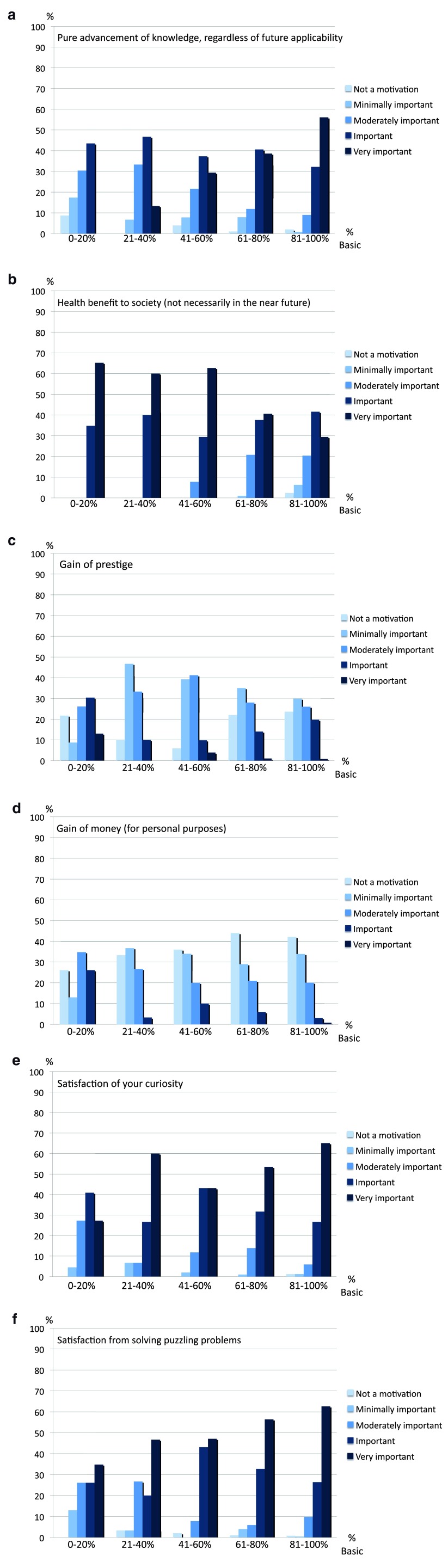
Your personal motivations as a scientist are from: ( **a**) Pure advancement of knowledge, regardless of future applicability, ( **b**) Health benefit to society (not necessarily in the near future), ( **c**) Gain of prestige, ( **d**) Gain of money (for personal purposes), ( **e**) Satisfaction of your curiosity, ( **f**) Satisfaction from solving puzzling problems. (Principal Investigators ordered by percentage of basic research).

For PIs, their level of involvement in basic research was positively correlated with the motivations “pure advancement of knowledge,” “satisfaction of curiosity,” and “satisfaction from solving puzzling problems” and negatively correlated with the motivations “health benefit to society,” “gain of prestige,” and “gain of money” ( Figures 2a–f).

## The concept of basic research and its practical benefit potential

**Figure 3.**
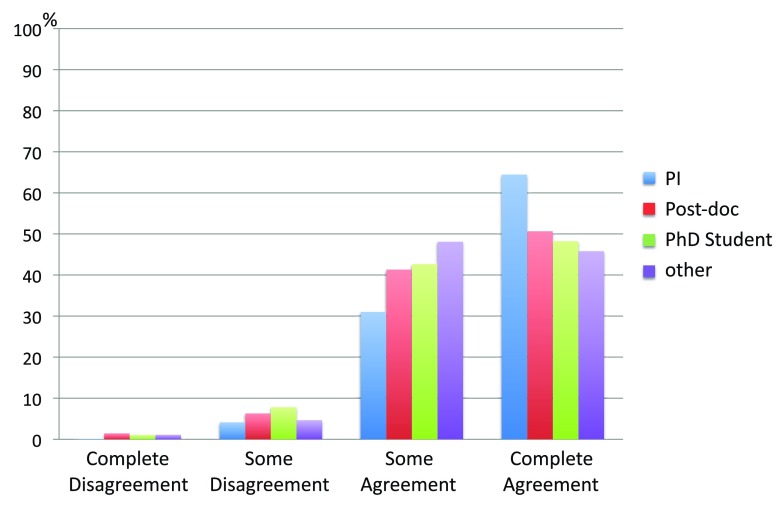
Basic scientists can ponder about the future indirect practical benefits of their research without losing their “basic status”.

**Figure 4.**
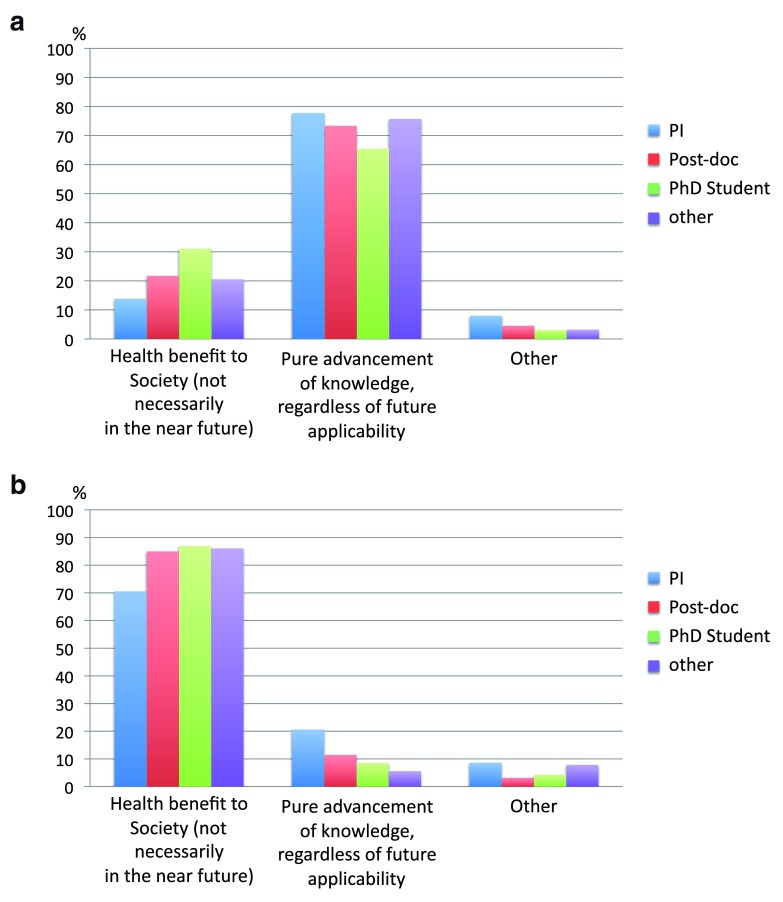
What should the most important goal of publicly funded basic: ( **a**) BIOLOGICAL (not biomedical) research be? ( **b**) BIOMEDICAL research be?

**Figure 5.**
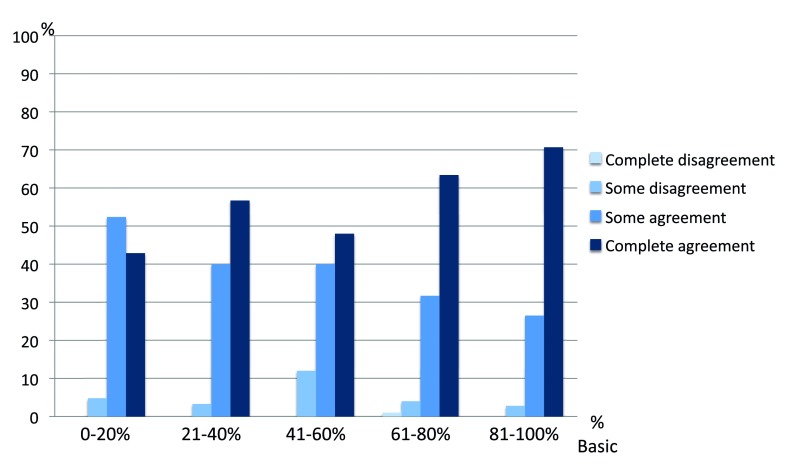
Basic scientists can ponder about the future indirect practical benefits of their research without losing their “basic status”. Principal investigators ordered by percentage of basic research.

**Figure 6.**
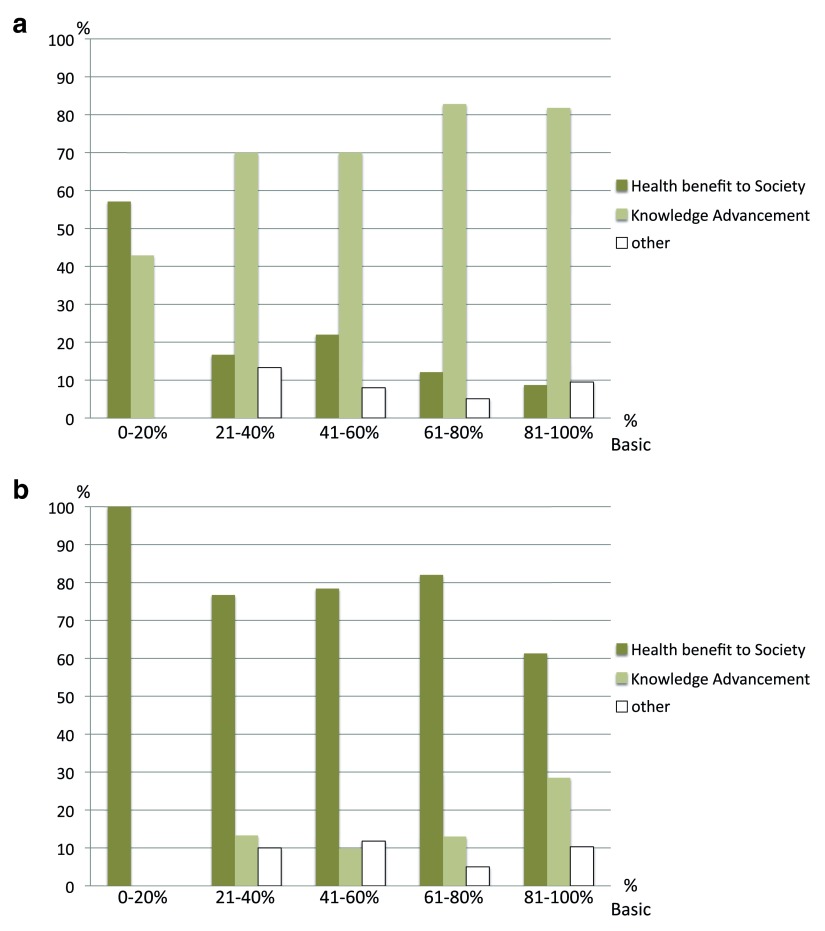
What should the most important goal of publicly funded basic: ( **a**) BIOLOGICAL (not biomedical) research be? ( **b**) BIOMEDICAL research be? (Principal investigators ordered by percentage of basic research).

The vast majority of the surveyed scientists were in some or complete agreement with the statement: “basic scientists can ponder about the future indirect practical benefits of their research without losing their ‘basic status’” ( Figure 3). The majority indicated that the most important goal of publicly funded basic *biological* research should be “pure advancement of knowledge, regardless of future applicability” ( Figure 4a) and of funded basic *biomedical* research should be the “health benefit to society (not necessarily in the near future)” ( Figure 4b). PIs with more involvement in basic research were more likely to agree with the statement “basic scientists can ponder about the future indirect practical benefits of their research without losing their ‘basic status’” ( Figure 5) and that “pure knowledge advancement” is the main goal of basic research ( Figures 6a–b).

## The policy of discussing health benefits in research proposals

**Figure 7.**
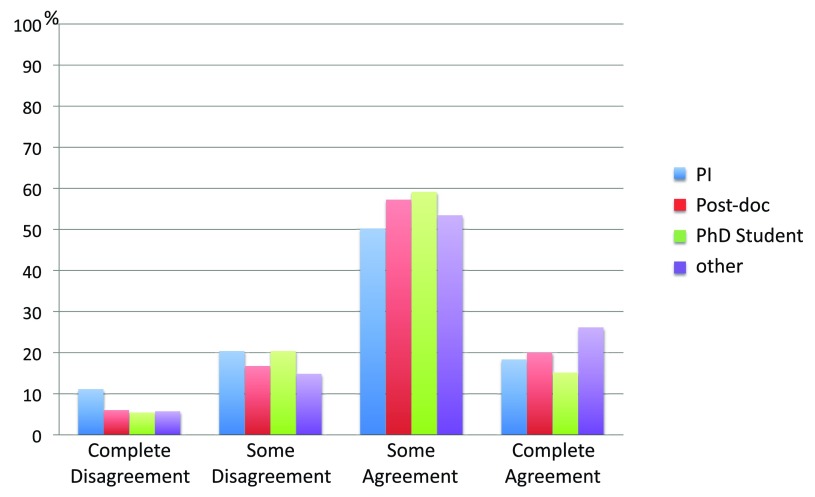
Although it is difficult to assess the potential future health benefits to society from basic biological/biomedical research as described in written proposals, some degree of estimation is always possible.

**Figure 8.**
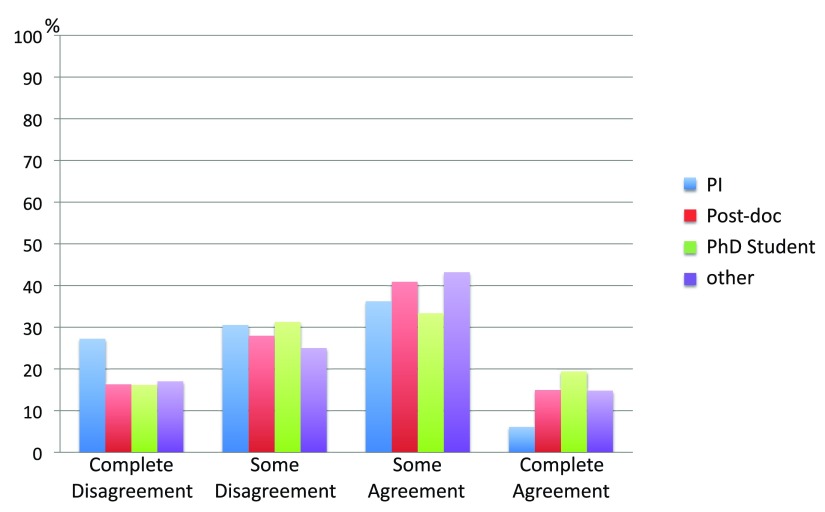
Written proposals about basic biological/biomedical research generally contain a section discussing potential future health benefits. These sections increase the likelihood that a project benefits future public health.

Over 70% of respondents expressed at least some agreement with the statement: “although it is difficult to assess the potential future health benefits to society from basic biological/biomedical research as described in written proposals, some degree of estimation is always possible” ( Figure 7). However, the level of agreement was significantly lower with the statement: “written proposals about basic biological/biomedical research generally contain a section discussing potential future health benefits. These sections increase the likelihood that a project benefits future public health.” The difference was especially important for PIs, who expressed the highest degree of disagreement with the second statement ( Figure 8).

**Figure 9.**
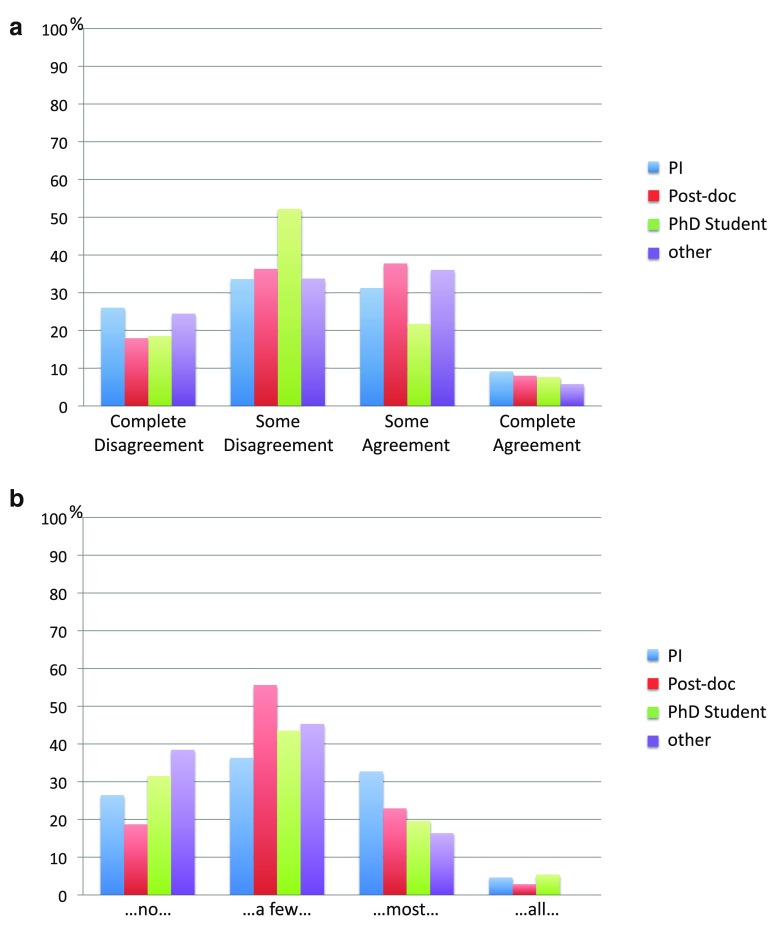
( **a**) Writing the sections discussing potential future health benefits takes too much time. ( **b**) The sections discussing potential future health benefits should be eliminated for .............. grants.

Almost half of respondents agreed with the statement that “writing the sections discussing potential future health benefits takes too much time” ( Figure 9a). For the statement “the sections discussing potential future health benefits should be eliminated for [no/a few/most/all] grants”, over 70% of respondents declared that these sections should be eliminated at least for some grants, including a significant portion (especially of PIs) that indicated that these sections should be eliminated for “most” or “all” grants ( Figure 9b).

**Figure 10.**
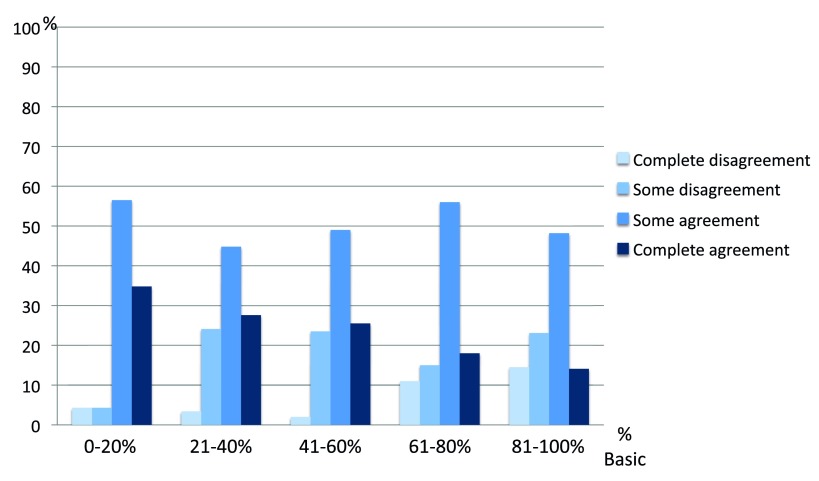
Although it is difficult to assess the potential future health benefits to society from basic biological/biomedical research as described in written proposals, some degree of estimation is always possible. Principal Investigators ordered by percentage of basic research.

**Figure 11.**
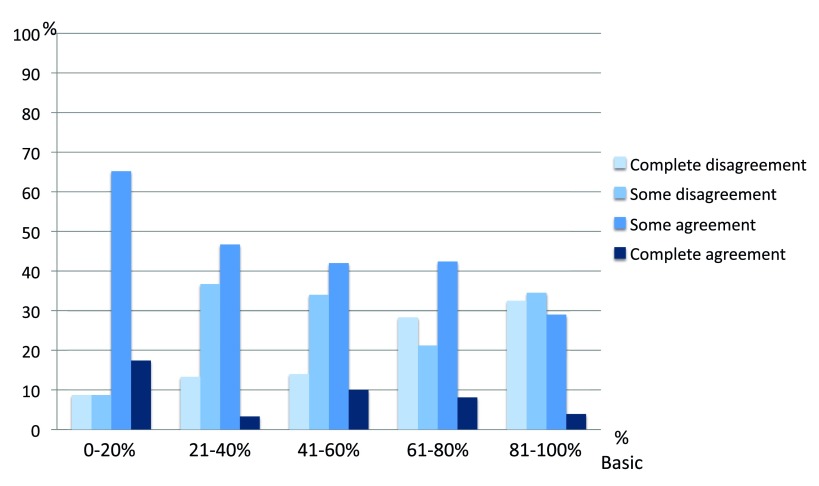
Written proposals about basic biological/biomedical research generally contain a section discussing potential future health benefits. These sections increase the likelihood that a project benefits future Public health. Principal investigators ordered by percentage of basic research.

For PIs, there was a negative correlation between their level of involvement in basic research and their level of agreement with the statement “although it is difficult to assess the potential future health benefits to society from basic biological/biomedical research as described in written proposals, some degree of estimation is always possible” although most PIs, even those with the highest involvement in basic research, were in agreement ( Figure 10). There was also a negative correlation between the degree of involvement in basic research and the statement “written proposals about basic biological/biomedical research generally contain a section discussing potential future health benefits. These sections increase the likelihood that a project benefits future public health.” Here, over two thirds of the PIs with the highest involvement in basic research were in disagreement ( Figure 11).

**Figure 12.**
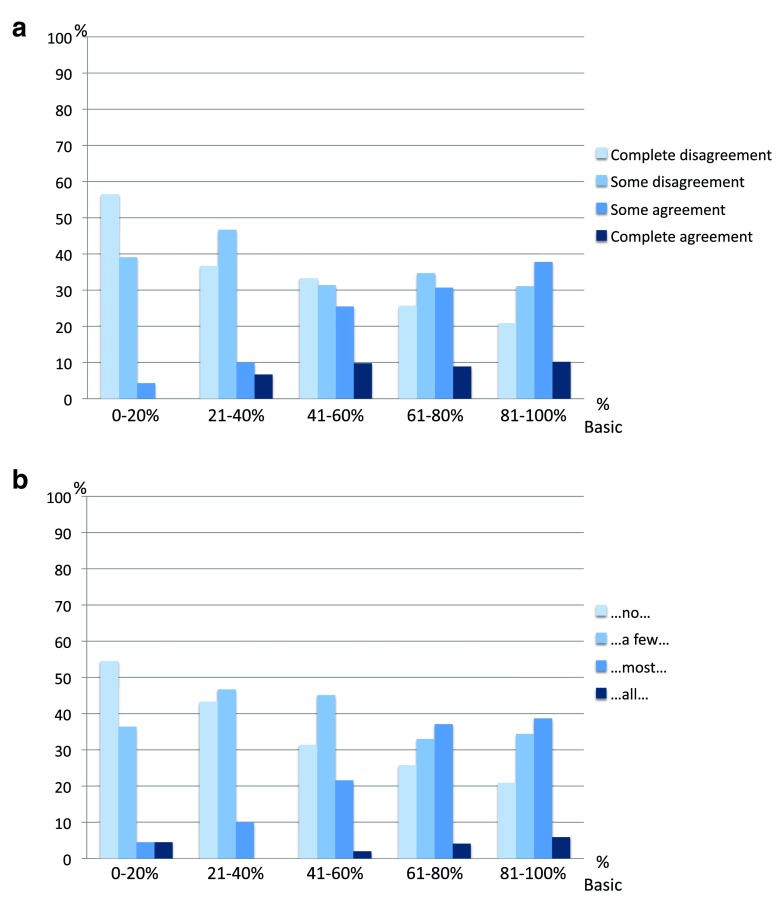
( **a**) Writing the sections discussing potential future health benefits takes too much time. ( **b**) The sections discussion potential future health benefits should be eliminated for …………. grants. Principal investigators ordered by percentage of basic research.

Among PIs, more involvement in basic research led to more support for the statement “writing the sections discussing potential future health benefits takes too much time” ( Figure 12a) and more support for the idea that “the sections discussing potential future health benefits” should be eliminated for at least a subset of grants. This latter opinion was expressed by fewer than 50% of PIs with the least involvement in basic research but by almost 80% of PIs with the highest involvement in basic research ( Figure 12b).

Overall, the results show that most of basic scientists believe that some degree of assessment of the health benefit potential of basic biological or biomedical research is possible but that the current policy requiring the discussion of this potential in written research proposals is not very effective and should be eliminated for at least a portion of the grants, if not most or all of them.

## Soft policies to increase the public health potential of basic research and the satisfaction of scientists

We tested scientists’ opinions concerning six soft policies that we designed based on the results of Study 1. These policies are intended to increase the public health potential of basic investigations and improve the work satisfaction of the basic scientists.

These are the policies:

A. “Locate more basic research laboratories inside or in close proximity of hospitals.”

B. “Organize more educational and discussion meetings between scientists and the general public or patient associations. Acknowledge participating scientists during grant assignments, promotion, hiring etc.”

C. “Promote more seminars and academic discussion concerning the purpose of scientific research and the role of scientists in the society. Acknowledge participating scientists during grant assignments, promotion, hiring etc.”

D. “Promote more seminars and academic discussion about the concept and definition of basic research. Acknowledge participating scientists during grant assignments, promotion, hiring etc.”

E. “Have ethics consultation services for scientists inside research institutes, with easily accessible information about these services.”

F. “Provide recognition to basic scientists who have contributed to acquiring key knowledge that leads to tangible health benefits by requiring a "basic bibliography" of seminal basic research articles for each new drug or other biological application.”

**Figure 13.**
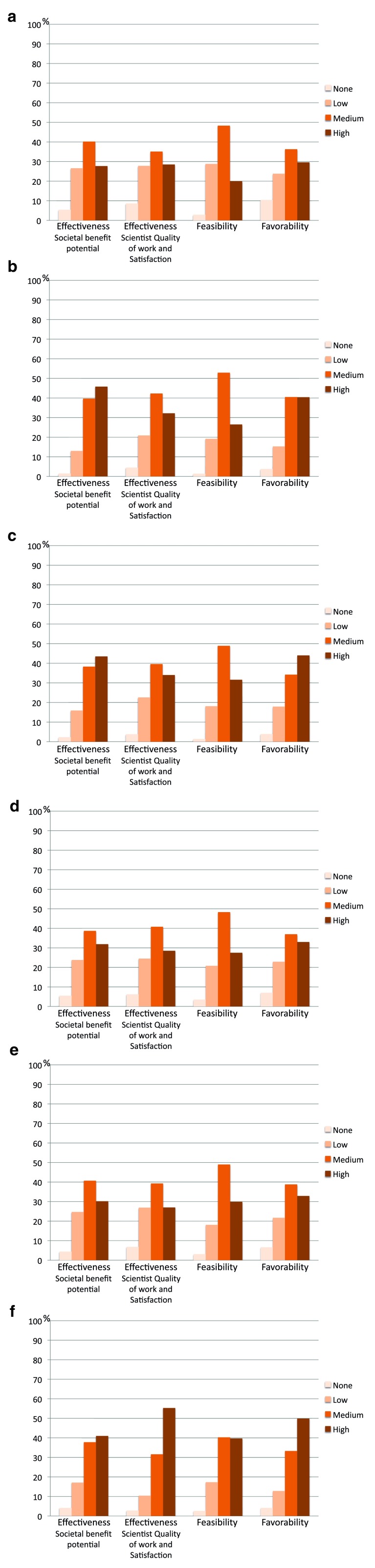
( **a**) POLICY A “Locate more basic research laboratories inside or in close proximity of hospitals”. ( **b**) POLICY B “Organize more educational and discussion meetings between scientists and the general public or patient associations. Acknowledge participating scientists during grant assignments, promotion, hiring etc.” ( **c**) POLICY C “Promote more seminars and academic discussion concerning the purpose of scientific research and the role of scientists in the society. Acknowledge participating scientists during grant assignments, promotion, hiring etc.” ( **d**) POLICY D “Promote more seminars and academic discussion about the concept and definition of basic research. Acknowledge participating scientists during grant assignments, promotion, hiring etc.” (For example, should basic research be conceptualized as purely curiosity-driven, or could basic scientists also consider future indirect practical benefits of their research?). ( **e**) POLICY E “Have ethics consultation services for scientists inside research institutes, with easily accessible information about these services”. ( **f**) POLICY F “Provide recognition to basic scientists who have contributed to acquiring key knowledge that leads to tangible health benefits by requiring a “basic bibliography” of seminal basic research articles for each new drug or other biological application”.

**Figure 14.**
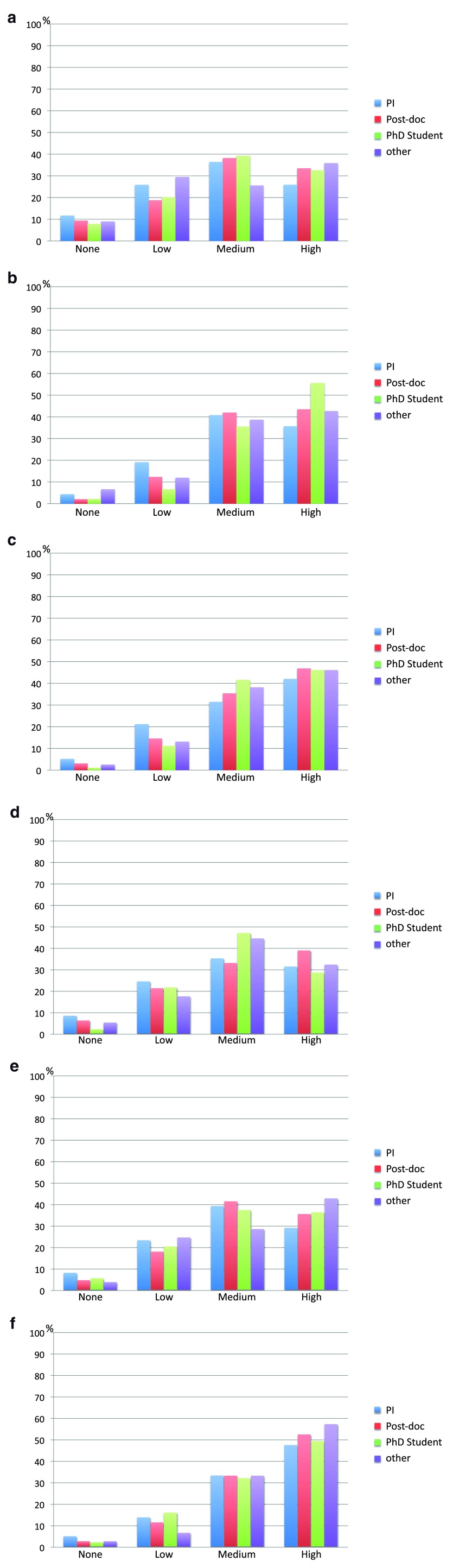
( **a**) Favorability for POLICY A “Locate more basic research laboratories inside or in close proximity of hospitals”. ( **b**) Favorability for POLICY B “Organize more educational and discussion meetings between scientists and the general public or patient associations. Acknowledge participating scientists during grant assignments, promotion, hiring etc.” ( **c**) Favorability for POLICY C “Promote more seminars and academic discussion concerning the purpose of scientific research and the role of scientists in the society. Acknowledge participating scientists during grant assignments, promotion, hiring etc.” ( **d**) Favorability for POLICY D “Promote more seminars and academic discussion about the concept and definition of basic research. Acknowledge participating scientists during grant assignments, promotion, hiring etc.” (For example, should basic research be conceptualized as purely curiosity driven, or could basic scientists also consider future indirect practical benefits of their research?). ( **e**) Favorability for POLICY E “Have ethics consultation services for scientists inside research institutes, with easily accessible information about these services”. ( **f**) Favorability for POLICY F “Provide recognition to basic scientists who have contributed to acquiring key knowledge that leads to tangible health benefits by requiring a “basic bibliography” of seminal basic research articles for each new drug or other biological application”.

Respondents were asked to evaluate these policies on four criteria: the policy’s effectiveness in generating (a) societal benefit, (b) scientists’ work satisfaction, along with the policy’s (c) feasibility and (d) overall favorability. Respondents were asked to evaluate these policies using four scores: “none” (score 1), “low” (score 2), “medium” (score 3), “high” (score 4). The vast majority of scientists judged all six policies to have at least some degree of effectiveness, feasibility and favorability, with a substantial proportion of scientists giving scores of 3 or 4. The policies that scored highest with regard to the societal benefit were B (score 3.3), C (score 3.2) and F (score 3.2). The policy that scored highest with regard to the scientist satisfaction was F (score 3.4). The policies that scored highest with regard to feasibility were F (score 3.2), C (score 3.1) and E (score 3.1). The policies that scored highest with regard to overall favorability were F (score 3.3), B (score 3.2) and C (score 3.2). Policy F had the highest percentage of “high” responses (scores 4) (46.5%) and the highest percentage of “high” responses in terms of overall favorability (49.9%) ( Figures 13a–f). Role of the respondent (i.e., PI, post-doc) did not substantially affect the favorability of these options ( Figures 14a–f).

**Figure 15.**
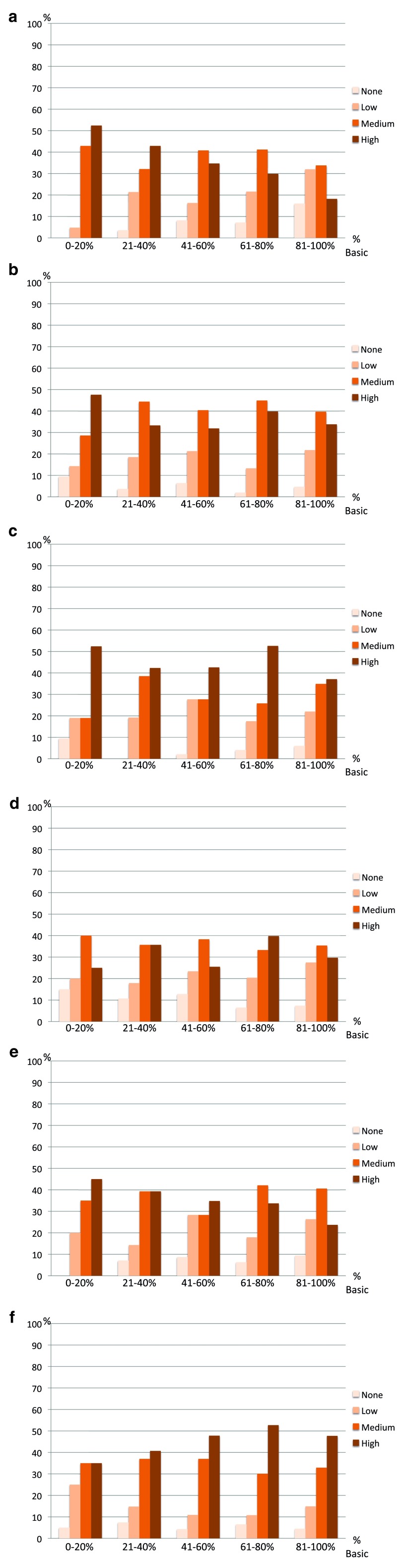
( **a**) Favorability for POLICY A “Locate more basic research laboratories inside or in close proximity of hospitals”. ( **b**) Favorability for POLICY B “Organize more educational and discussion meetings between scientists and the general public or patient associations. Acknowledge participating scientists during grant assignments, promotion, hiring etc.” ( **c**) Favorability for POLICY C “Promote more seminars and academic discussion concerning the purpose of scientific research and the role of scientists in the society. Acknowledge participating scientists during grant assignments, promotion, hiring etc.” ( **d**) Favorability for POLICY D “Promote more seminars and academic discussion about the concept and definition of basic research. Acknowledge participating scientists during grant assignments, promotion, hiring etc.” (For example, should basic research be conceptualized as purely curiosity driven, or could basic scientists also consider future indirect practical benefits of their research?). ( **e**) Favorability for POLICY E “Have ethics consultation services for scientists inside research institutes, with easily accessible information about these services”. ( **f**) Favorability for POLICY F “Provide recognition to basic scientists who have contributed to acquiring key knowledge that leads to tangible health benefits by requiring a “basic bibliography” of seminal basic research articles for each new drug or other biological application”. (Principal Investigators ordered by percentage of basic research).

**Figure 16.**
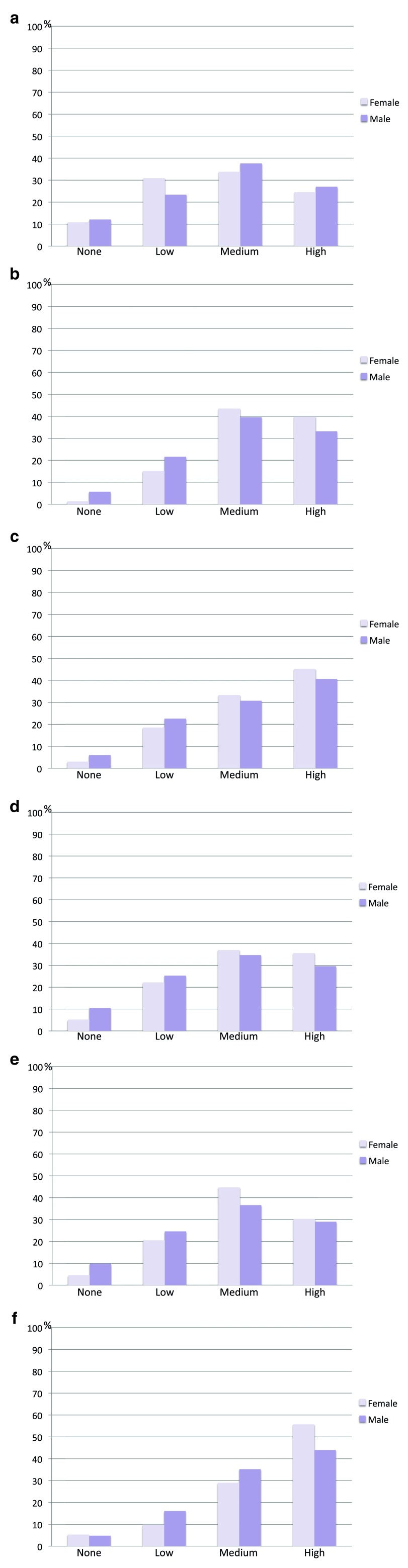
( **a**) Favorability for POLICY A “Locate more basic research laboratories inside or in close proximity of hospitals”. ( **b**) Favorability for POLICY B “Organize more educational and discussion meetings between scientists and the general public or patient associations. Acknowledge participating scientists during grant assignments, promotion, hiring etc.” ( **c**) Favorability for POLICY C “Promote more seminars and academic discussion concerning the purpose of scientific research and the role of scientists in the society. Acknowledge participating scientists during grant assignments, promotion, hiring etc.” ( **d**) Favorability for POLICY D “Promote more seminars and academic discussion about the concept and definition of basic research. Acknowledge participating scientists during grant assignments, promotion, hiring etc.” (For example, should basic research be conceptualized as purely curiosity driven, or could basic scientists also consider future indirect practical benefits of their research?). ( **e**) Favorability for POLICY E “Have ethics consultation services for scientists inside research institutes, with easily accessible information about these services”. ( **f**) Favorability for POLICY F “Provide recognition to basic scientists who have contributed to acquiring key knowledge that leads to tangible health benefits by requiring a “basic bibliography” of seminal basic research articles for each new drug or other biological application”. (Principal Investigators and Gender).

For PIs, in terms of degree of involvement in basic research, there were substantial differences in favorability only in relation to policy A, for which there was a small negative correlation (R ^2^ = 0.986) ( Figures 15a–f). With regard to the influence of the gender of the PIs, there was a slight increase in favorability of female PIs for policy B–F and in favorability of male PIs for policy A ( Figures 16a–f).

## Conclusions

Ten main results deserve some emphasis:

• Basic scientists are strongly motivated not only by “satisfaction of curiosity” and “from solving puzzling problems” but also by the possible future practical benefits to society.
• There is a positive correlation between involvement in basic research and the importance of motivation from “pure advancement of knowledge,” “satisfaction of curiosity,” and “satisfaction from solving puzzling problems.”
• There is a negative correlation between involvement in basic research and motivation for the “health benefit to society.”
• PIs are more motivated than other types of investigators from “pure advancement of knowledge,” “satisfaction of curiosity,” and “satisfaction from solving puzzling problems.”
• While money is not a powerful motivation, prestige is an important to moderately important motivation for nearly half of PIs.
• Almost all scientists think that it is possible to ponder possible future applications of basic investigations without compromising the basic nature of the research.
• On the other hand, most basic scientists disfavor policies mandating the discussion of these practical applications in research proposals.
• There is a positive correlation between PIs’ involvement in basic research and their rejection of the requirement of discussing the health benefit potential in research proposals.
• There is a large consensus on the effectiveness, feasibility and favorability for all six soft policies designed to increase the public health potential of basic research and the scientist satisfaction.
• Among these six policies, those entailing the organization of more meetings between scientists and the general public, the organization of more academic discussion about the role of scientists in the society, and the implementation of a “basic bibliography” for each new approved drug received the highest approval rates.

## Discussion

This study is a follow-up to a preliminary study of a smaller sample of basic scientists working at a single institution (Harvard University) in a single geographical area (the Boston area in the United States). The results of the preliminary study have been recently published alongside an in-depth analysis of the related literature and conceptual framework ^20^. Based on the results of that survey, we expanded the study to include questions on six specific soft policies and a larger sample of scientists working at different institutions in four geographical areas (see Figures S19–S26 for differences between locations) in three countries.

The current study substantially confirmed what we had discovered in Study 1. In particular, the new study confirmed what we had previously observed with regard to basic scientists’ motivations and conceptualization of basic research. Moreover, it further reinforces the notion that, while some estimate of the public health potential of basic investigations is always possible, basic scientists believe that the requirement of discussing this potential in research proposals is not effective and should be eliminated for at least a portion of the grants (see also Figures S27–S30). The six proposed “nudge”-based policies were judged positively in terms of their future public health impact, scientist work satisfaction, and feasibility, with policies B, C and F receiving the highest approval ratings.

This study has several strengths. First, we gathered data from a large and diverse population of basic scientists. Second, we analyzed responses not only with respect to the role of the scientist (e.g., PI, post-doc) but also with the self-reported (from 0 to 100%) level of involvement with basic research. As far as we could determine, this has not been done before. Third, the survey has a depth and level of detail rarely seen in surveys of the motivations and perspectives of biological and biomedical scientists. Finally, and most important, this study provides information on specific policies, some of these new policies (B, C, D, F), and the findings can be used by policymakers to improve the governance of basic research.

The study has also weaknesses. First the survey results may not be fully representative of the views of all basic biomedical and biological scientists. Although based on a large and multinational sample, it still only presents the views of scientists in three countries. Moreover, since we focused our study mainly on PIs and post-docs, this study is less representative of the opinions of students or other types of scientists such as staff scientists or research technicians. In addition, although we had a large sample, only 11% of possible respondents answered the survey. Finally, although most of the surveyed scientists were positive about the effectiveness and feasibility of the proposed policies, that alone does not ensure these policies will in fact be effective and/or feasible. Similarly, it does not imply that other parts of the society (e.g. the general public and the policymakers) have the same views.

We hope the information and discussion provided in this paper will be useful to scholars, policymakers and advocates. We encourage them to foster the discussion and work for the implementation of policies that can benefit both society and science. The results provided in this paper suggest that the proposed policies are well grounded in the motivations and perspectives of the basic bioscientists and have their approval. We believe this is an important asset with respect to what would be the actual effectiveness of these policies and the potential for implementation.

## Data availability

The data referenced by this article are under copyright with the following copyright statement: Copyright: © 2016 Sorrentino C et al.

Data associated with the article are available under the terms of the Creative Commons Zero "No rights reserved" data waiver (CC0 1.0 Public domain dedication). 

*F1000Research*: Dataset 1. Questions and responses of the survey, 10.5256/f1000research.7683.d110888 ^21^

## Supplementary material

Figures S1–S6. Data on the invitations to the questionnaire.

Figures S7–S11. Data on the response rates of the questionnaire.

Figures S12–S18. Data describing the sample of scientists participating to the questionnaire.

Figures S19–S26. Data describing the differences in the responses based on geographical location.

Figures S27–S30. Additional data on scientists views on criteria of evaluation and funding of basic research.

Tables showing the detailed percentages of responses for all the figures of the paper ( Figures 1–16).

## References

[1] Ballabeni A Boggio A Hemenway D: Recognizing Basic Science Contributions. *The Scientist.* 2014;28:26–27. Reference Source

[2] Beckwith J Huang F: Should we make a fuss? A case for social responsibility in science. *Nat Biotechnol.* 2005;23(12):1479–1480. 10.1038/nbt1205-1479 16333283

[3] Bornmann L: Measuring the societal impact of research: research is less and less assessed on scientific impact alone--we should aim to quantify the increasingly important contributions of science to society. *EMBO Rep.* 2012;13(8):673–676. 10.1038/embor.2012.99 22777497 PMC3410397

[4] Bush V: Science the endless frontier. *Trans Kans Acad Sci (1903-).*Washington DC: US Government Printing Office.1945;48(3):231–264. 10.2307/3625196

[5] Chalmers I Bracken MB Djulbegovic B: How to increase value and reduce waste when research priorities are set. *Lancet.* 2014;383(9912):156–165. 10.1016/S0140-6736(13)62229-1 24411644

[6] Kuhn T: The Structure of Scientific Revolutions.1962 Reference Source

[7] Ladd JM Lappé MD McCormick JB: The "how" and "whys" of research: life scientists' views of accountability. *J Med Ethics.* 2009;35(12):762–767. 10.1136/jme.2009.031781 19948933 PMC4396621

[8] Merton R: The Sociology of Science: theoretical and empirical investigations.The University of Chicago Press.1973 Reference Source

[9] Sampat BN: Mission-oriented biomedical research at the NIH. *Res Policy.* 2012;41(10):1729–1741. 10.1016/j.respol.2012.05.013

[10] Stokes D: Pasteurs Quadrant: Basic Science and Technological Innovation.Washington DC: Brookings Institution Press.1997 Reference Source

[11] Wilsdon J Wynne B Stilgoe J: The public value of science: or how to ensure that science really matters. London: Demos,2005 Reference Source

[12] Pouliot C Godbout J: Thinking outside the 'knowledge deficit' box. *EMBO Rep.* 2014;15(8):833–835. 10.15252/embr.201438590 24993560 PMC4197039

[13] Rull V: The most important application of science: As scientists have to justify research funding with potential social benefits, they may well add education to the list. *EMBO Rep.* 2014;15(9):919–922. 10.15252/embr.201438848 25135952 PMC4198034

[14] McCormick JB Boyce AM Ladd JM: Barriers to Considering Ethical and Societal Implications of Research: Perceptions of Life Scientists. *AJOB Prim Res.* 2012;3(3):40–50. 10.1080/21507716.2012.680651 22866239 PMC3409664

[15] Besley JC Oh SH Nisbet M: Predicting scientists' participation in public life. *Public Underst Sci.* 2013;22(8):971–987. 10.1177/0963662512459315 23825262

[16] Dubochet J: Teaching scientists to be citizens. It is hard to become a good scientist. It is even harder to become a good citizen. *EMBO Rep.* 2003;4(4):330–332. 10.1038/sj.embor.embor810 12671671 PMC1319166

[17] Saunders C Girgis A Butow P: Beyond scientific rigour: funding cancer research of public value. *Health Policy.* 2007;84(2–3):234–242. 10.1016/j.healthpol.2007.05.002 17573144

[18] Thaler RH Sunstein CR: Nudge: improving decisions about health, wealth, and happiness. New Haven: Yale University Press,2008 Reference Source

[19] Lam A: What motivates academic scientists to engage in research commercialization: ‘Gold’, ‘ribbon’ or ‘puzzle’? *Res Policy.* 2011;40(10):1354–1368. 10.1016/j.respol.2011.09.002

[20] Ballabeni A Boggio A Hemenway D: Policies to increase the social value of science and the scientist satisfaction. An exploratory survey among Harvard bioscientists [version 2; referees: 2 approved]. *F1000Res.* 2014;3:20. 10.12688/f1000research.3-20.v2 24795807 PMC3999931

[21] Scita G Sorrentino C Boggio A: Dataset 1 in: Increasing the public health potential of basic research and the scientist satisfaction. An international survey of bioscientists. *F1000Research.* 2016 Data Source 10.12688/f1000research.7683.1 PMC4909114 27347372

