# Supplementary material for: Increasing both the public health potential of basic research and the scientist satisfaction. An international survey of bio-scientists
Source: F1000Res. 2016 Jun 1;5:56. Originally published 2016 Jan 12. [Version 2] doi: 10.12688/f1000research.7683.2 (PMC4909114; doi:10.12688/f1000research.7683.2)
Supplement: Supplementary file 2 [file f1000research-5-9472-s0001.tgz › 68e49843-9a68-41e7-b12d-79eb11201a6c.pdf]

Invitations: Principal Investigators (%) per Geographical Location

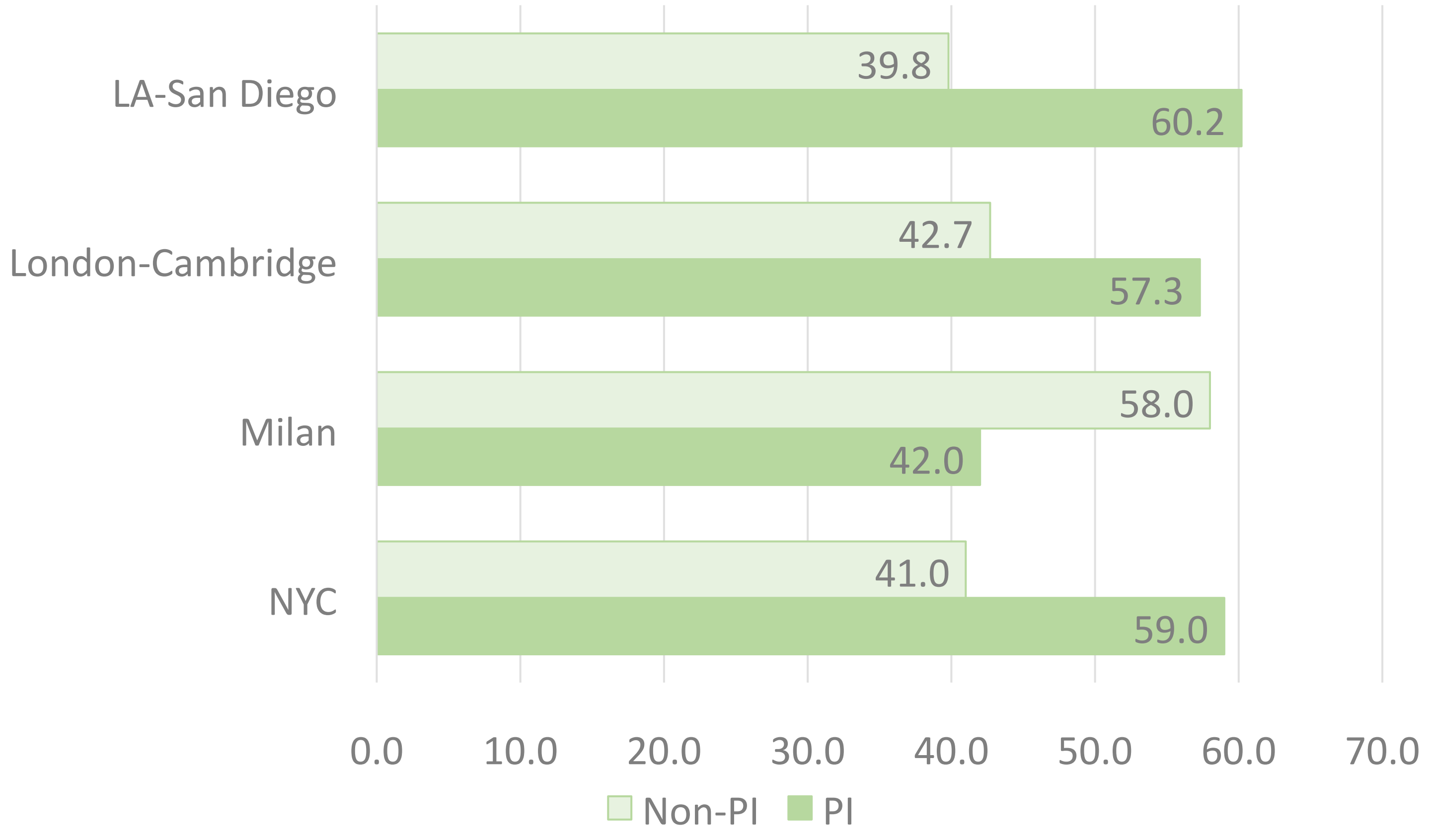

Fig. S1

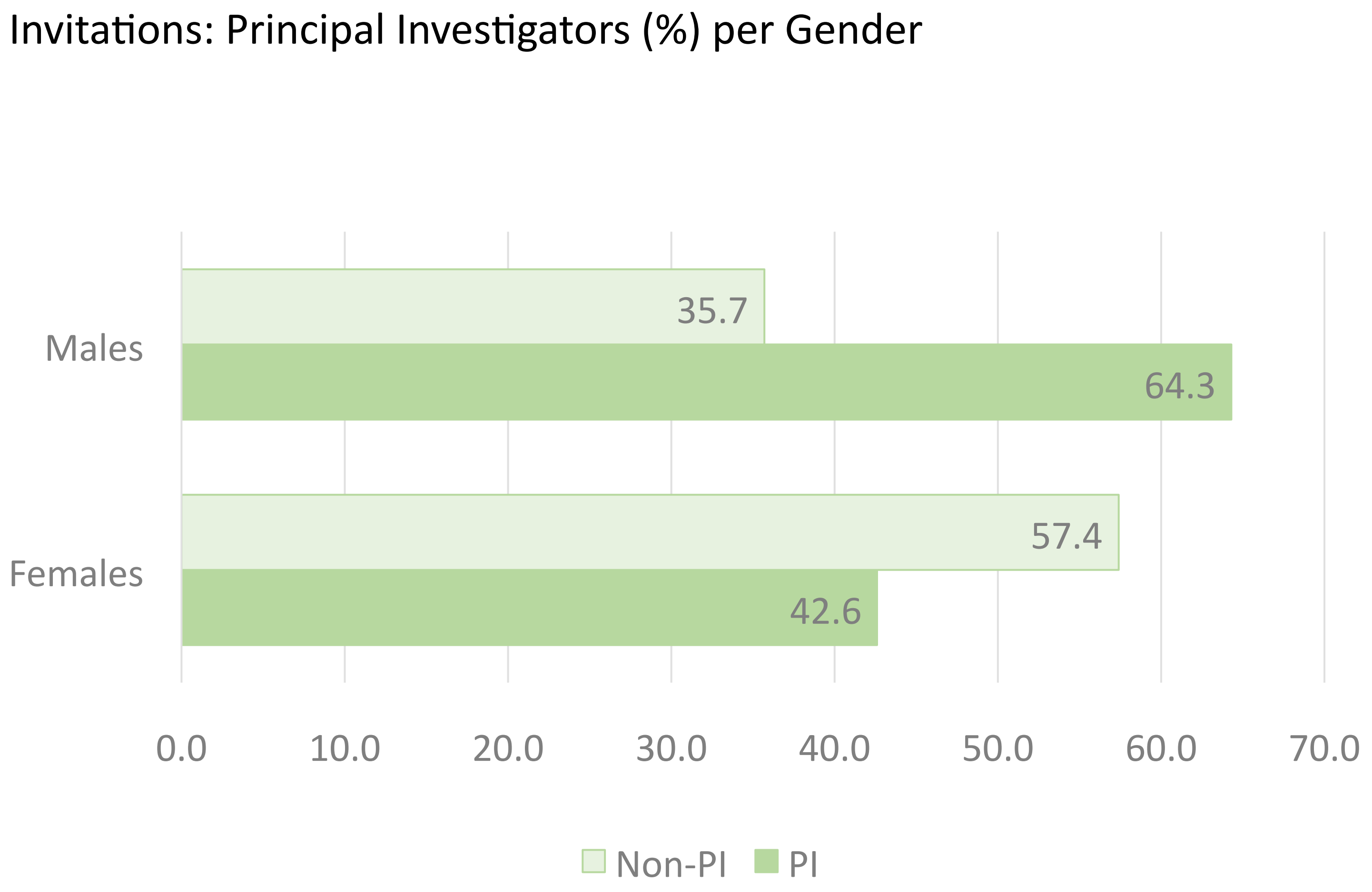

Invitations: Principal Investigators (%) per Gender
Males
Females
35.7
64.3
57.4
42.6
0.0
10.0
20.0
30.0
40.0
50.0
60.0
70.0
Non-PI
PI

Fig. S2

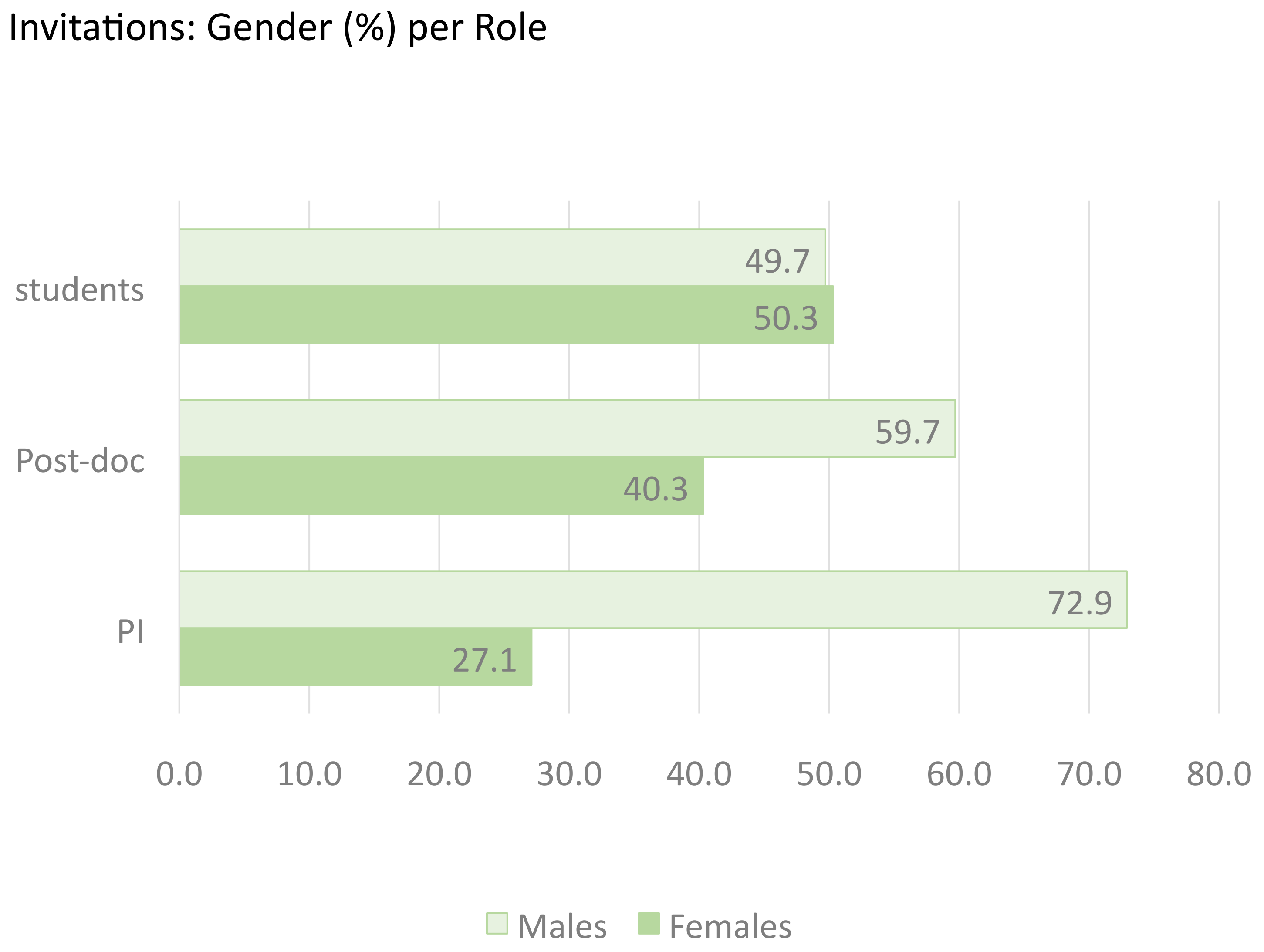
Invitations: Gender (%) per Role
students
Post-doc
PI
49.7
50.3
59.7
40.3
72.9
27.1
0.0
10.0
20.0
30.0
40.0
50.0
60.0
70.0
80.0
Males
Females

Fig. S3

Invitations: Principal Investigators Gender (%) per Geographical Location

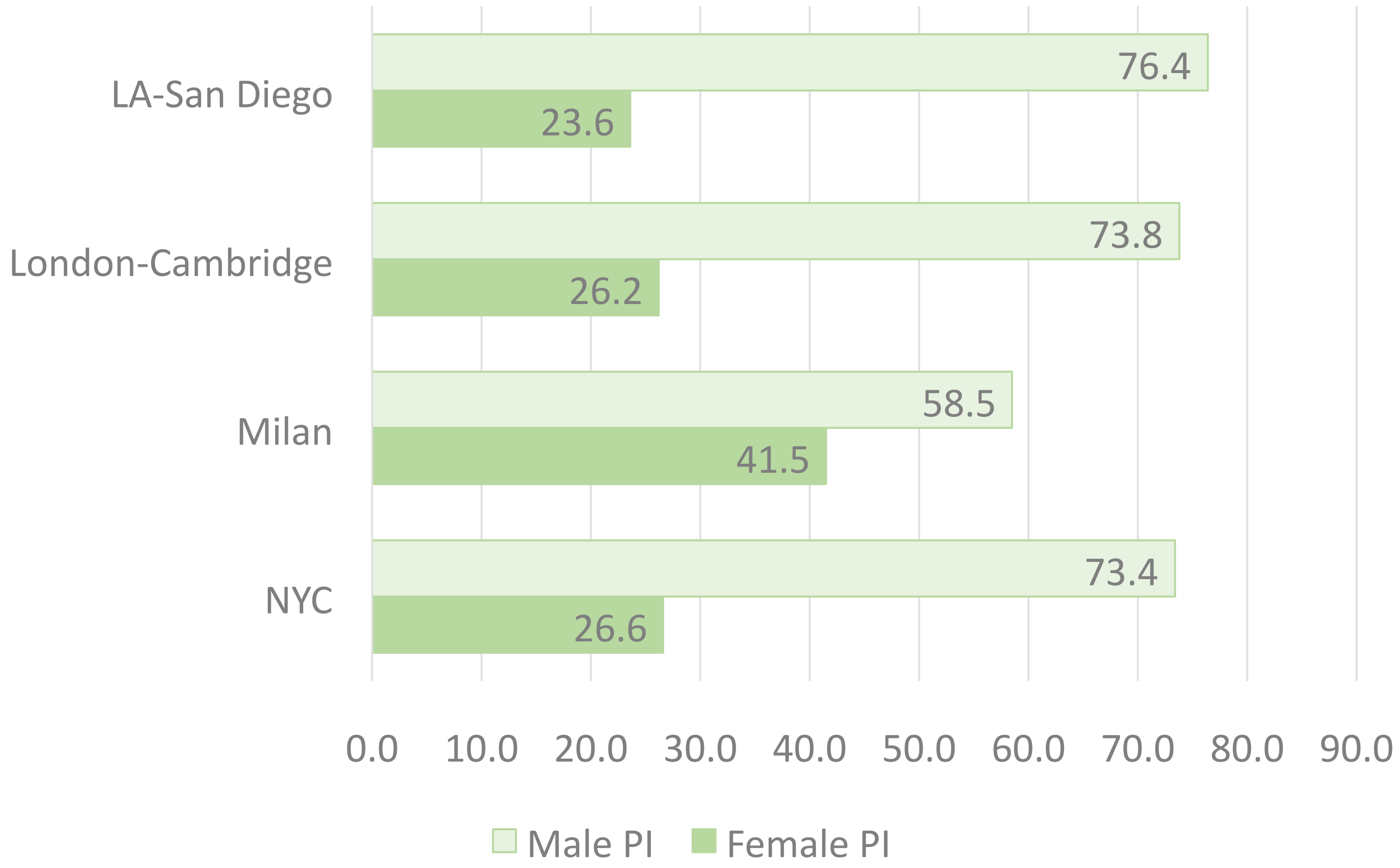

Fig. S4

Invitations: Post-docs Gender (%) per Geographical Location

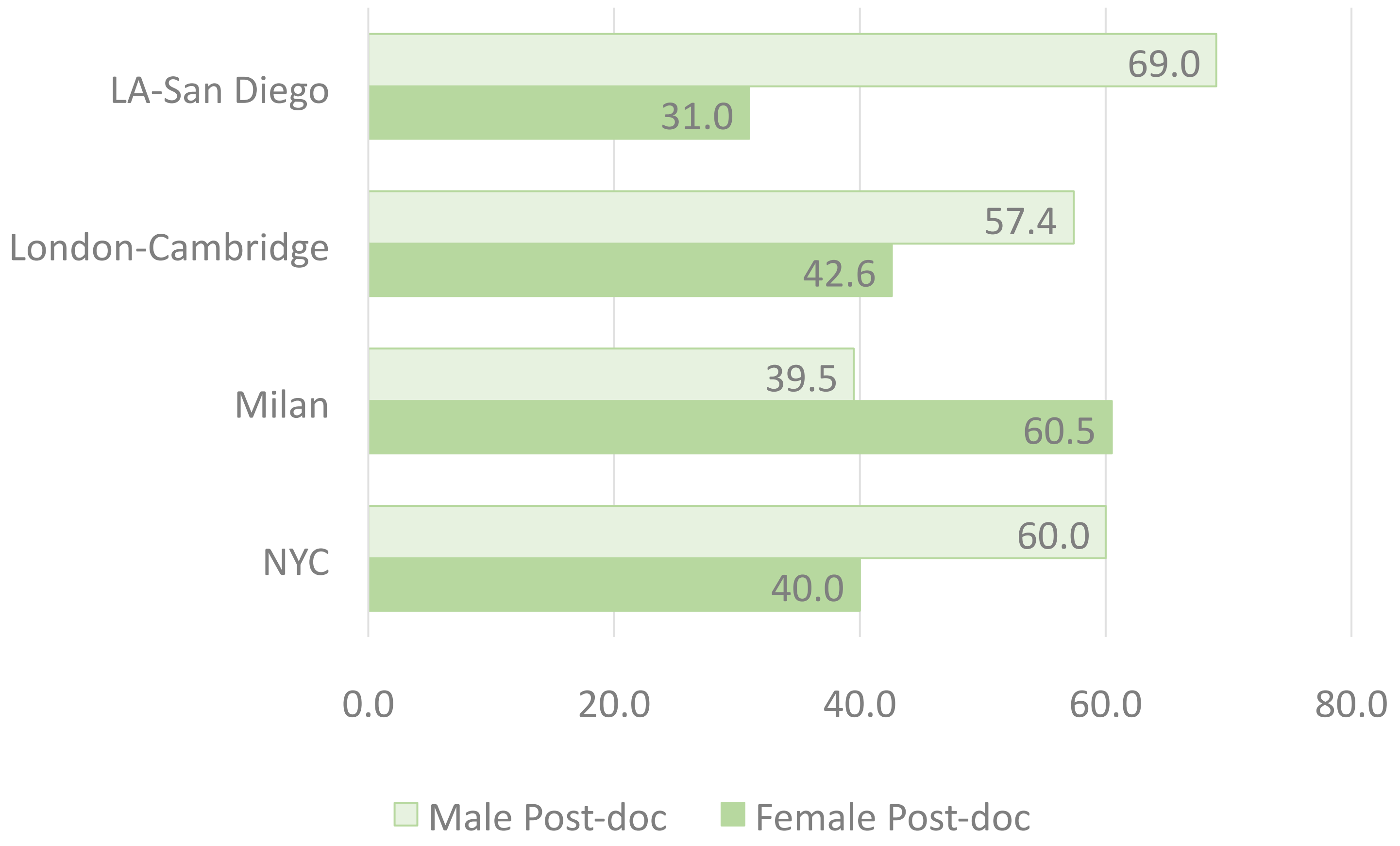

Fig. S5

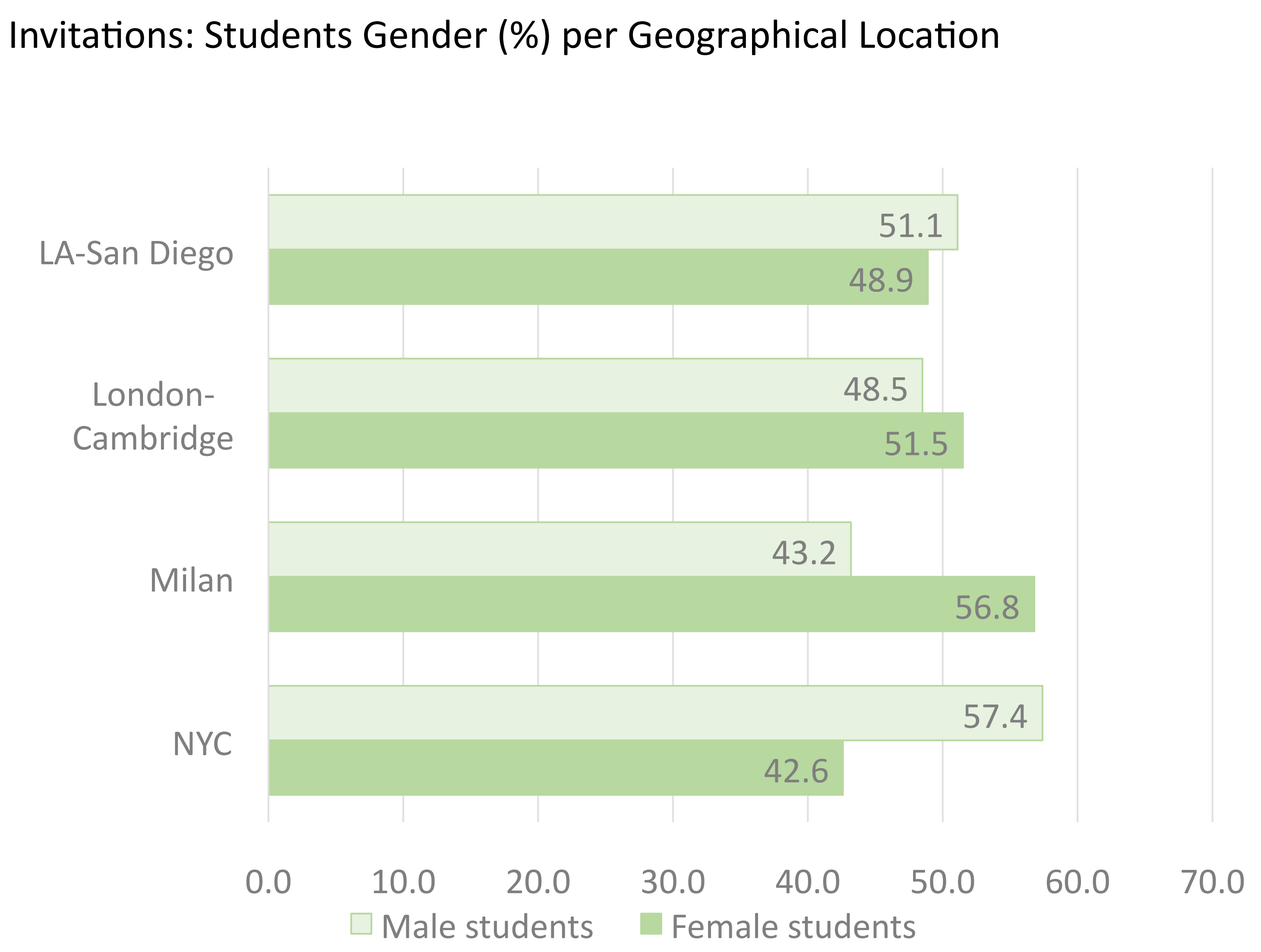
Invitations: Students Gender (%) per Geographical Location
LA-San Diego
51.1
48.9
London-Cambridge
48.5
51.5
Milan
43.2
56.8
NYC
57.4
42.6
0.0
10.0
20.0
30.0
40.0
50.0
60.0
70.0
Male students
Female students

Fig. S6
