# Supplementary material for: Increasing both the public health potential of basic research and the scientist satisfaction. An international survey of bio-scientists
Source: F1000Res. 2016 Jun 1;5:56. Originally published 2016 Jan 12. [Version 2] doi: 10.12688/f1000research.7683.2 (PMC4909114; doi:10.12688/f1000research.7683.2)
Supplement: Supplementary file 3 [file f1000research-5-9472-s0002.tgz › 7f70a4fb-f59d-4754-a174-d99fe9871bf0.pdf]

Response rates: Principal Investigators (%)

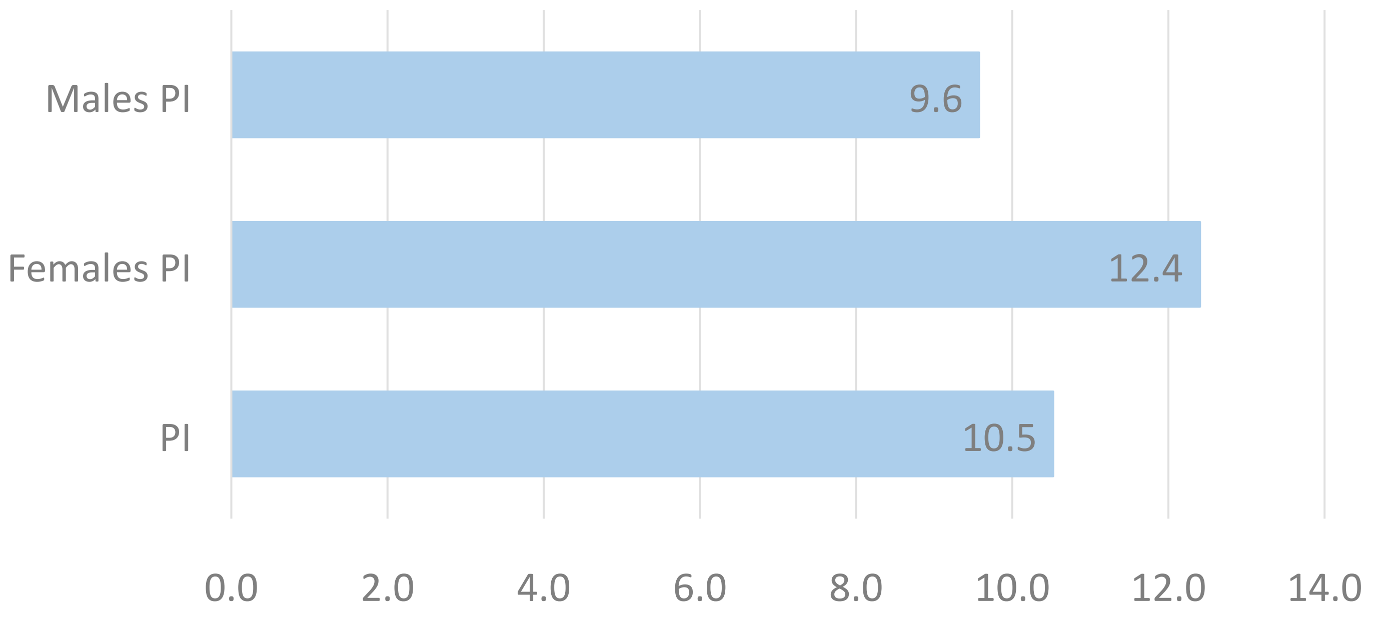

Fig. S7

Response rates: Gender (%)

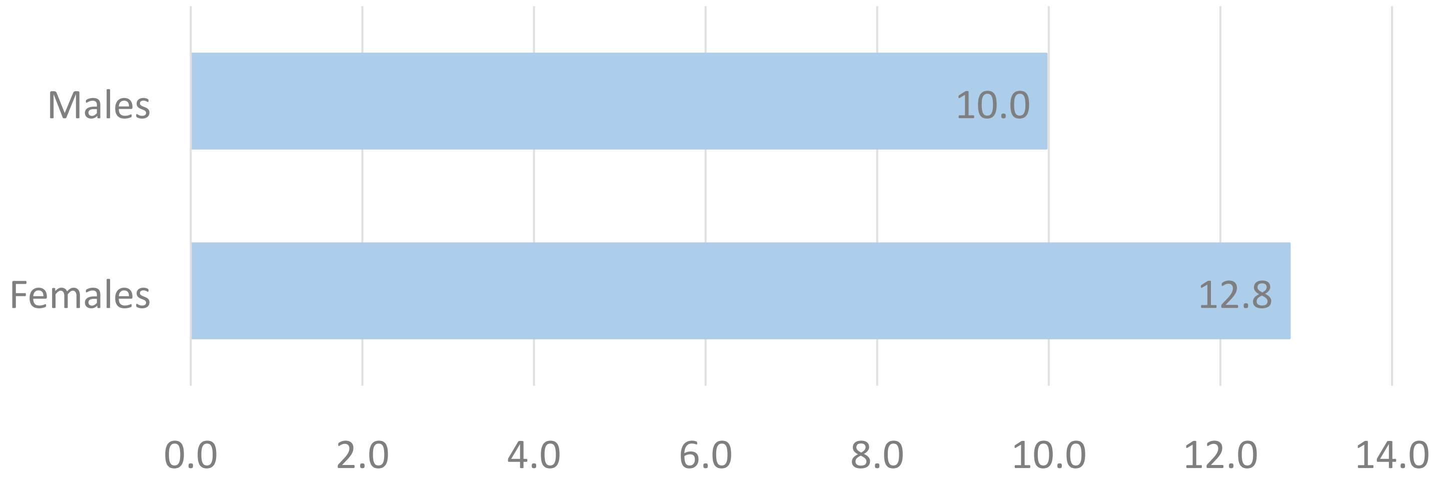

Fig. S8

Response rates: Geographical Locations (%)

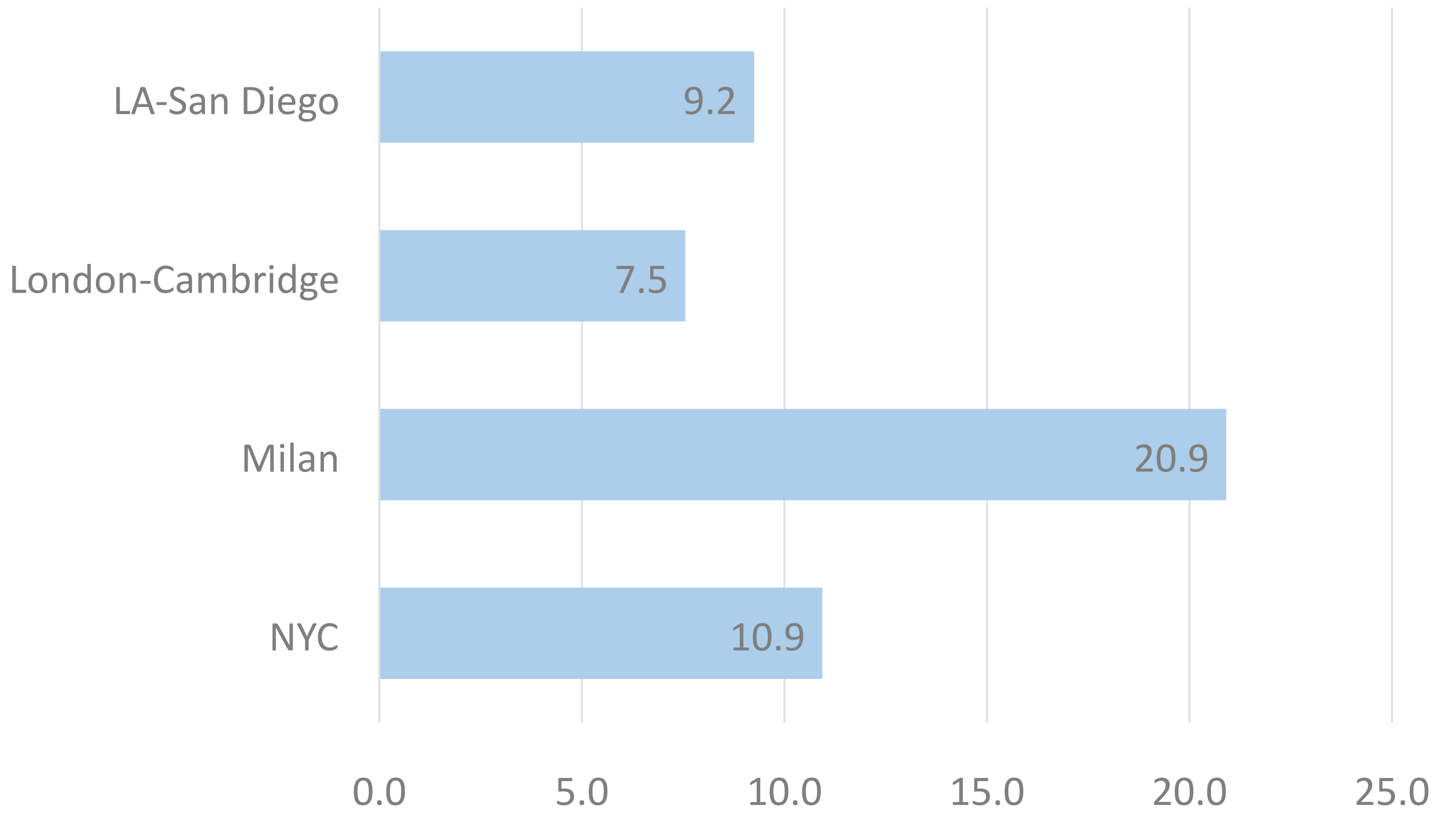

Fig. S9

Response rates: Principal Investigators (%) per Geographical Location

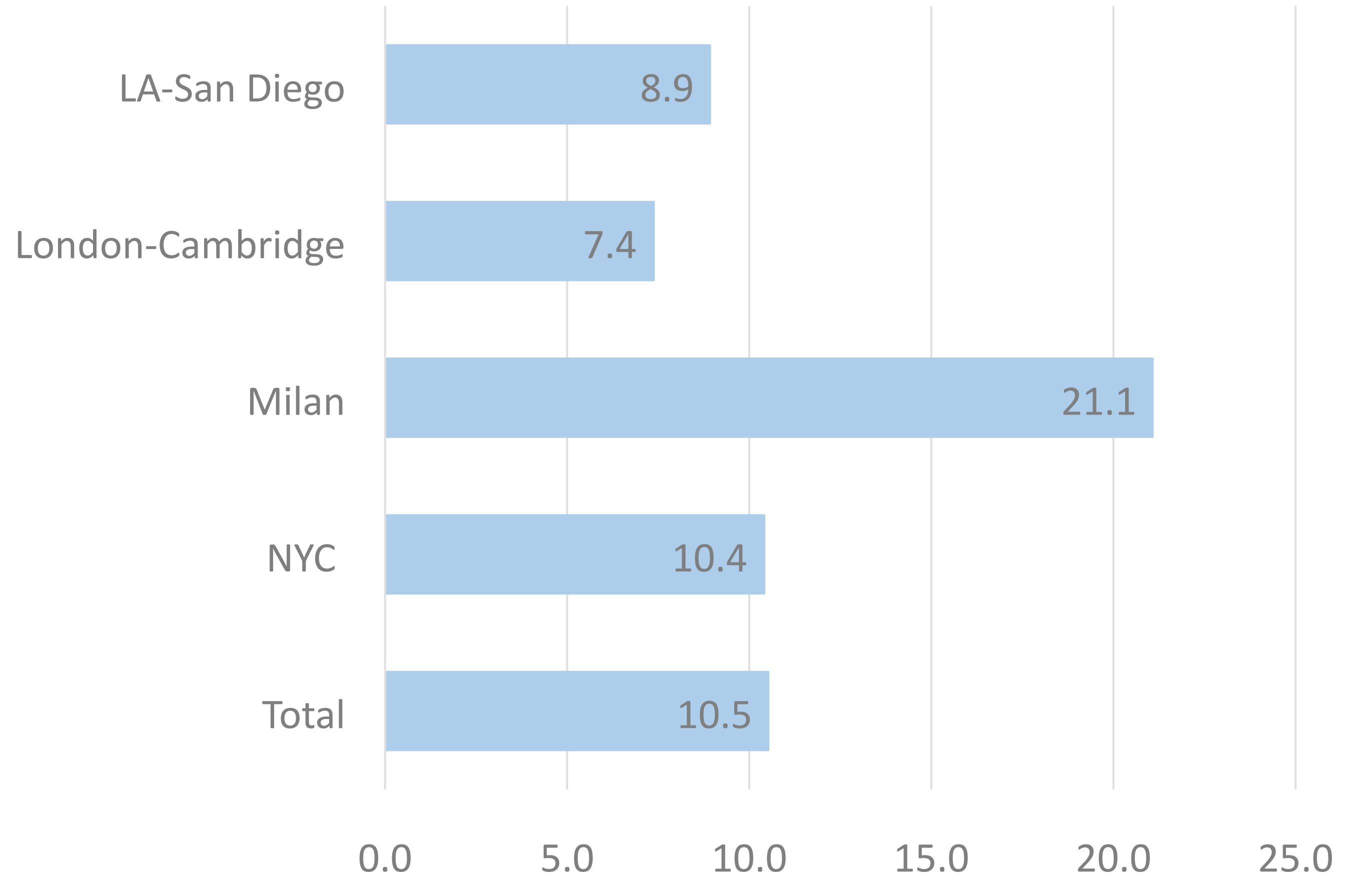

Fig. S10

Response rates: Gender (%) per Geographical Location

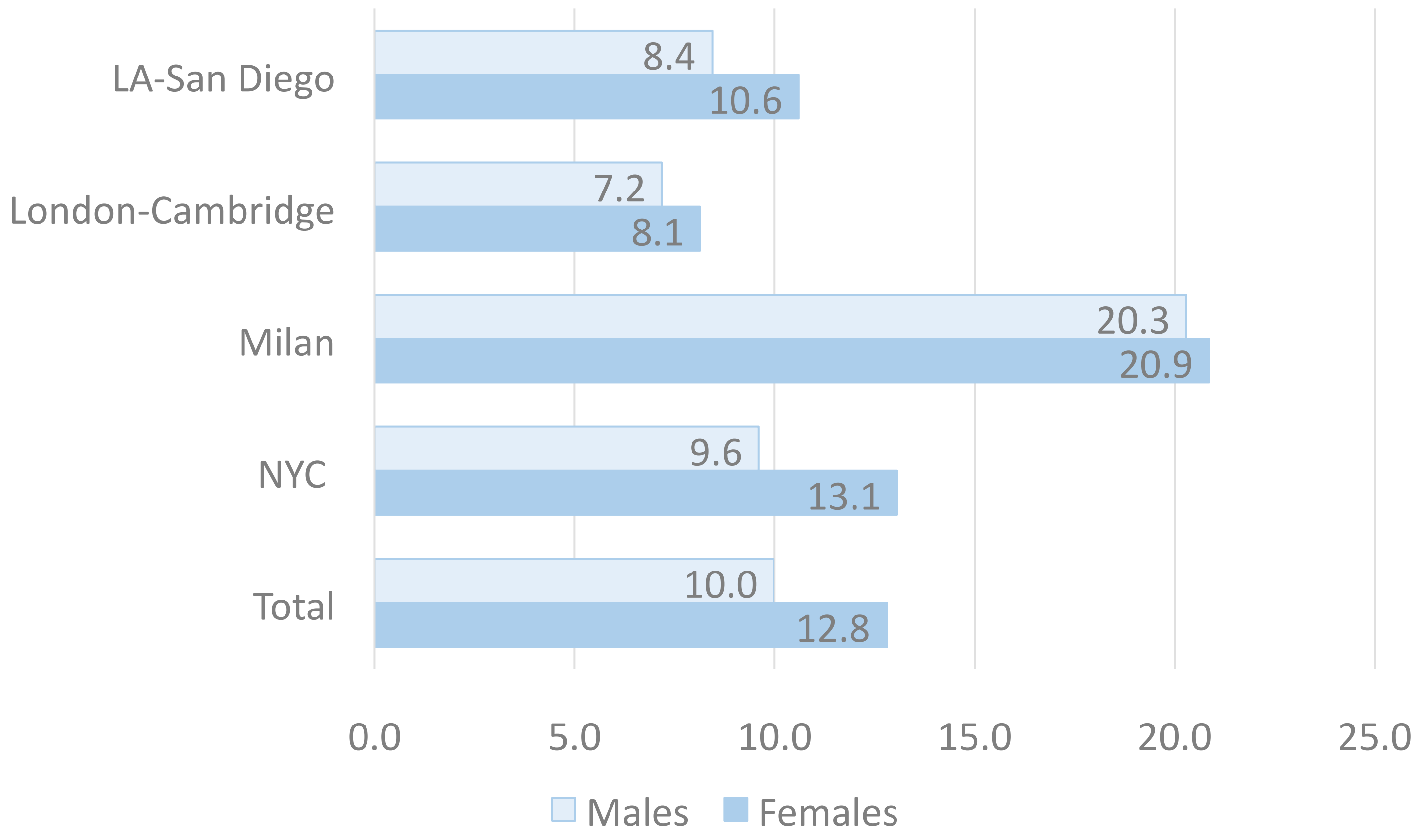

Fig. S11a

Response rates: Gender (%) per Geographical Location for Principal Investigators

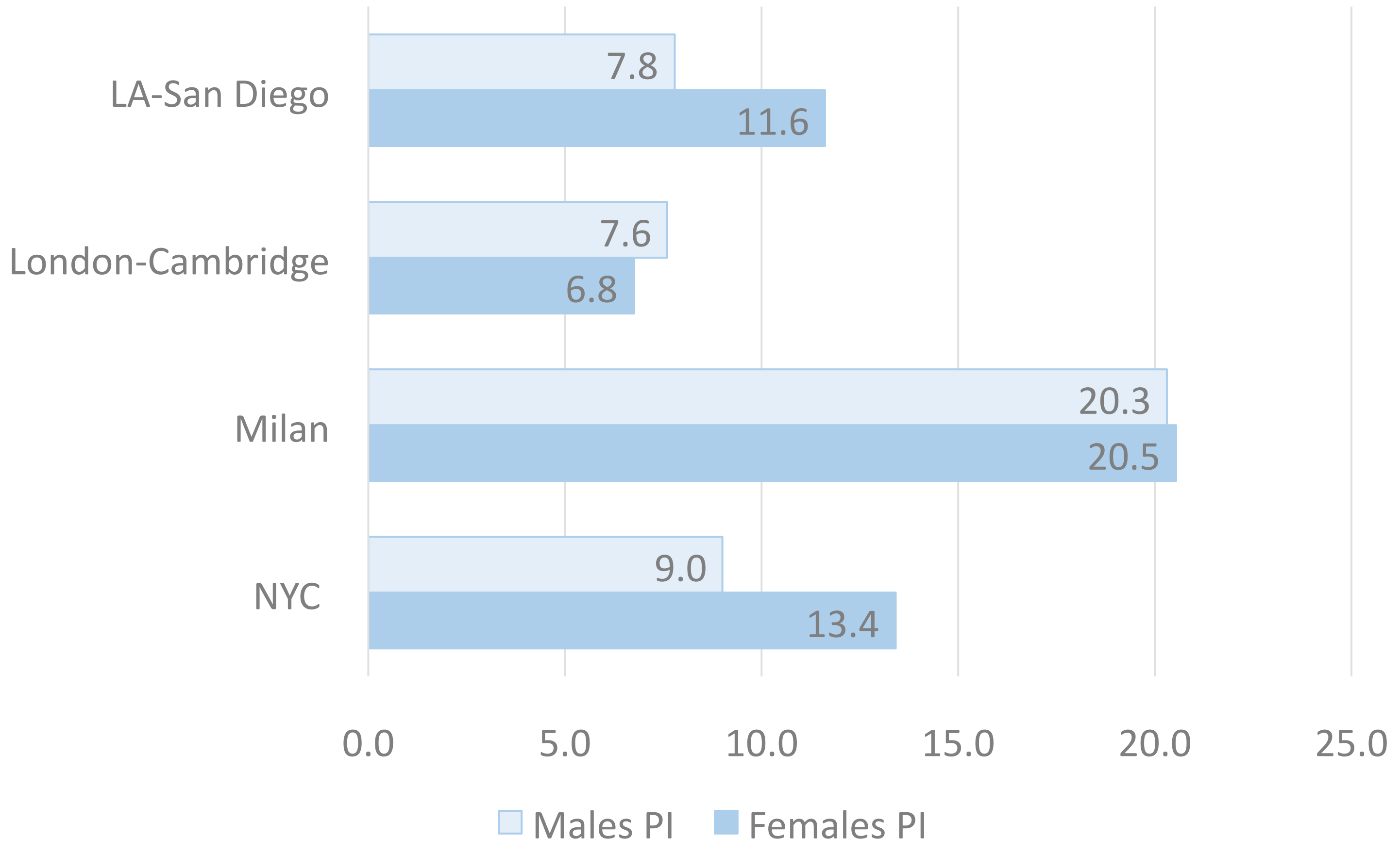

Fig. S11b
