# Supplementary material for: Increasing both the public health potential of basic research and the scientist satisfaction. An international survey of bio-scientists
Source: F1000Res. 2016 Jun 1;5:56. Originally published 2016 Jan 12. [Version 2] doi: 10.12688/f1000research.7683.2 (PMC4909114; doi:10.12688/f1000research.7683.2)
Supplement: Supplementary file 4 [file f1000research-5-9472-s0003.tgz › 8d31952e-b818-4bba-8c17-ff0267abb6e4.pdf]

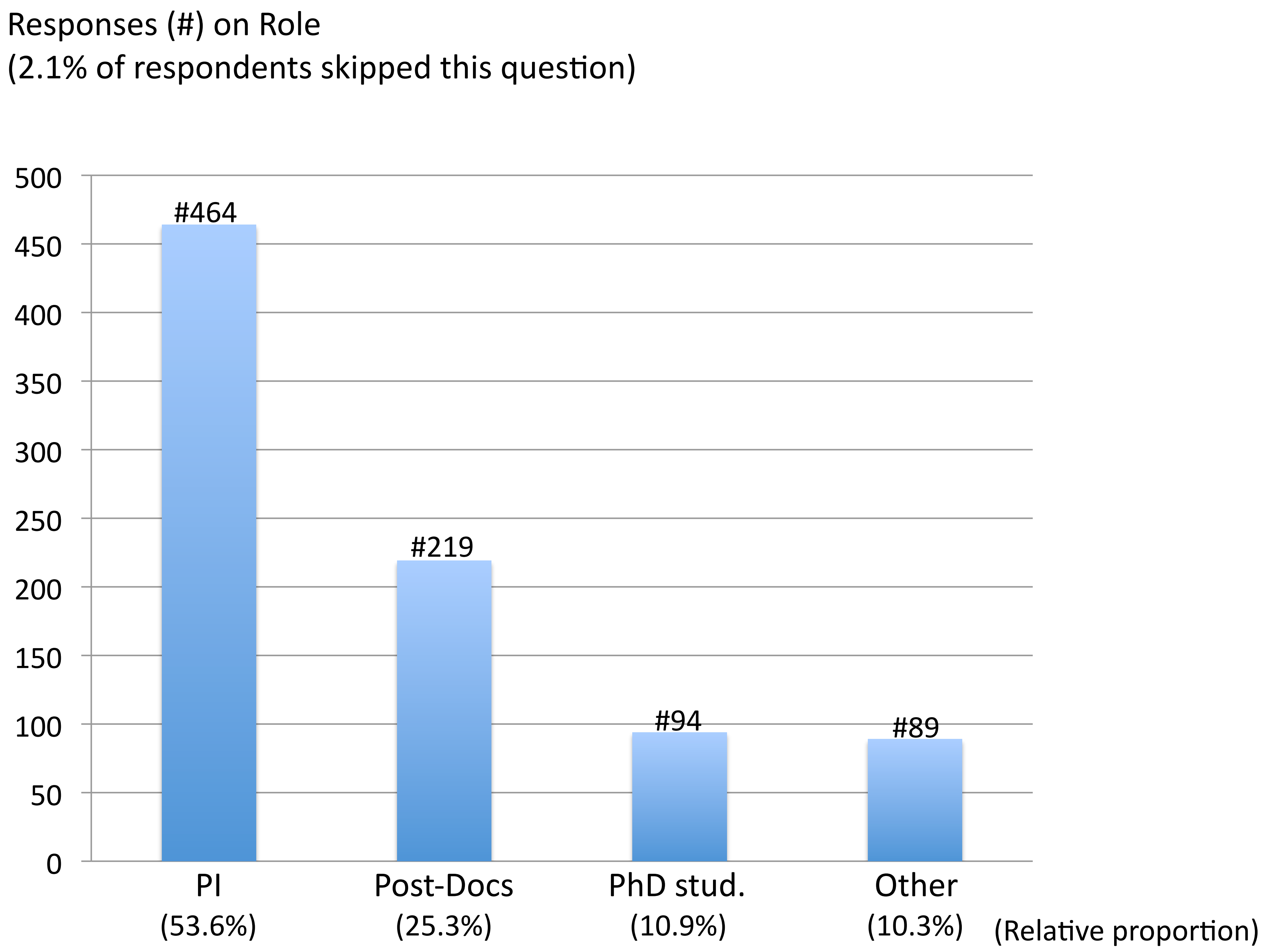

Responses (#) on Role
(2.1% of respondents skipped this question)
500
450
400
350
300
250
200
150
100
50
0
#464
#219
#94
#89
PI
(53.6%)
Post-Docs
(25.3%)
PhD stud.
(10.9%)
Other
(10.3%)
(Relative proportion)

Fig. S12

Responses (#) on Gender
(2.9% of respondents skipped this question)

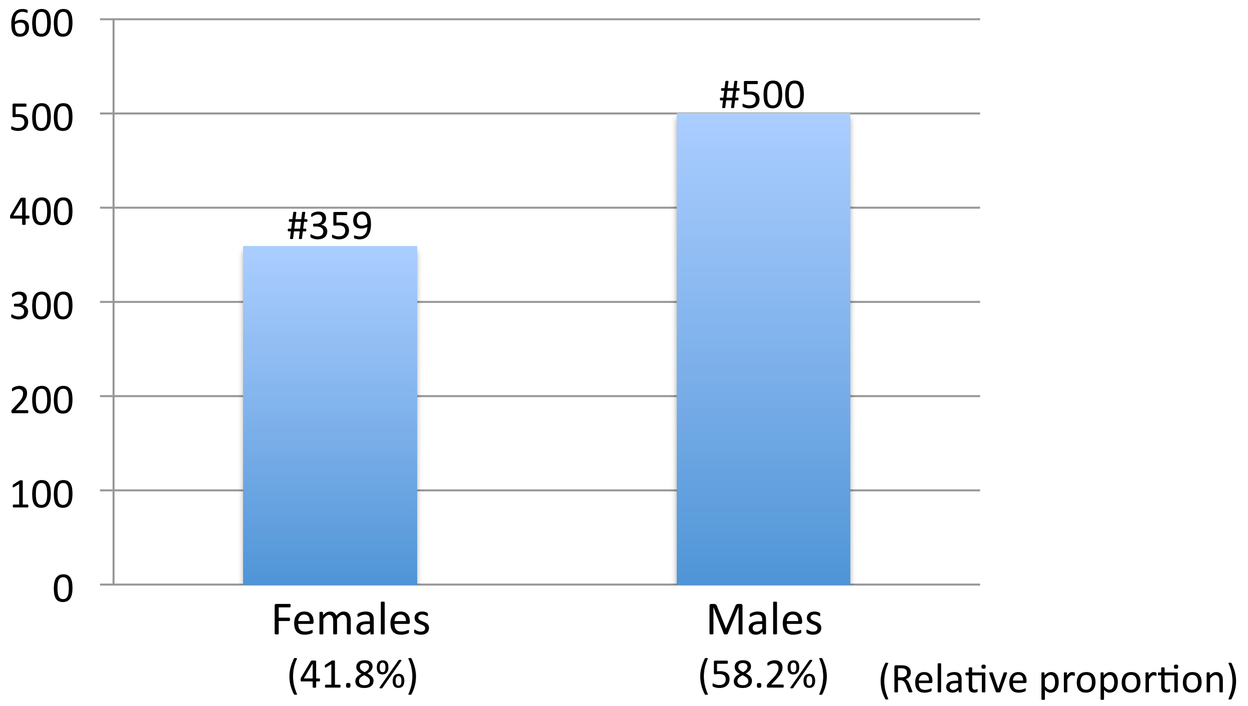

Fig. S13

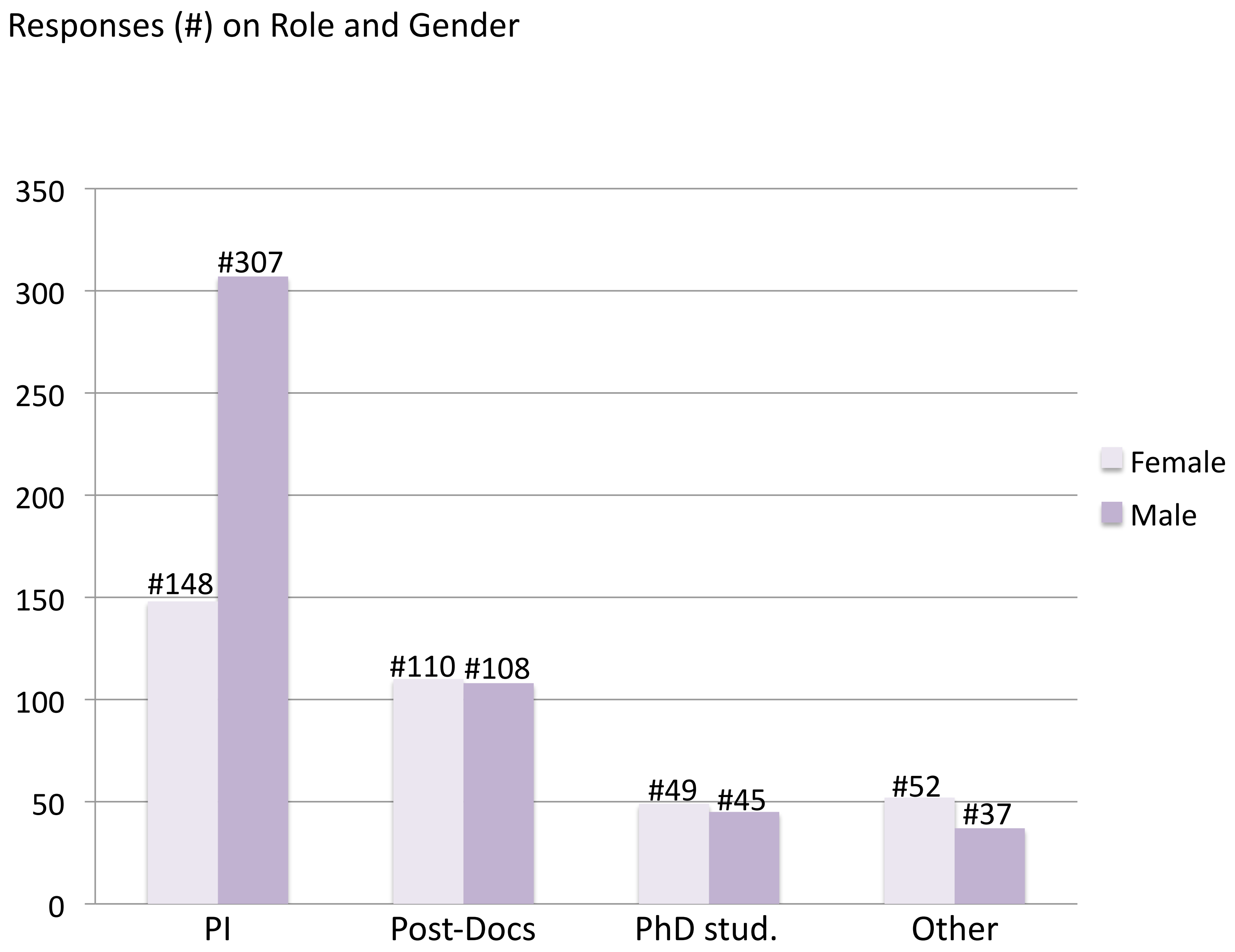
Responses (#) on Role and Gender
350
300
250
200
150
100
50
0
#307
#148
#110
#108
#49
#45
#52
#37
PI
Post-Docs
PhD stud.
Other
Female
Male

Fig. S14

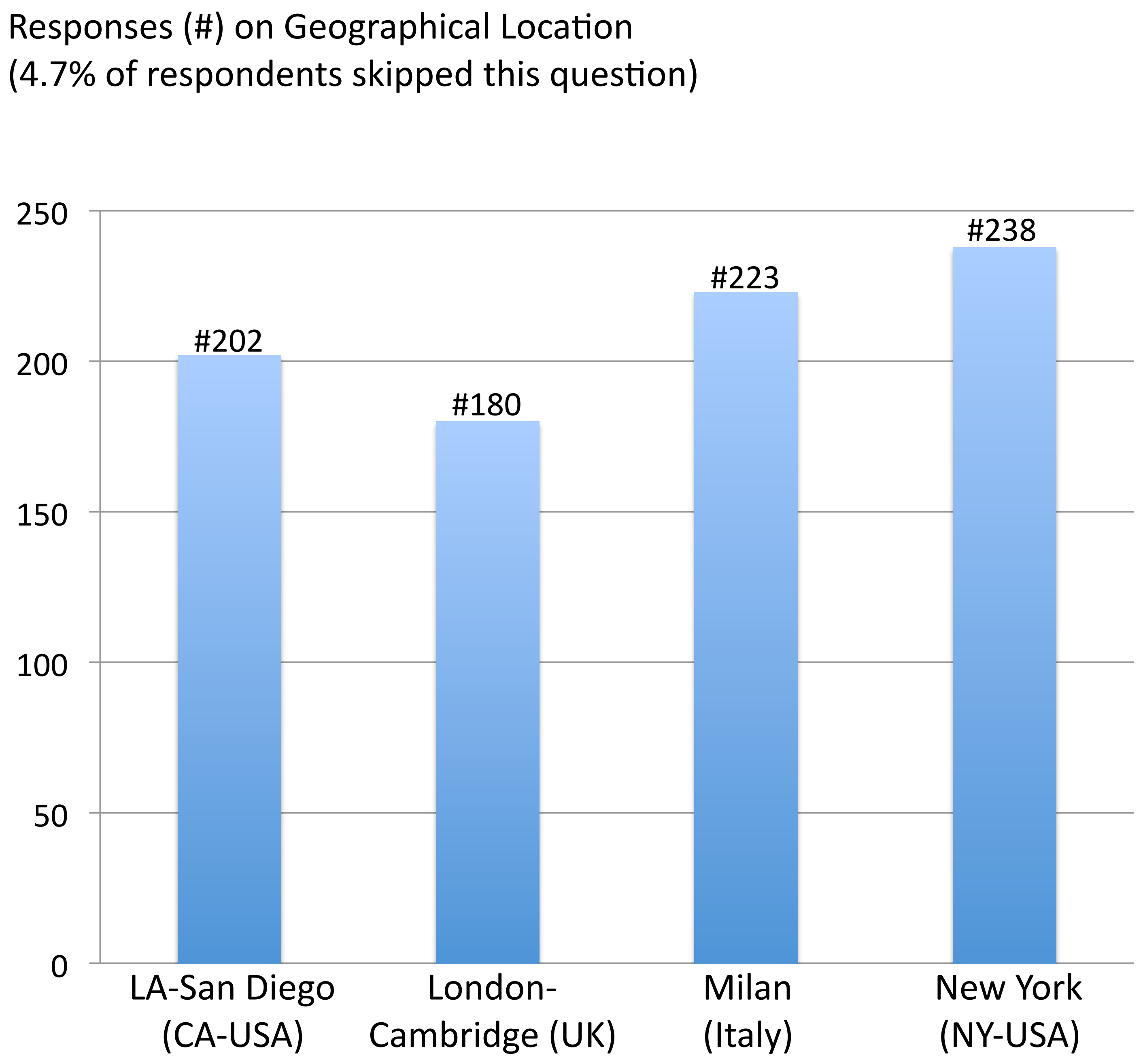
Responses (#) on Geographical Location
(4.7% of respondents skipped this question)
250
200
150
100
50
0
#202
#180
#223
#238
LA-San Diego (CA-USA)
London-Cambridge (UK)
Milan (Italy)
New York (NY-USA)

Fig. S15

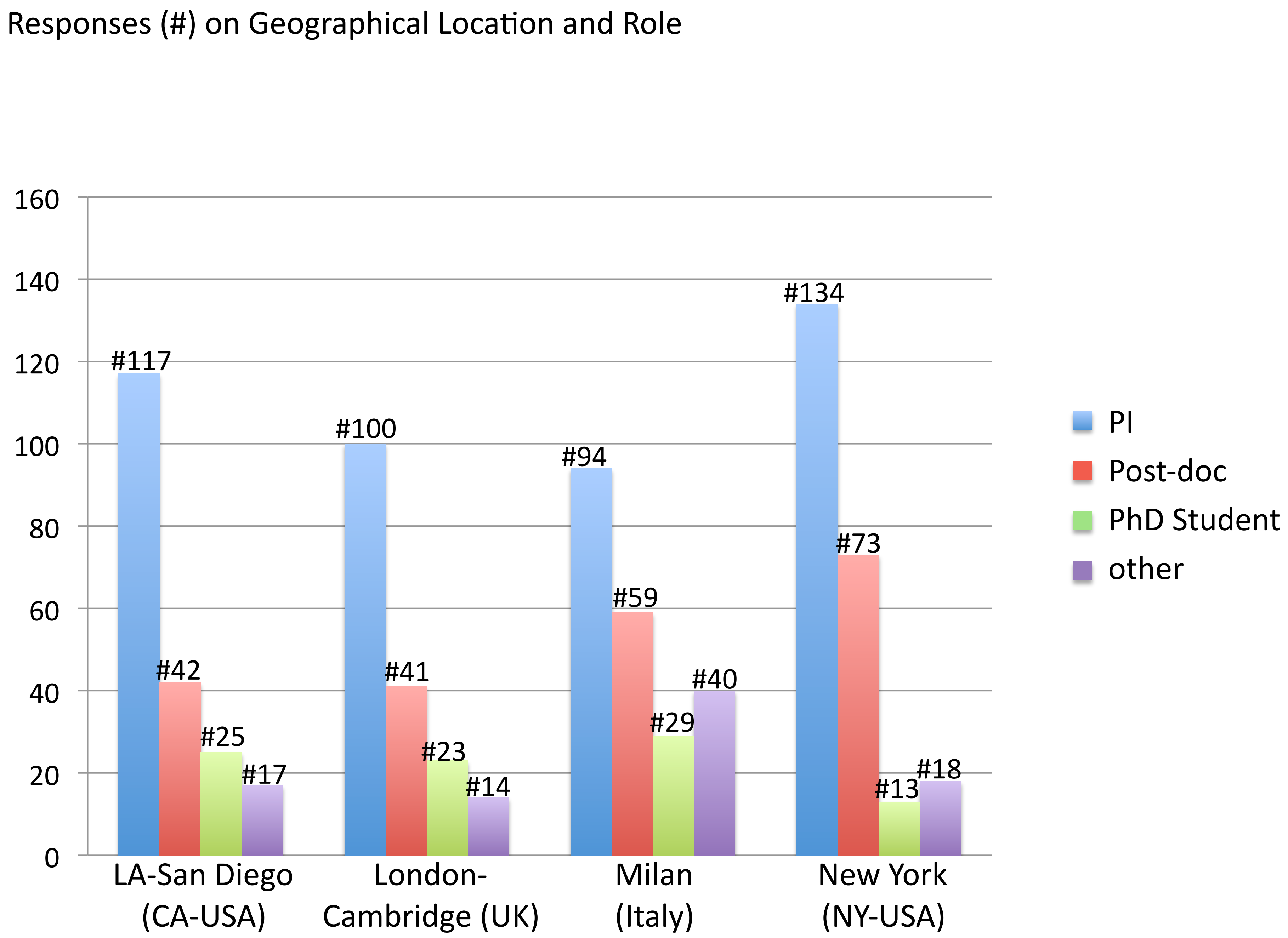
Responses (#) on Geographical Location and Role
160
140
120
100
80
60
40
20
0
#117
#42
#25
#17
#100
#41
#23
#14
#94
#59
#29
#40
#134
#73
#13
#18
LA-San Diego (CA-USA)
London-Cambridge (UK)
Milan (Italy)
New York (NY-USA)
PI
Post-doc
PhD Student
other

Fig. S16a

Responses (#) on Geographical Location and Role for Females

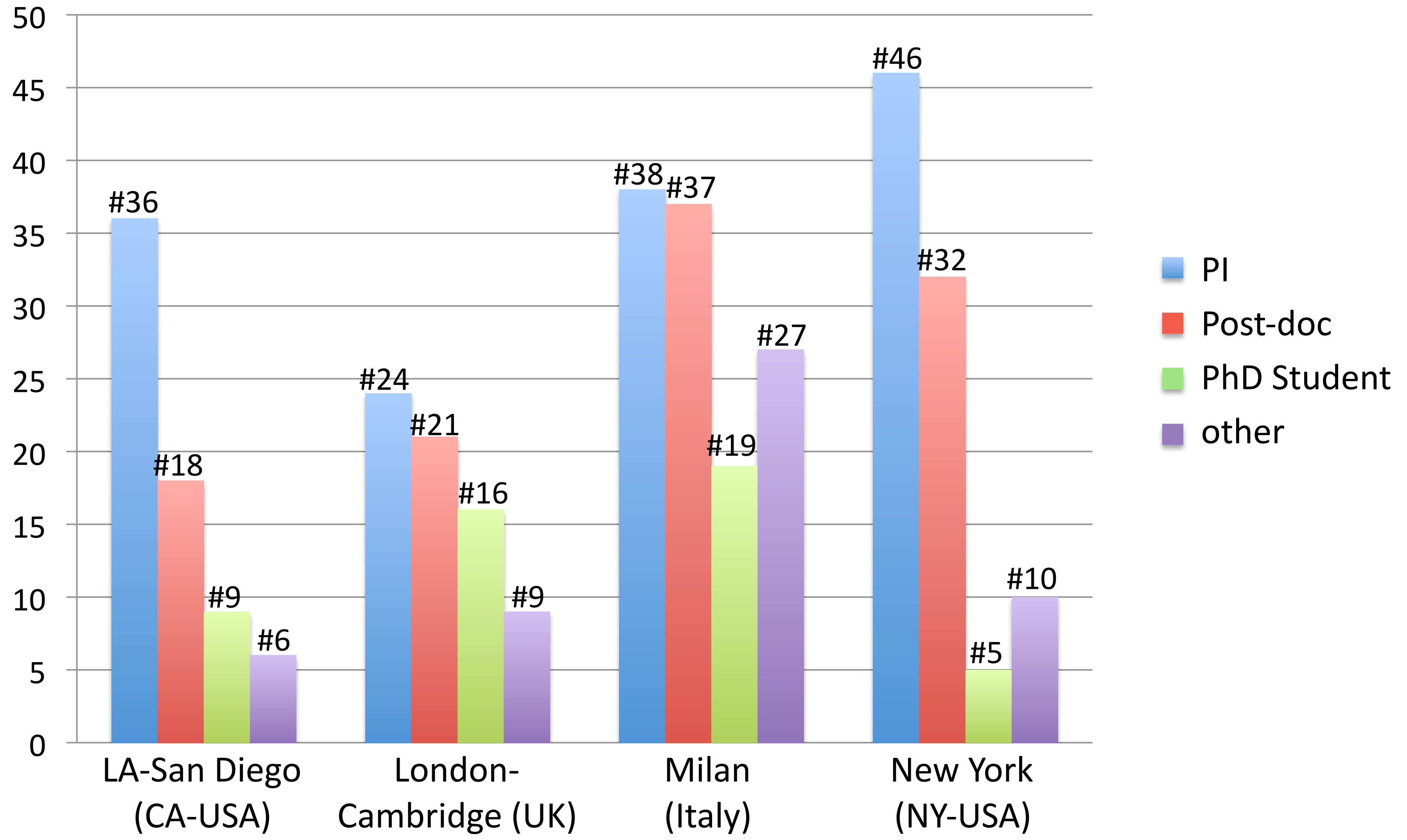

Fig. S16b

Responses (#) on Geographical Location and Role for Males

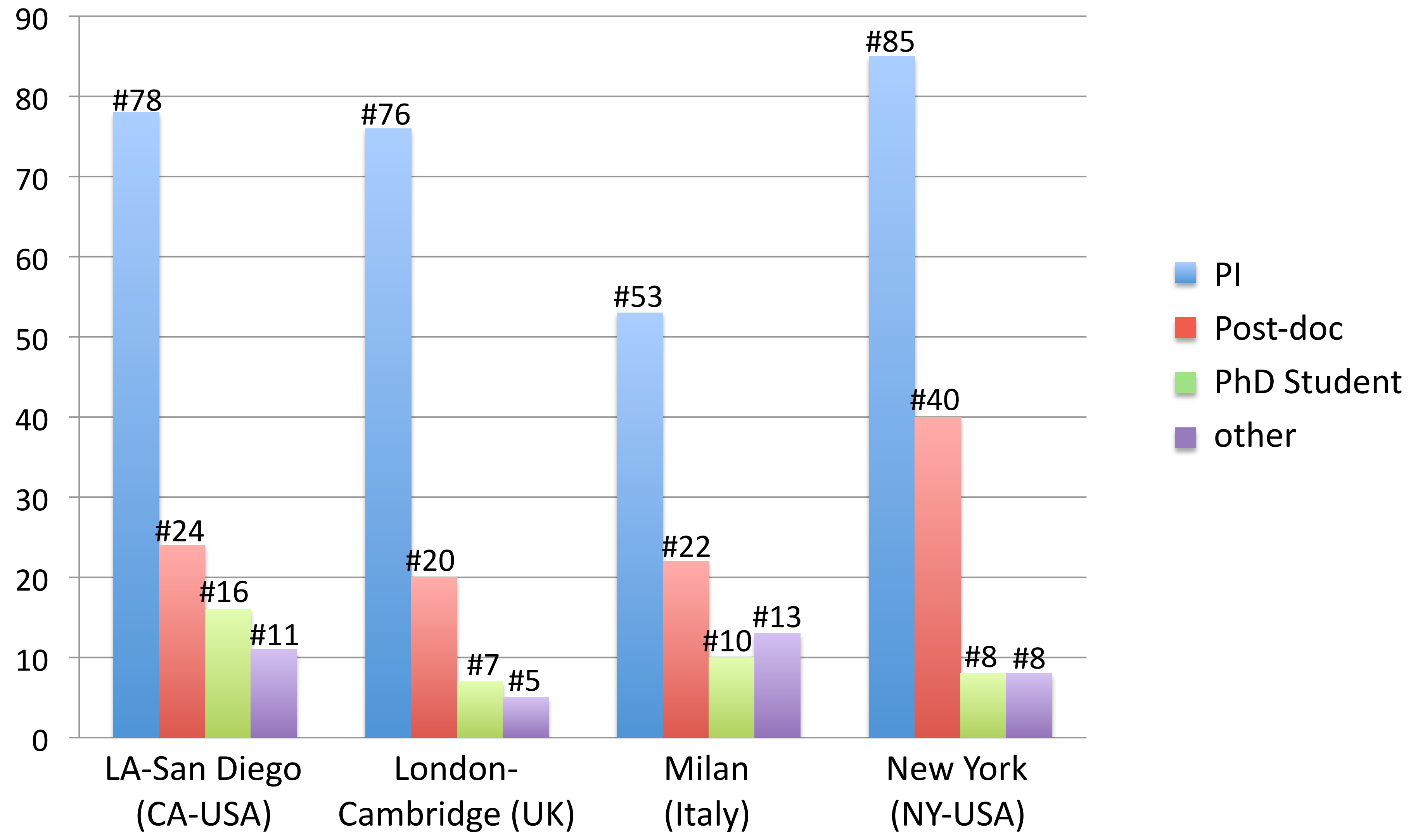

Fig. S16c

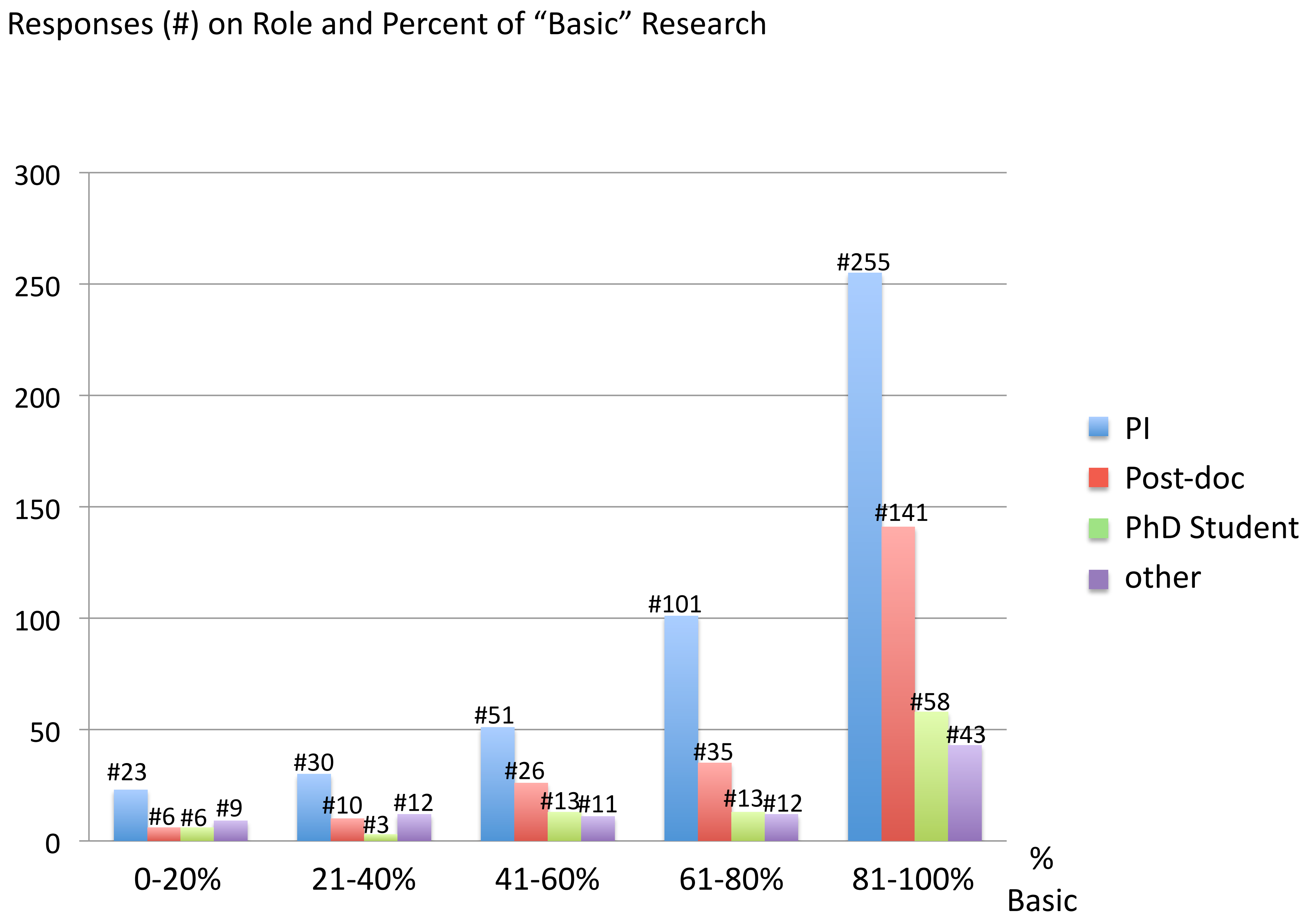

Responses (#) on Role and Percent of “Basic” Research
300
250
200
150
100
50
0
#23
#6
#6
#9
#30
#10
#3
#12
#51
#26
#13
#11
#101
#35
#13
#12
#255
#141
#58
#43
0-20%
21-40%
41-60%
61-80%
81-100%
% Basic
PI
Post-doc
PhD Student
other

Fig. S17

Responses (#) on Gender and Percent of "Basic" Research
For Principal Investigators

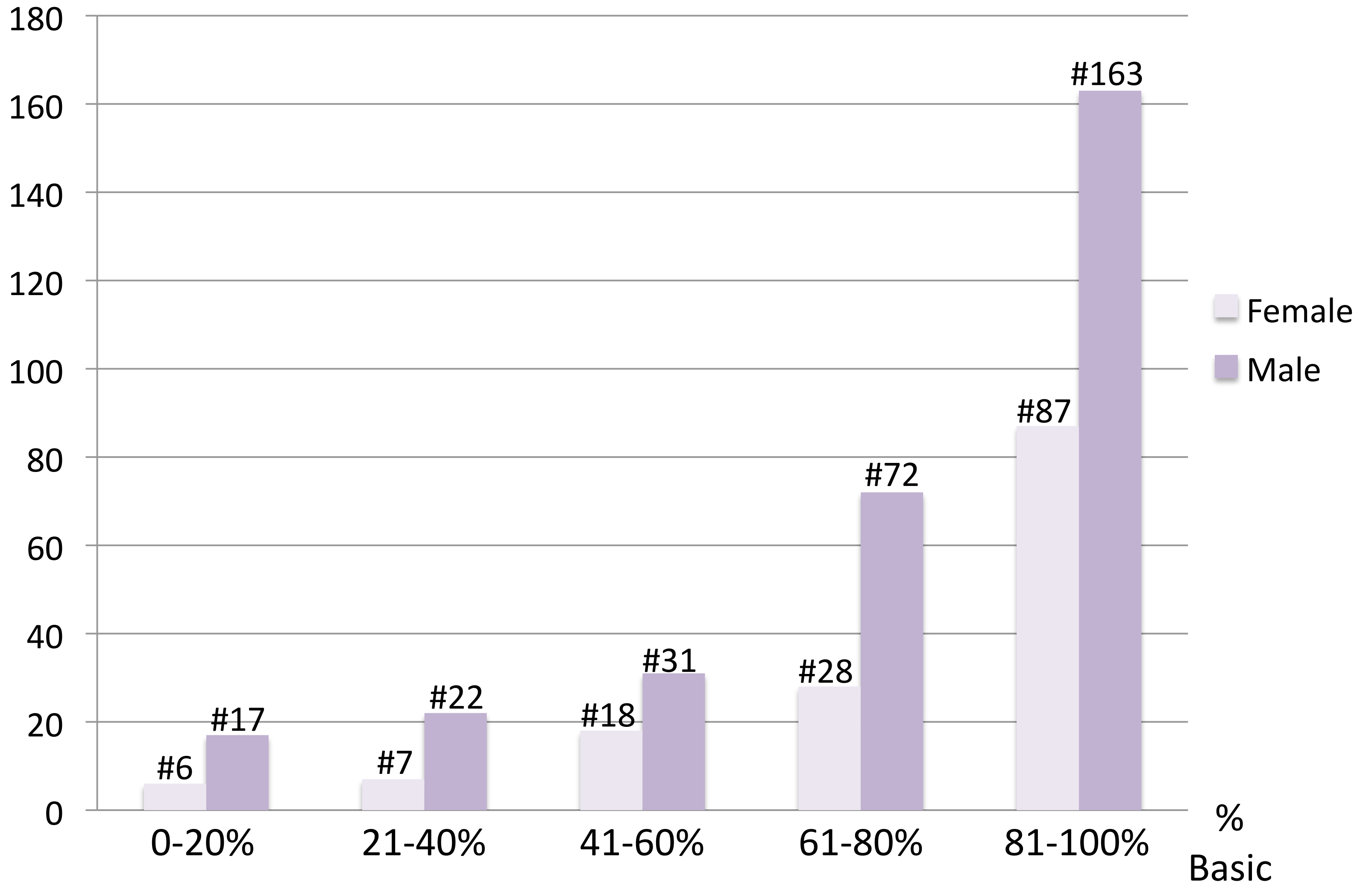

Fig. S18

Percentages of Percent of “Basic” Research per Females/Males Principal Investigators

	**0-20%**	**21-40%**	**41-60%**	**61-80%**	**81-100%**	Total
**Female**	4.1	4.8	12.3	19.2	59.6	100
**Male**	5.6	7.2	10.2	23.6	53.4	100
Total	5.1	6.4	10.9	22.2	55.4	100

Fig. S18
