# Supplementary material for: Increasing both the public health potential of basic research and the scientist satisfaction. An international survey of bio-scientists
Source: F1000Res. 2016 Jun 1;5:56. Originally published 2016 Jan 12. [Version 2] doi: 10.12688/f1000research.7683.2 (PMC4909114; doi:10.12688/f1000research.7683.2)
Supplement: Supplementary file 5 [file f1000research-5-9472-s0004.tgz › c2fd43b3-eeb7-4c9a-84a6-063bd7ed5cfd.pdf]

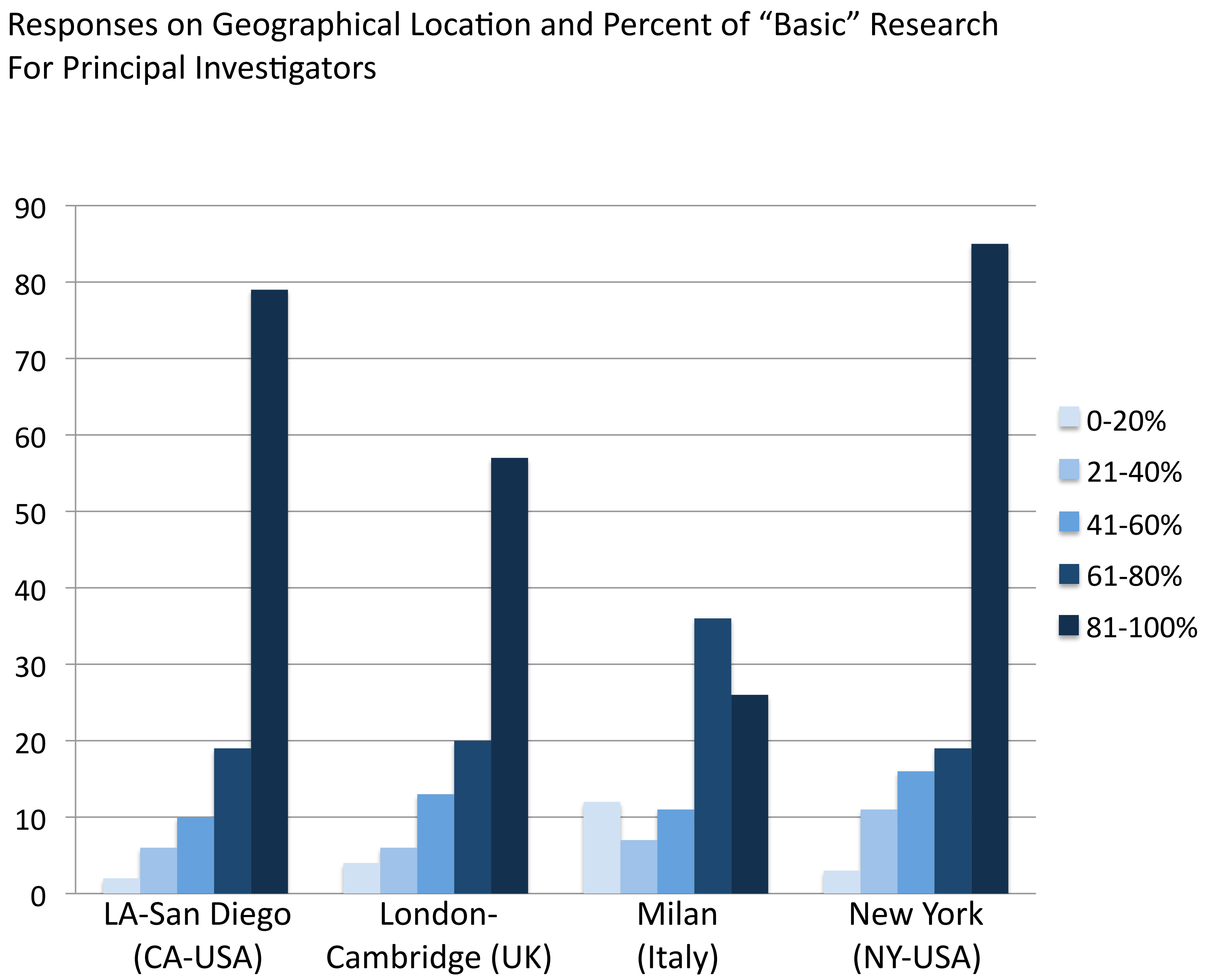
Responses on Geographical Location and Percent of “Basic” Research
For Principal Investigators
90
80
70
60
50
40
30
20
10
0
LA-San Diego (CA-USA)
London-Cambridge (UK)
Milan (Italy)
New York (NY-USA)
0-20%
21-40%
41-60%
61-80%
81-100%

Fig. S19

Percentages of Percent of “Basic” Research per Geographical Location
For Total/Females/Males Principal Investigators

Total

	0-20%	21-40%	41-60%	61-80%	81-100%	Total
**LA-SD (CA-USA)**	1.7	5.2	8.6	16.4	68.1	100
**London-Camb. (UK)**	4	6	13	20	57	100
**Milan (Italy)**	13	7.6	12	39.1	28.3	100
**NYC (NY-USA)**	2.2	8.2	11.9	14.2	63.4	100
Total	4.8	6.8	11.3	21.3	55.9	100

Females

	0-20%	21-40%	41-60%	61-80%	81-100%	Total
**LA-SD (CA-USA)**	0	0	14.3	5.7	80	100
**London-Camb. (UK)**	0	4.2	12.5	20.8	62.5	100
**Milan (Italy)**	10.8	8.1	13.5	32.4	35.1	100
**NYC (NY-USA)**	4.3	6.5	10.9	15.2	63	100
Total	4.2	4.9	12.7	18.3	59.9	100

Males

	0-20%	21-40%	41-60%	61-80%	81-100%	Total
**LA-SD (CA-USA)**	2.6	7.7	6.4	21.8	61.5	100
**London-Camb. (UK)**	5.3	6.6	13.2	19.7	55.3	100
**Milan (Italy)**	15.4	5.8	11.5	44.2	23.1	100
**NYC (NY-USA)**	1.2	9.4	10.6	14.1	64.7	100
Total	5.2	7.6	10.3	23	54	100

Fig. S19

Your personal motivations as a scientist are from:
(a) Pure advancement of knowledge, regardless of future applicability
Principal Investigators ordered by geographical location

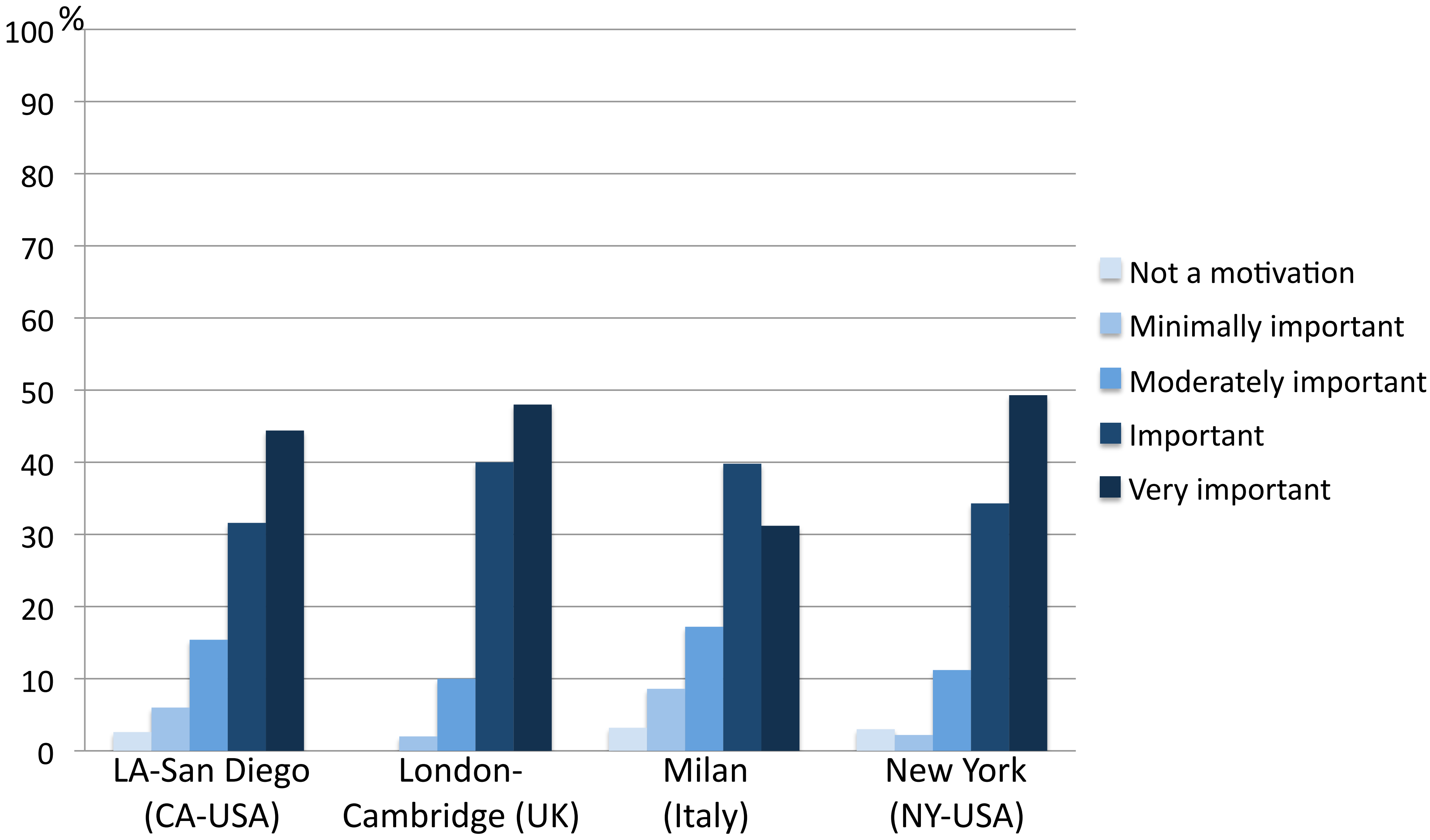

Fig. S20a

	Not a motivation	Minimally important	Moderately important	Important	Very important	Tota l	
**LA-SD (CA-USA)**	2.6	6	15.4	31.6	44.4	100	
**London-Camb. (UK)**	0	2	10	40	48	100	
**Milan (Italy)**	3.2	8.6	17.2	39.8	31.2	100	0-100% Basic Research
**NYC (NY-USA)**	3	2.2	11.2	34.3	49.3	100	
Total	2.3	4.5	13.3	36	43.9	100	

	Not a motivation	Minimally important	Moderately important	Important	Very important	Tot al	
**LA-SD (CA-USA)**	3.8	1.3	8.9	31.6	54.4	100	
**London-Camb. (UK)**	0	0	5.3	36.8	57.9	100	
**Milan (Italy)**	0	0	19.2	34.6	46.2	100	81-100% Basic Research
**NYC (NY-USA)**	2.4	1.2	8.2	27.1	61.2	100	
Total	2	0.8	8.9	31.6	56.7	100	

Fig. S20a

Your personal motivations as a scientist are from:
(b) Health benefit to society (not necessarily in the near future)
Principal Investigators ordered by geographical location

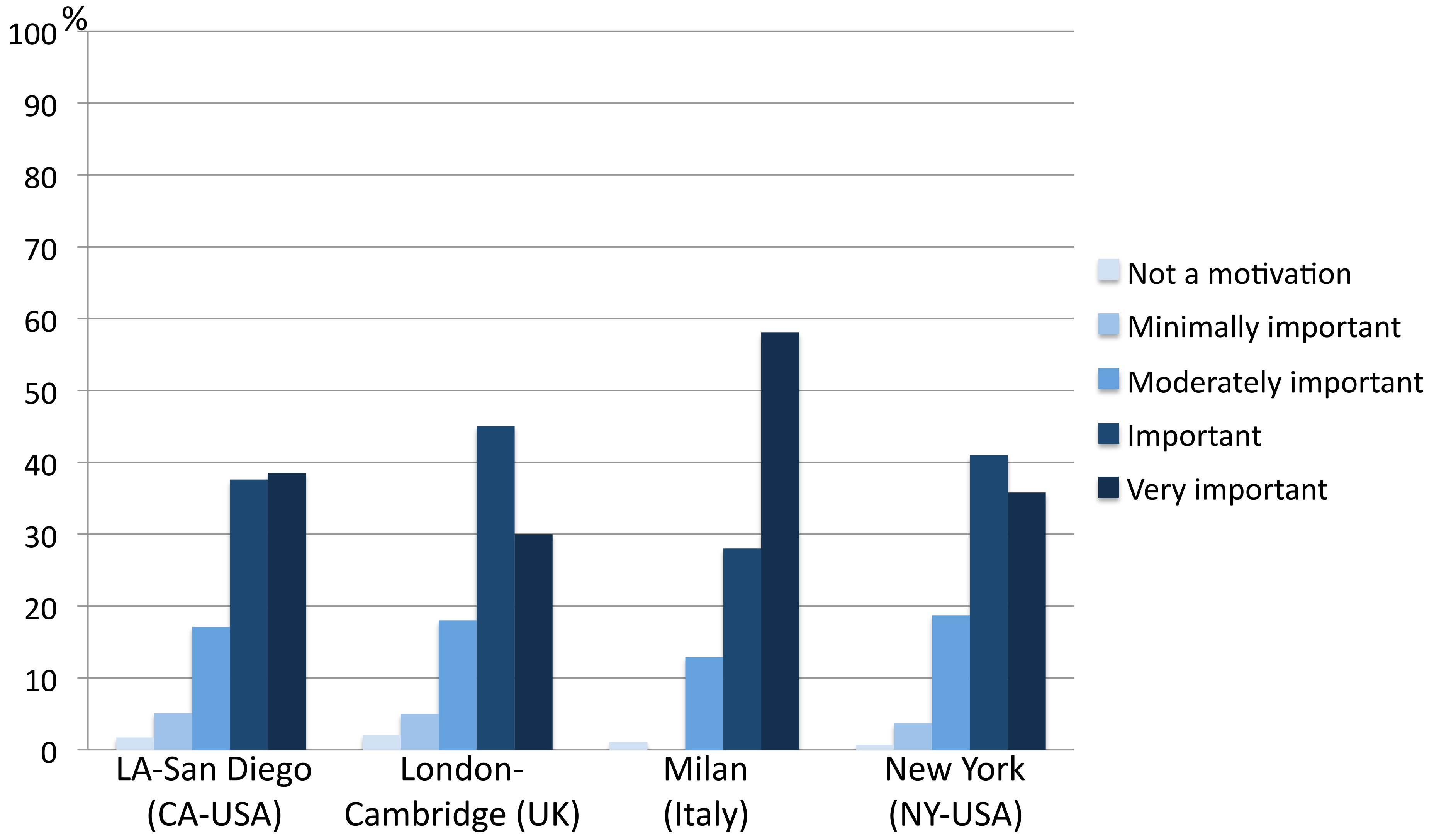

Fig. S20b

	**Not a motivation**	**Minimally important**	**Moderately important**	**Important**	**Very important**	Tot al	
**LA-SD (CA-USA)**	1.7	5.1	17.1	37.6	38.5	100	
**London-Camb. (UK)**	2	5	18	45	30	100	
**Milan (Italy)**	1.1	0	12.9	28	58.1	100	0-100% Basic Research
**NYC (NY-USA)**	0.7	3.7	18.7	41	35.8	100	
Total	1.4	3.6	16.9	38.3	39.9	100	

	**Not a motivation**	**Minimally important**	**Moderately important**	**Important**	**Very important**	Tot al	
**LA-SD (CA-USA)**	2.5	7.6	20.3	38	31.6	100	
**London-Camb. (UK)**	3.5	8.8	19.3	49.1	19.3	100	
**Milan (Italy)**	3.8	0	11.5	26.9	57.7	100	81-100% Basic Research
**NYC (NY-USA)**	1.2	5.9	23.5	42.4	27.1	100	
Total	2.4	6.5	20.2	40.9	30	100	

Fig. S20b

Your personal motivations as a scientist are from:
(c) Gain of prestige
Principal Investigators ordered by geographical location

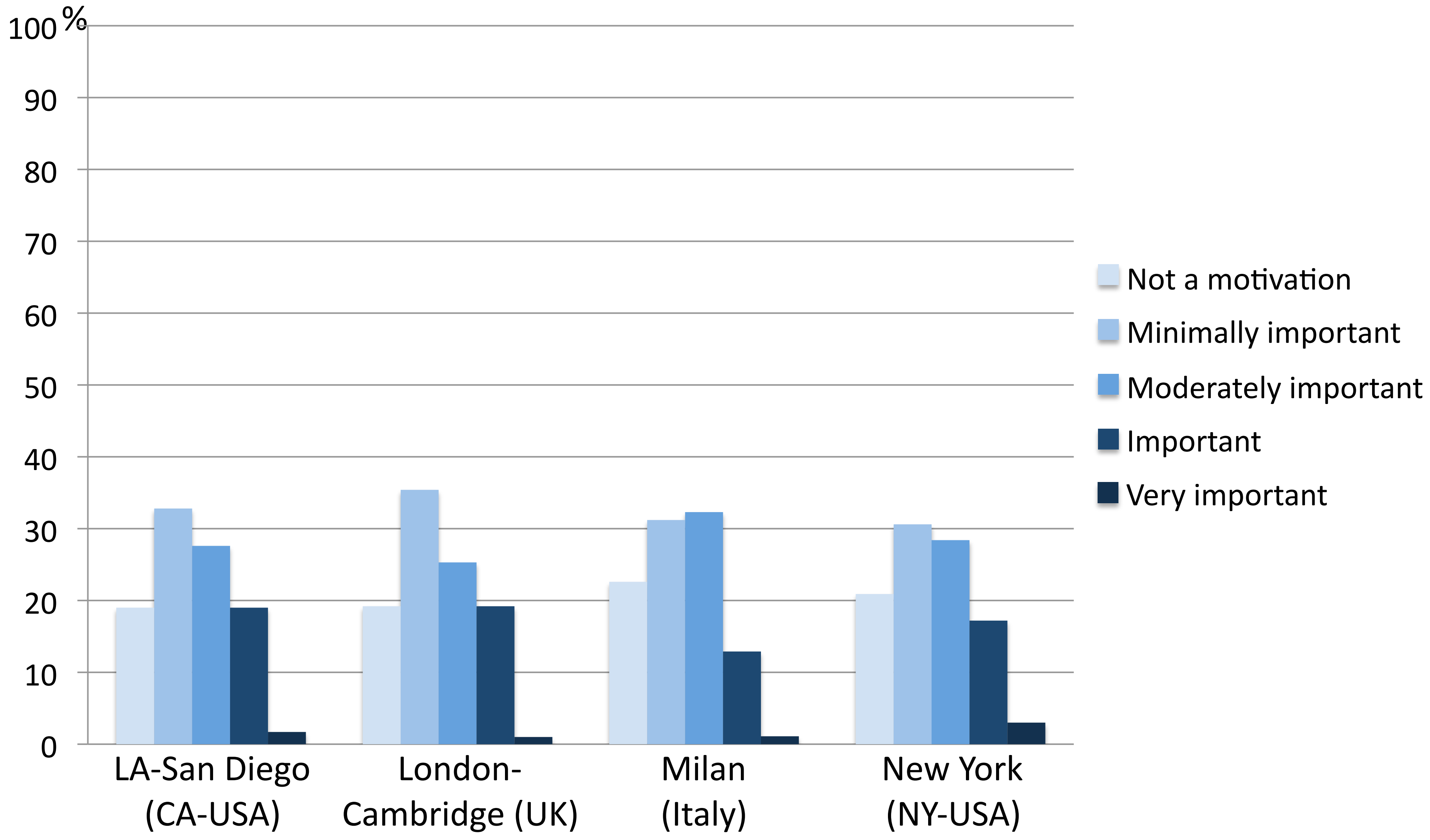

Fig. S20c

	Not a motivation	Minimally important	Moderately important	Important	Very important	Total	
**LA-SD (CA-USA)**	19	32.8	27.6	19	1.7	100	
**London-Camb. (UK)**	19.2	35.4	25.3	19.2	1	100	0-100% Basic Research
**Milan (Italy)**	22.6	31.2	32.3	12.9	1.1	100	
**NYC (NY-USA)**	20.9	30.6	28.4	17.2	3	100	
Total	20.4	32.4	28.3	17.2	1.8	100	

	Not a motivation	Minimally important	Moderately important	Important	Very important	Total	
**LA-SD (CA-USA)**	19.2	29.5	29.5	19.2	2.6	100	
**London-Camb. (UK)**	22.8	29.8	26.3	21.1	0	100	81-100% Basic Research
**Milan (Italy)**	19.2	38.5	30.8	11.5	0	100	
**NYC (NY-USA)**	28.2	29.4	20	22.4	0	100	
Total	23.2	30.5	25.6	19.9	0.8	100	

Fig. S20c

Your personal motivations as a scientist are from:
(d) Gain of money (for personal purposes)
Principal Investigators ordered by geographical location

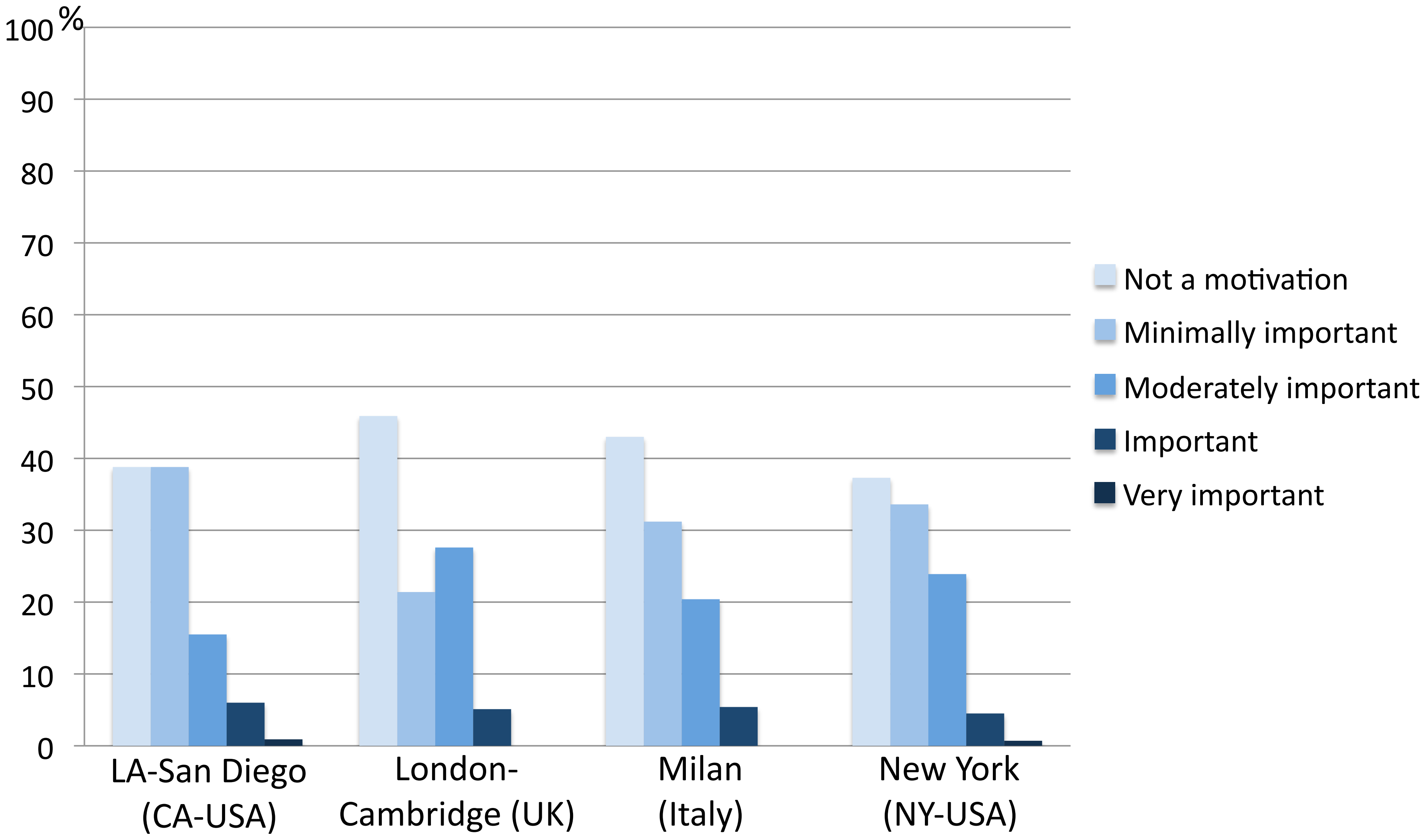

Fig. S20d

	Not a motivation	Minimally important	Moderately important	Important	Very important	Tota l
**LA-SD (CA-USA)**	38.8	38.8	15.5	6	0.9	100
**London-Camb. (UK)**	45.9	21.4	27.6	5.1	0	100
**Milan (Italy)**	43	31.2	20.4	5.4	0	100
**NYC (NY-USA)**	37.3	33.6	23.9	4.5	0.7	100
Total	40.8	31.7	21.8	5.2	0.5	100

0-100% Basic Research

	Not a motivation	Minimally important	Moderately important	Important	Very important	Total
**LA-SD (CA-USA)**	38.5	39.7	16.7	3.8	1.3	100
**London-Camb. (UK)**	45.6	24.6	28.1	1.8	0	100
**Milan (Italy)**	46.2	34.6	15.4	3.8	0	100
**NYC (NY-USA)**	43.5	32.9	20	2.4	1.2	100
Total	42.7	33.3	20.3	2.8	0.8	100

81-100% Basic Research

Fig. S20d

Your personal motivations as a scientist are from:
(e) Satisfaction of your curiosity
Principal Investigators ordered by geographical location

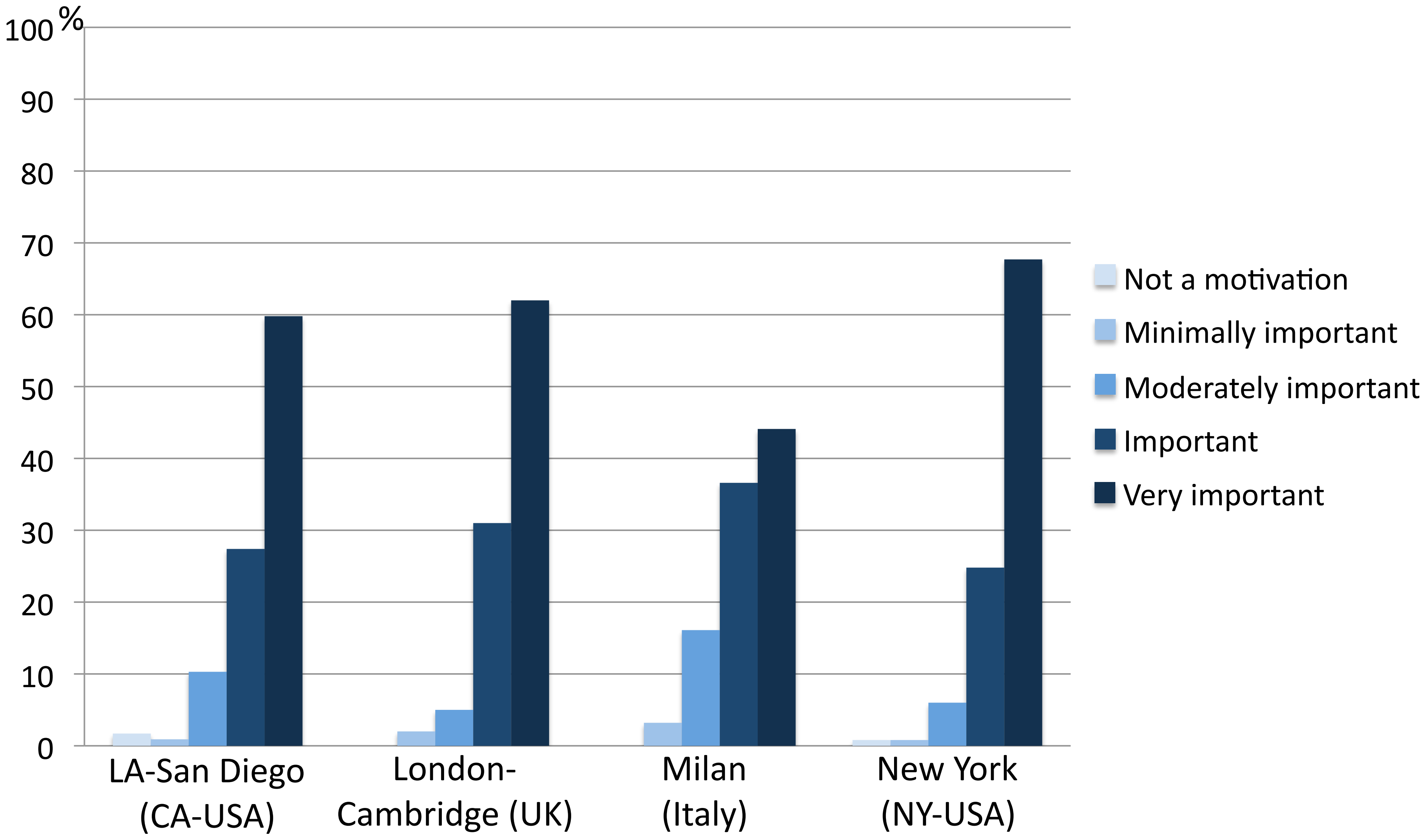

Fig. S20e

	Not a motivation	Minimally important	Moderately important	Important	Very important	Total	
**LA-SD (CA-USA)**	1.7	0.9	10.3	27.4	59.8	100	
**London-Camb. (UK)**	0	2	5	31	62	100	0-100% Basic Research
**Milan (Italy)**	0	3.2	16.1	36.6	44.1	100	
**NYC (NY-USA)**	0.8	0.8	6	24.8	67.7	100	
Total	0.7	1.6	9	29.3	59.4	100	

	Not a motivation	Minimally important	Moderately important	Important	Very important	Total	
**LA-SD (CA-USA)**	2.5	0	7.6	26.6	63.3	100	
**London-Camb. (UK)**	0	1.8	0	29.8	68.4	100	81-100% Basic Research
**Milan (Italy)**	0	7.7	11.5	26.9	53.8	100	
**NYC (NY-USA)**	1.2	0	5.9	21.2	71.8	100	
Total	1.2	1.2	5.7	25.5	66.4	100	

Fig. S20e

Your personal motivations as a scientist are from:
(f) Satisfaction from solving puzzling problems
Principal Investigators ordered by geographical location

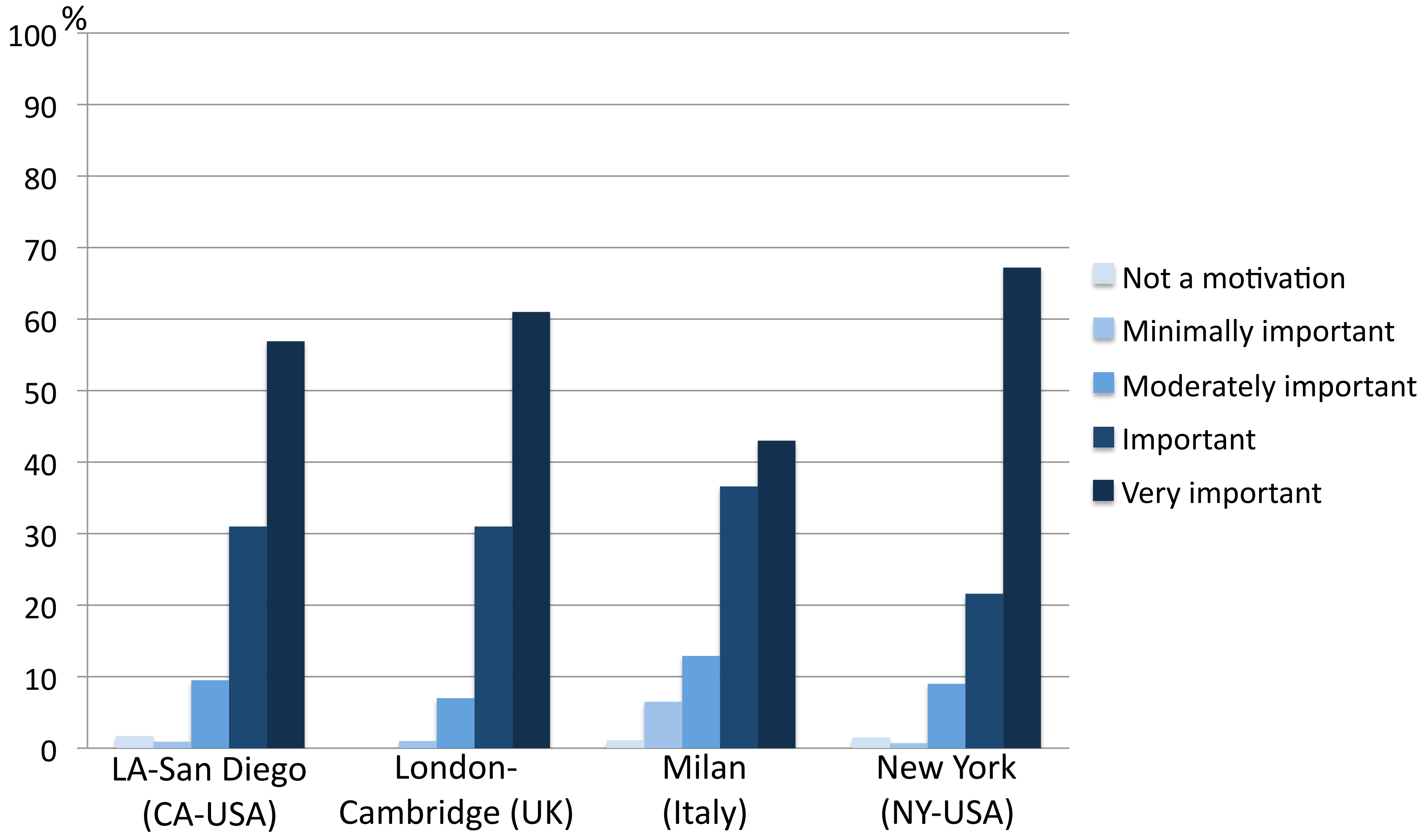

Fig. S20f

	Not a motivation	Minimally important	Moderately important	Important	Very important	Total	
**LA-SD (CA-USA)**	1.7	0.9	9.5	31	56.9	100	0-100% Basic Research
**London-Camb. (UK)**	0	1	7	31	61	100	
**Milan (Italy)**	1.1	6.5	12.9	36.6	43	100	
**NYC (NY-USA)**	1.5	0.7	9	21.6	67.2	100	
Total	1.1	2	9.5	29.3	58	100	

	Not a motivation	Minimally important	Moderately important	Important	Very important	Total	
**LA-SD (CA-USA)**	1.3	0	9	28.2	61.5	100	81-100% Basic Research
**London-Camb. (UK)**	0	1.8	1.8	35.1	61.4	100	
**Milan (Italy)**	0	0	26.9	19.2	53.8	100	
**NYC (NY-USA)**	1.2	0	8.2	21.2	69.4	100	
Total	0.8	0.4	8.9	26.4	63.4	100	

Fig. S20f

Basic scientists can ponder about the future indirect practical benefits of their research without losing their "basic status”
Principal Investigators ordered by geographical location

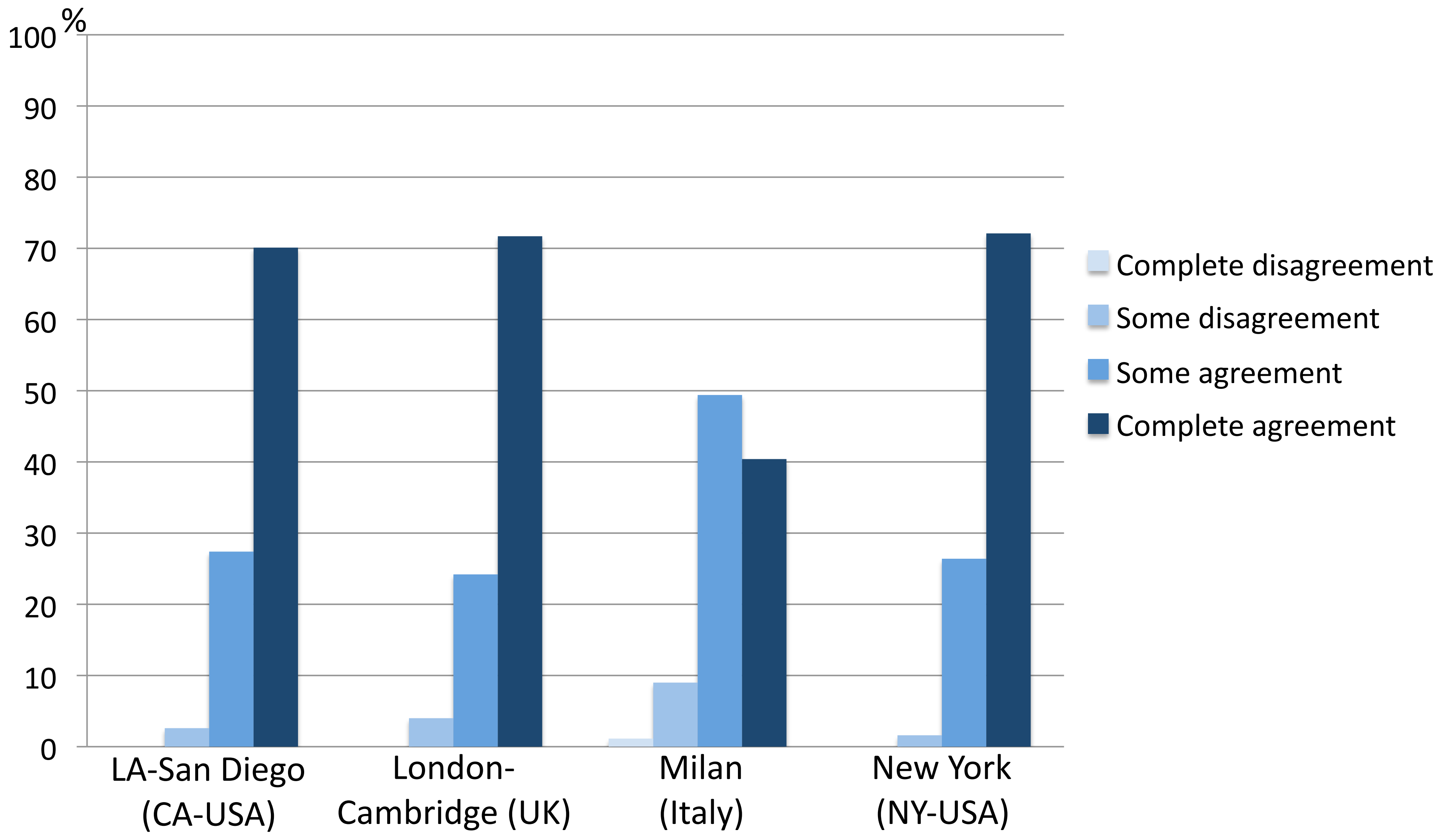

Fig. S21

	Complete disagreement	Some disagreement	Some agreement	Complete agreement	Total	
**LA-SD (CA-USA)**	0	2.6	27.4	70.1	100	
**London-Camb. (UK)**	0	4	24.2	71.7	100	0-100% Basic Research
**Milan (Italy)**	1.1	9	49.4	40.4	100	
**NYC (NY-USA)**	0	1.6	26.4	72.1	100	
Total	0.2	3.9	30.9	65	100	

	Complete disagreement	Some disagreement	Some agreement	Complete agreement	Total	
**LA-SD (CA-USA)**	0	3.8	25.3	70.9	100	
**London-Camb. (UK)**	0	1.8	21.4	76.8	100	81-100% Basic Research
**Milan (Italy)**	0	8	60	32	100	
**NYC (NY-USA)**	0	1.2	19.8	79	100	
Total	0	2.9	26.1	71	100	

Fig. S21

What should the most important goal of publicly funded basic BIOLOGICAL (not biomedical) research be?
Principal Investigators ordered by geographical location

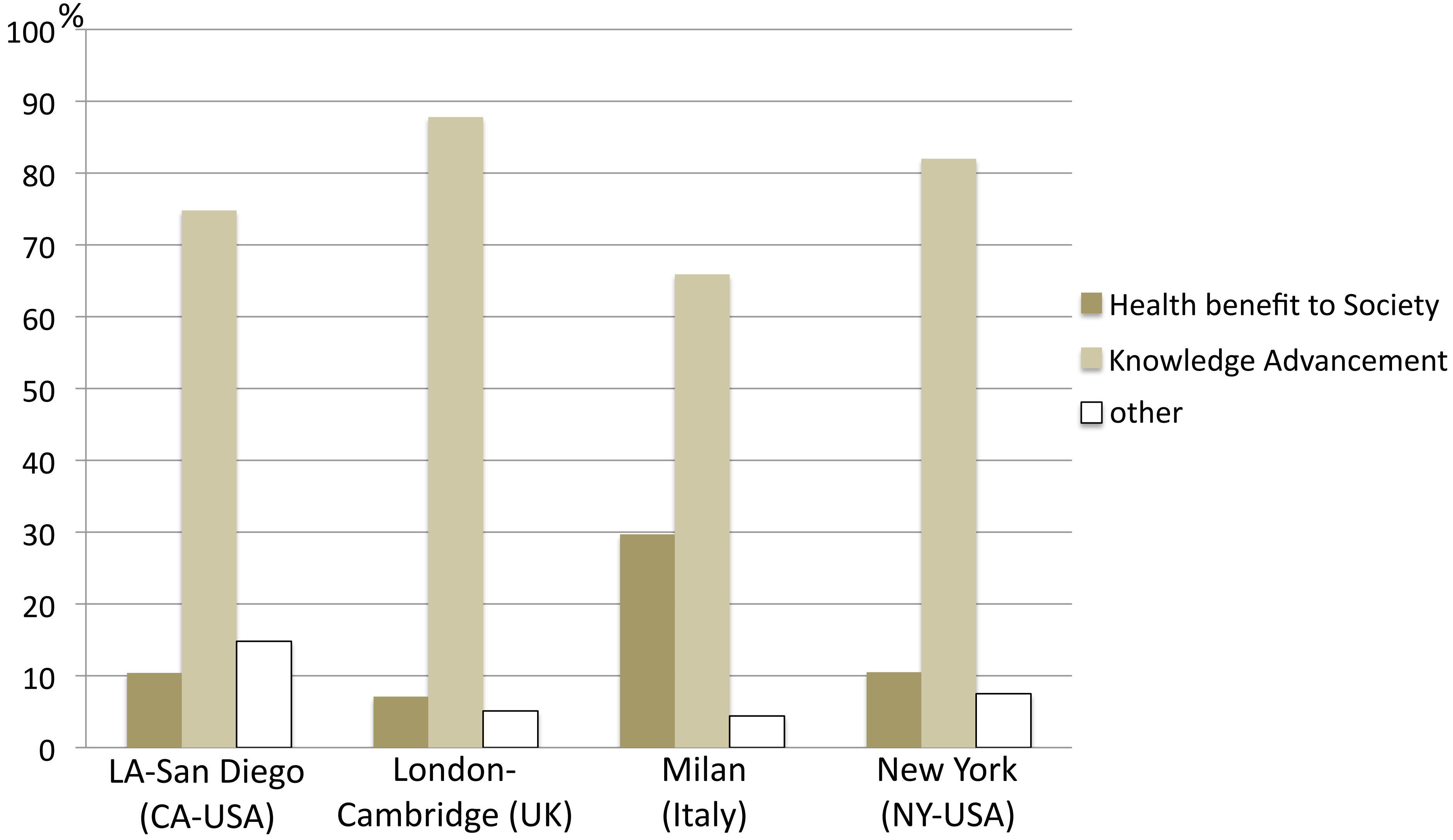

Fig. S22a

	Health benefit to society (not necessarily in the near future)	Pure advancement of knowledge, regardless of future applicability	Other	Total	
**LA-SD (CA-USA)**	10.4	74.8	14.8	100	
**London-Camb. (UK)**	7.1	87.8	5.1	100	0-100%
**Milan (Italy)**	29.7	65.9	4.4	100	Basic Research
**NYC (NY-USA)**	10.5	82	7.5	100	
Total	13.7	78	8.2	100	

	Health benefit to society (not necessarily in the near future)	Pure advancement of knowledge, regardless of future applicability	Other	Total	
**LA-SD (CA-USA)**	10.3	74.4	15.4	100	
**London-Camb. (UK)**	3.6	92.9	3.6	100	81-100%
**Milan (Italy)**	19.2	73.1	7.7	100	Basic Research
**NYC (NY-USA)**	8.2	83.5	8.2	100	
Total	9	81.6	9.4	100	

Fig. S22a

What should the most important goal of publicly funded basic BIOMEDICAL research be?
Principal Investigators ordered by geographical location

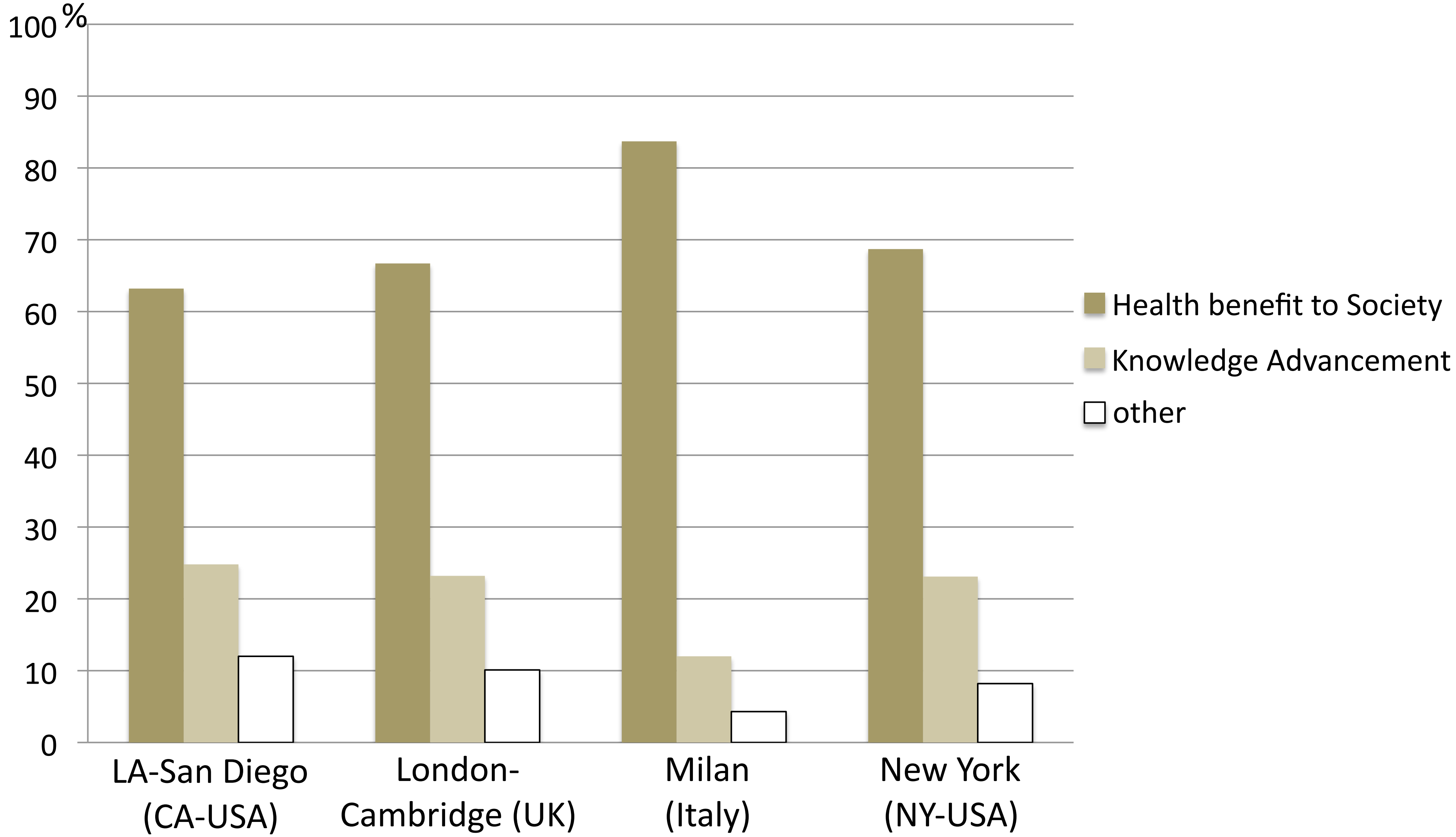

Fig. S22b

	Health benefit to society (not necessarily in the near future)	Pure advancement of knowledge, regardless of future applicability	Other	Total	
**LA-SD (CA-USA)**	63.2	24.8	12	100	
**London-Camb. (UK)**	66.7	23.2	10.1	100	0-100% Basic Research
**Milan (Italy)**	83.7	12	4.3	100	
**NYC (NY-USA)**	68.7	23.1	8.2	100	
Total	69.9	21.3	8.8	100	

	Health benefit to society (not necessarily in the near future)	Pure advancement of knowledge, regardless of future applicability	Other	Total	
**LA-SD (CA-USA)**	55.7	30.4	13.9	100	
**London-Camb. (UK)**	55.4	32.1	12.5	100	81-100% Basic Research
**Milan (Italy)**	76.9	15.4	7.7	100	
**NYC (NY-USA)**	62.4	30.6	7.1	100	
Total	60.2	29.3	10.6	100	

Fig. S22b

Although it is difficult to assess the potential future health benefits to society from basic biological/biomedical research as described in written proposals, some degree of estimation is always possible.
Principal Investigators ordered by geographical location

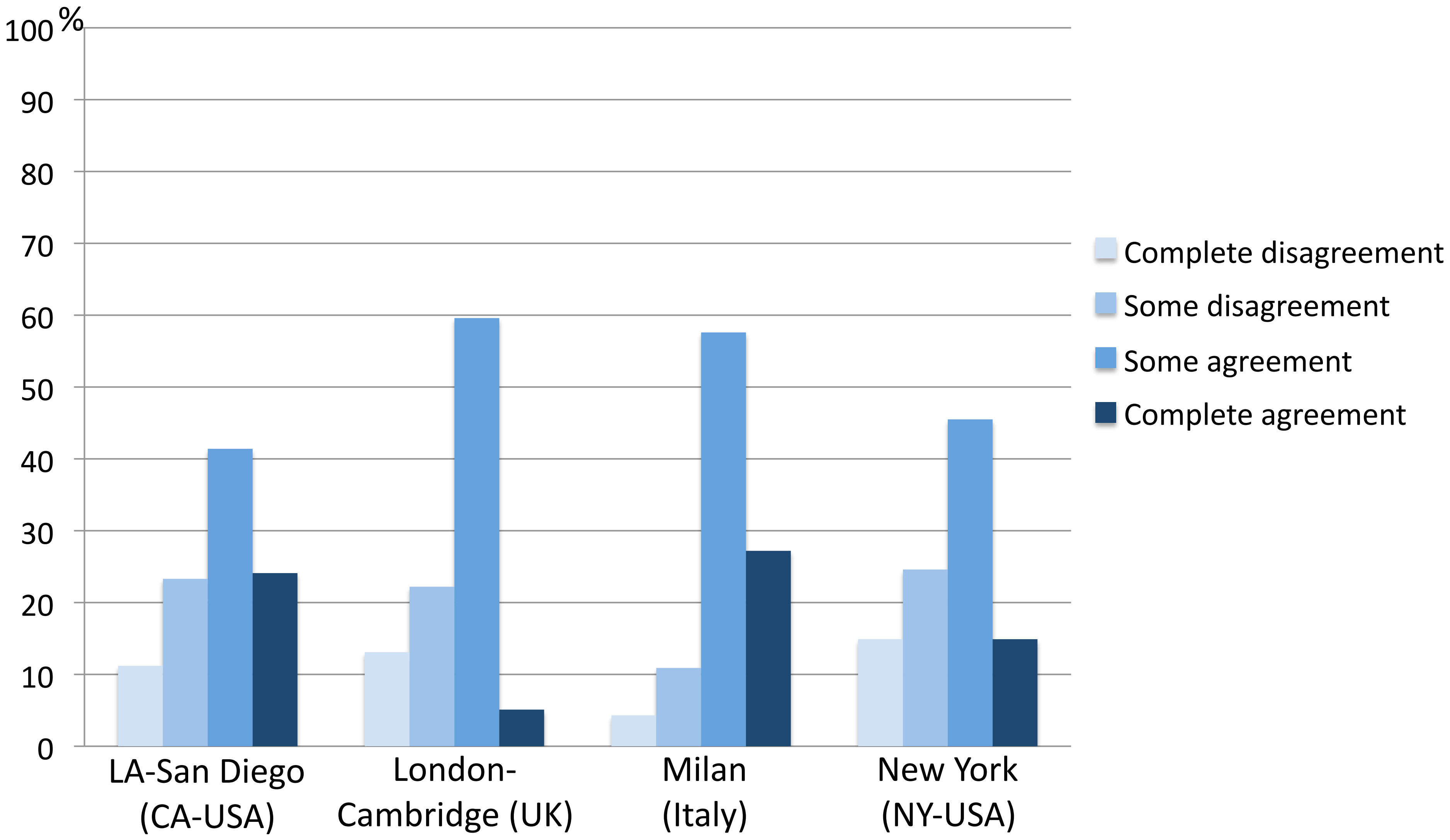

Fig. S23

	Complete disagreement	Some disagreement	Some agreement	Complete agreement	Total	
**LA-SD (CA-USA)**	11.2	23.3	41.4	24.1	100	
**London-Camb. (UK)**	13.1	22.2	59.6	5.1	100	0-100% Basic Research
**Milan (Italy)**	4.3	10.9	57.6	27.2	100	
**NYC (NY-USA)**	14.9	24.6	45.5	14.9	100	
Total	11.3	20.9	50.1	17.7	100	

	Complete disagreement	Some disagreement	Some agreement	Complete agreement	Total	
**LA-SD (CA-USA)**	15.2	29.1	35.4	20.3	100	
**London-Camb. (UK)**	19.3	24.6	54.4	1.8	100	81-100% Basic Research
**Milan (Italy)**	7.7	7.7	65.4	19.2	100	
**NYC (NY-USA)**	14.1	23.5	49.4	12.9	100	
Total	15	23.9	47.8	13.4	100	

Fig. S23

Written proposals about basic biological/biomedical research generally contain a section discussing potential future health benefits. These sections increase the likelihood that a project benefits future public health. Principal Investigators ordered by geographical location

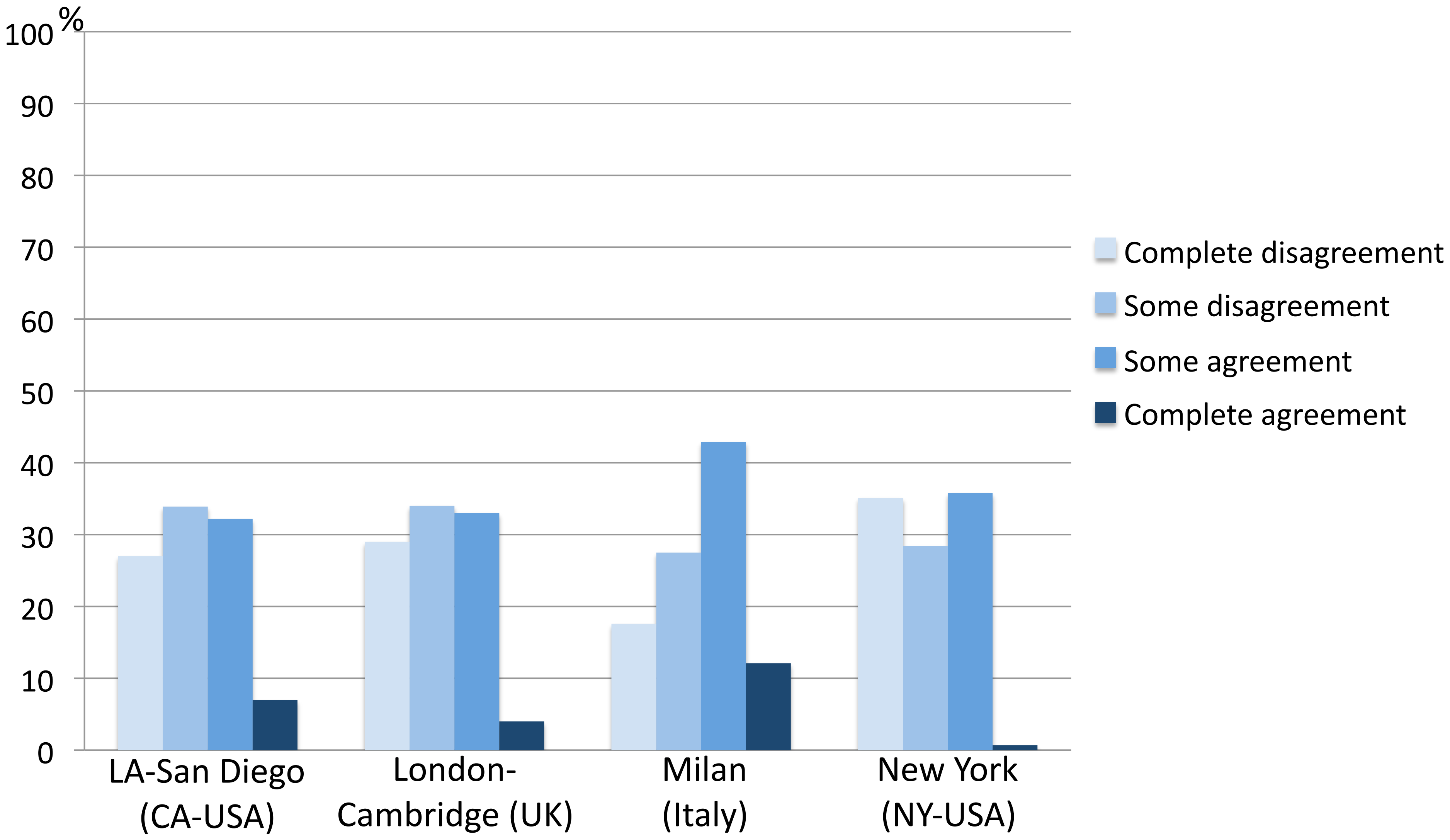

Fig. S24

	Complete disagreement	Some disagreement	Some agreement	Complete agreement	Total	
**LA-SD (CA-USA)**	27	33.9	32.2	7	100	
**London-Camb. (UK)**	29	34	33	4	100	0-100%
**Milan (Italy)**	17.6	27.5	42.9	12.1	100	Basic Research
**NYC (NY-USA)**	35.1	28.4	35.8	0.7	100	
Total	28	30.9	35.7	5.5	100	

	Complete disagreement	Some disagreement	Some agreement	Complete agreement	Total	
**LA-SD (CA-USA)**	32.9	34.2	25.3	7.6	100	
**London-Camb. (UK)**	31.6	38.6	28.1	1.8	100	81-100%
**Milan (Italy)**	23.1	34.6	34.6	7.7	100	Basic Research
**NYC (NY-USA)**	37.6	32.9	29.4	0	100	
Total	33.2	34.8	28.3	3.6	100	

Fig. S24

Writing the sections discussing potential future health benefits takes too much time.
Principal Investigators ordered by geographical location

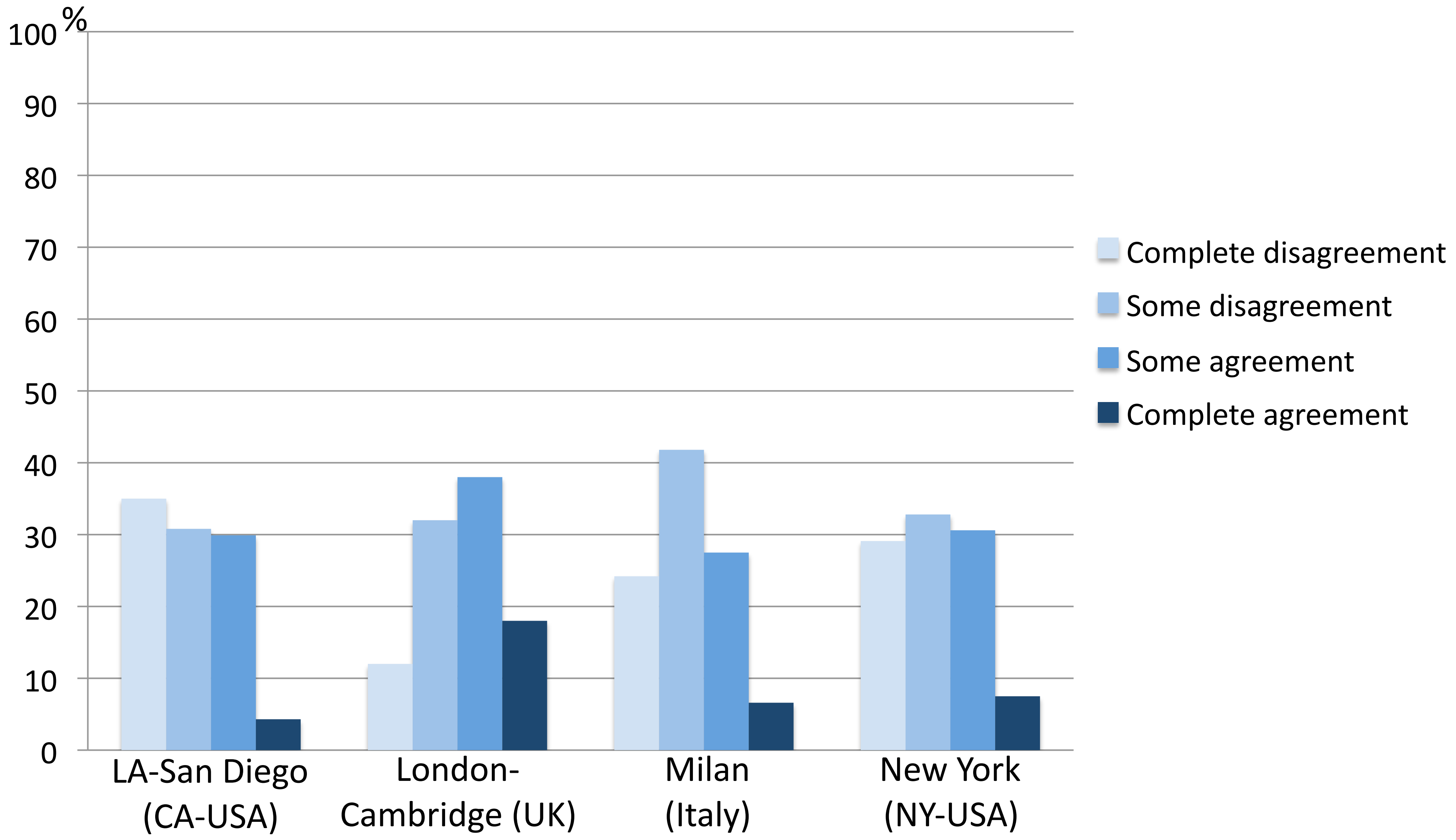

Fig. S25a

	Complete disagreement	Some disagreement	Some agreement	Complete agreement	Total
**LA-SD (CA-USA)**	35	30.8	29.9	4.3	100
**London-Camb. (UK)**	12	32	38	18	100
**Milan (Italy)**	24.2	41.8	27.5	6.6	100
**NYC (NY-USA)**	29.1	32.8	30.6	7.5	100
Total	25.8	33.9	31.4	8.8	100

0-100%
Basic Research

	Complete disagreement	Some disagreement	Some agreement	Complete agreement	Total
**LA-SD (CA-USA)**	27.8	36.7	32.9	2.5	100
**London-Camb. (UK)**	8.8	22.8	43.9	24.6	100
**Milan (Italy)**	8	44	40	8	100
**NYC (NY-USA)**	27.1	27.1	38.8	7.1	100
Total	21.1	30.9	38.2	9.8	100

81-100%
Basic Research

Fig. S25a

The sections discussing potential future health benefits should be eliminated for .... grants.
Principal Investigators ordered by geographical location

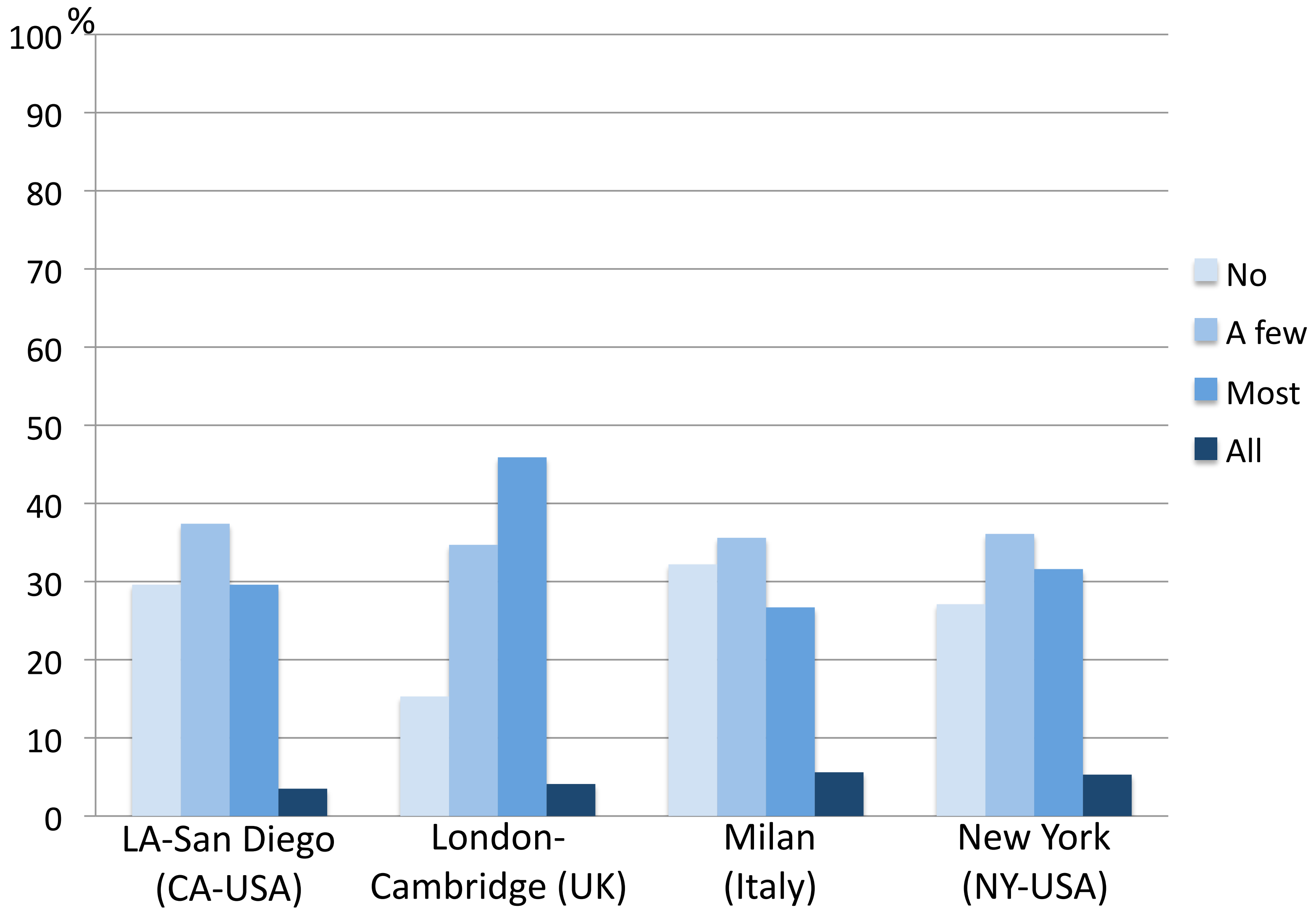

Fig. S25b

	...no...	...a few...	...most...	...all...	Total	
**LA-SD (CA-USA)**	29.6	37.4	29.6	3.5	100	
**London-Camb. (UK)**	15.3	34.7	45.9	4.1	100	0-100%
**Milan (Italy)**	32.2	35.6	26.7	5.6	100	Basic Research
**NYC (NY-USA)**	27.1	36.1	31.6	5.3	100	
Total	26.1	36	33.3	4.6	100	

	...no...	...a few...	...most...	...all...	Total	
**LA-SD (CA-USA)**	26.9	35.9	33.3	3.8	100	
**London-Camb. (UK)**	10.7	33.9	48.2	7.1	100	81-100%
**Milan (Italy)**	19.2	26.9	50	3.8	100	Basic Research
**NYC (NY-USA)**	22.4	34.1	35.3	8.2	100	
Total	20.8	33.9	39.2	6.1	100	

Fig. S25b

A. Favorability for "Locate more basic research laboratories inside or in close proximity of hospitals"
Principal Investigators ordered by geographical location

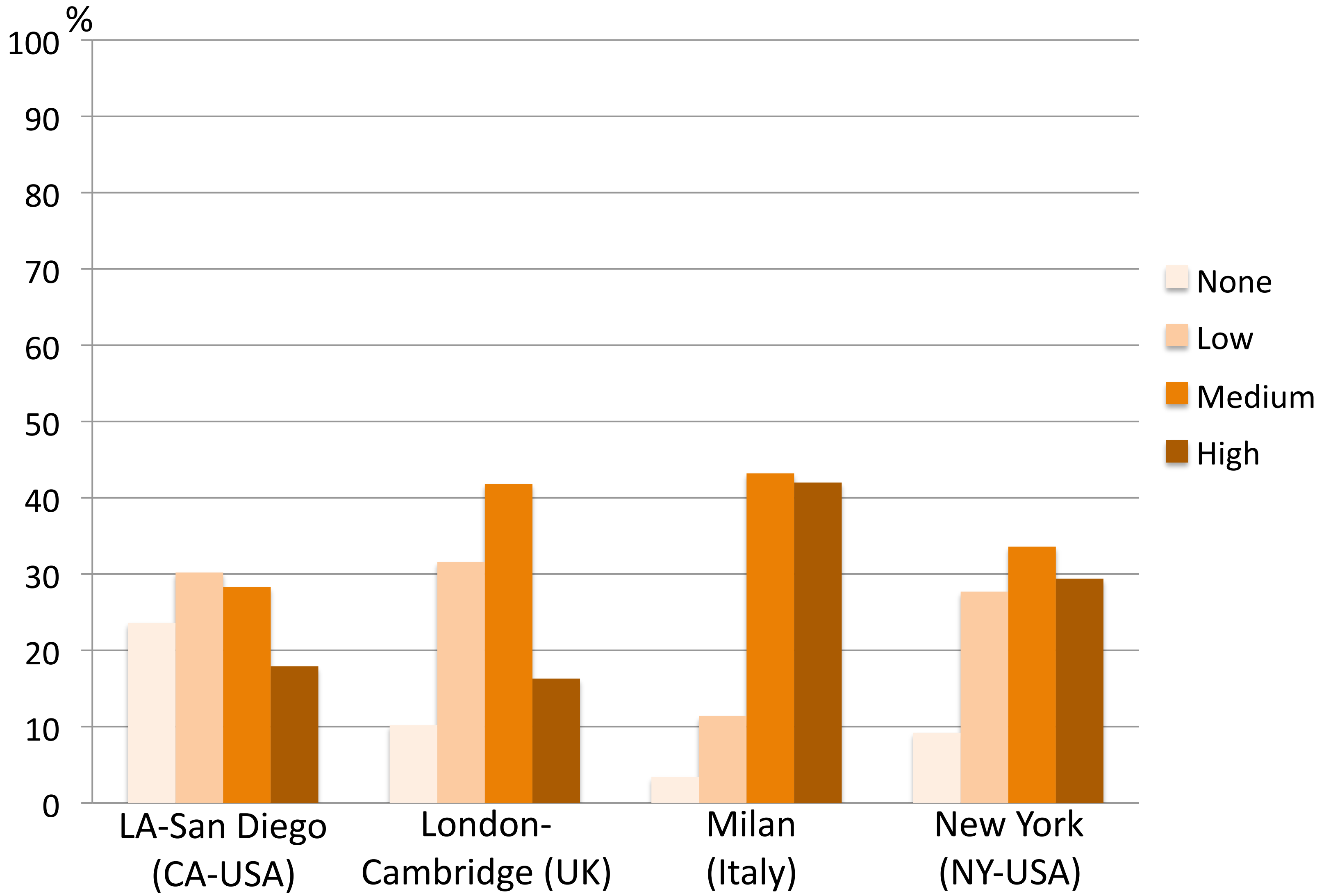

Fig. S26a

	None	Low	Medium	High	Total	
**LA-SD (CA-USA)**	23.6	30.2	28.3	17.9	100	
**London-Camb. (UK)**	10.2	31.6	41.8	16.3	100	0-100%
**Milan (Italy)**	3.4	11.4	43.2	42	100	Basic Research
**NYC (NY-USA)**	9.2	27.7	33.6	29.4	100	
Total	11.9	25.8	36.3	26	100	

	None	Low	Medium	High	Total	
**LA-SD (CA-USA)**	28.6	32.9	25.7	12.9	100	
**London-Camb. (UK)**	14.5	40	38.2	7.3	100	81-100%
**Milan (Italy)**	4.2	16.7	54.2	25	100	Basic Research
**NYC (NY-USA)**	9.3	30.7	32	28	100	
Total	16.1	32.1	33.9	17.9	100	

Fig. S26a

B. Favorability for "Organize more educational and discussion meetings between scientists and the general public or patient associations. Acknowledge participating scientists during grant assignments, promotion, hiring etc."
Principal Investigators ordered by geographical location

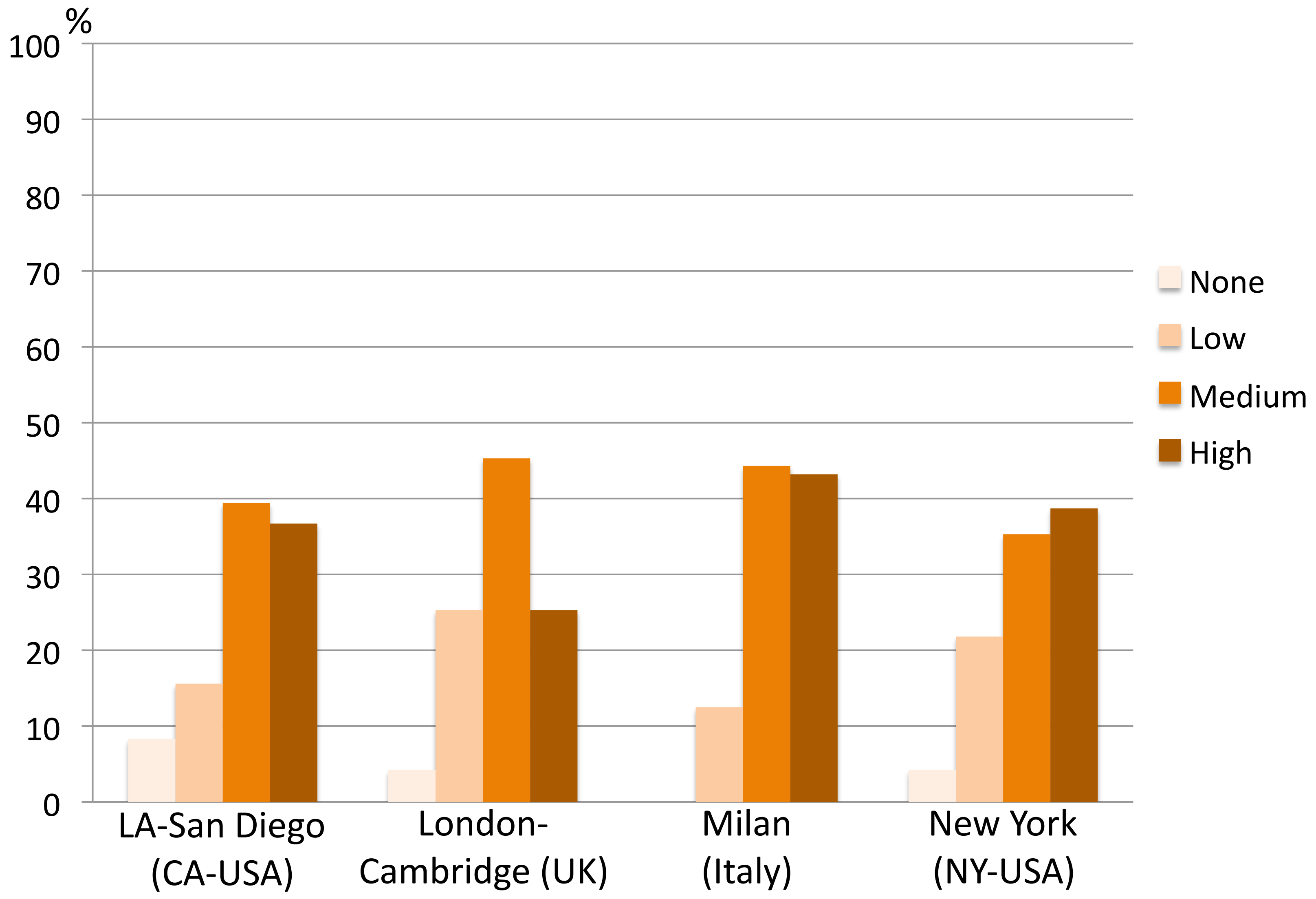

Fig. S26b

	None	Low	Medium	High	Total	
**LA-SD (CA-USA)**	8.3	15.6	39.4	36.7	100	
**London-Camb. (UK)**	4.2	25.3	45.3	25.3	100	0-100%
**Milan (Italy)**	0	12.5	44.3	43.2	100	Basic Research
**NYC (NY-USA)**	4.2	21.8	35.3	38.7	100	
Total	4.4	19	40.6	36	100	

	None	Low	Medium	High	Total	
**LA-SD (CA-USA)**	6.9	15.3	38.9	38.9	100	
**London-Camb. (UK)**	1.8	30.9	47.3	20	100	81-100%
**Milan (Italy)**	0	20	52	28	100	Basic Research
**NYC (NY-USA)**	5.3	21.3	32	41.3	100	
Total	4.4	21.6	40.1	33.9	100	

Fig. S26b

C. Favorability for "Promote more seminars and academic discussion concerning the purpose of scientific research and the role of scientists in the society. Acknowledge participating scientists during grant assignments, promotion, hiring etc."
Principal Investigators ordered by geographical location

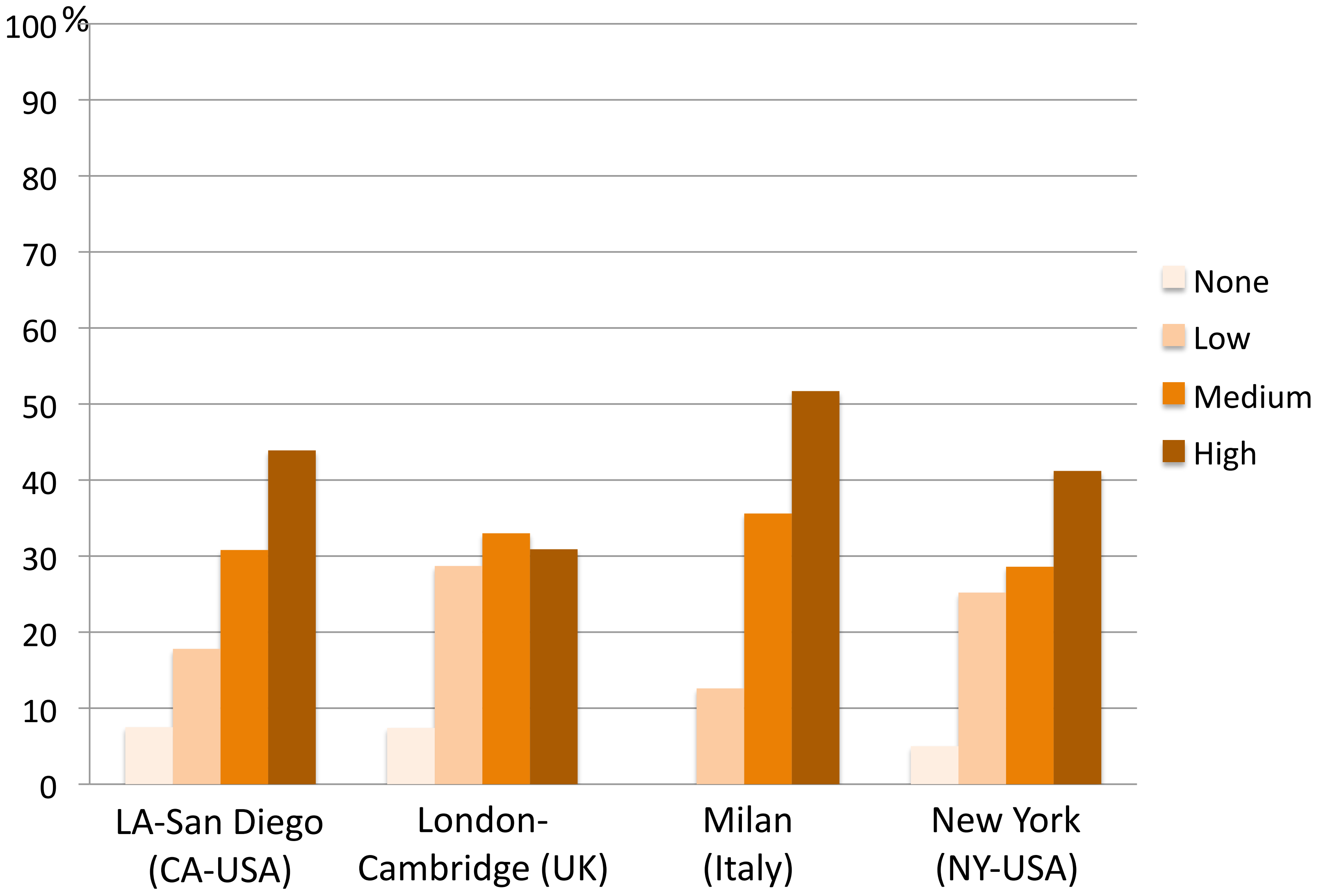

Fig. S26c

	None	Low	Medium	High	Total	
**LA-SD (CA-USA)**	7.5	17.8	30.8	43.9	100	
**London-Camb. (UK)**	7.4	28.7	33	30.9	100	0-100%
**Milan (Italy)**	0	12.6	35.6	51.7	100	Basic Research
**NYC (NY-USA)**	5	25.2	28.6	41.2	100	
Total	5.2	21.4	31.7	41.8	100	

	None	Low	Medium	High	Total	
**LA-SD (CA-USA)**	7	21.1	32.4	39.4	100	
**London-Camb. (UK)**	5.5	23.6	41.8	29.1	100	81-100%
**Milan (Italy)**	0	12.5	50	37.5	100	Basic Research
**NYC (NY-USA)**	6.7	24	29.3	40	100	
Total	5.8	21.8	35.6	36.9	100	

Fig. S26c

D. Favorability for “Promote more seminars and academic discussion about the concept and definition of basic research. Acknowledge participating scientists during grant assignments, promotion, hiring etc.” (For example, should basic research be conceptualized as purely curiosity-driven, or could basic scientists also consider future indirect practical benefits of their research?) - Principal Investigators ordered by geographical location

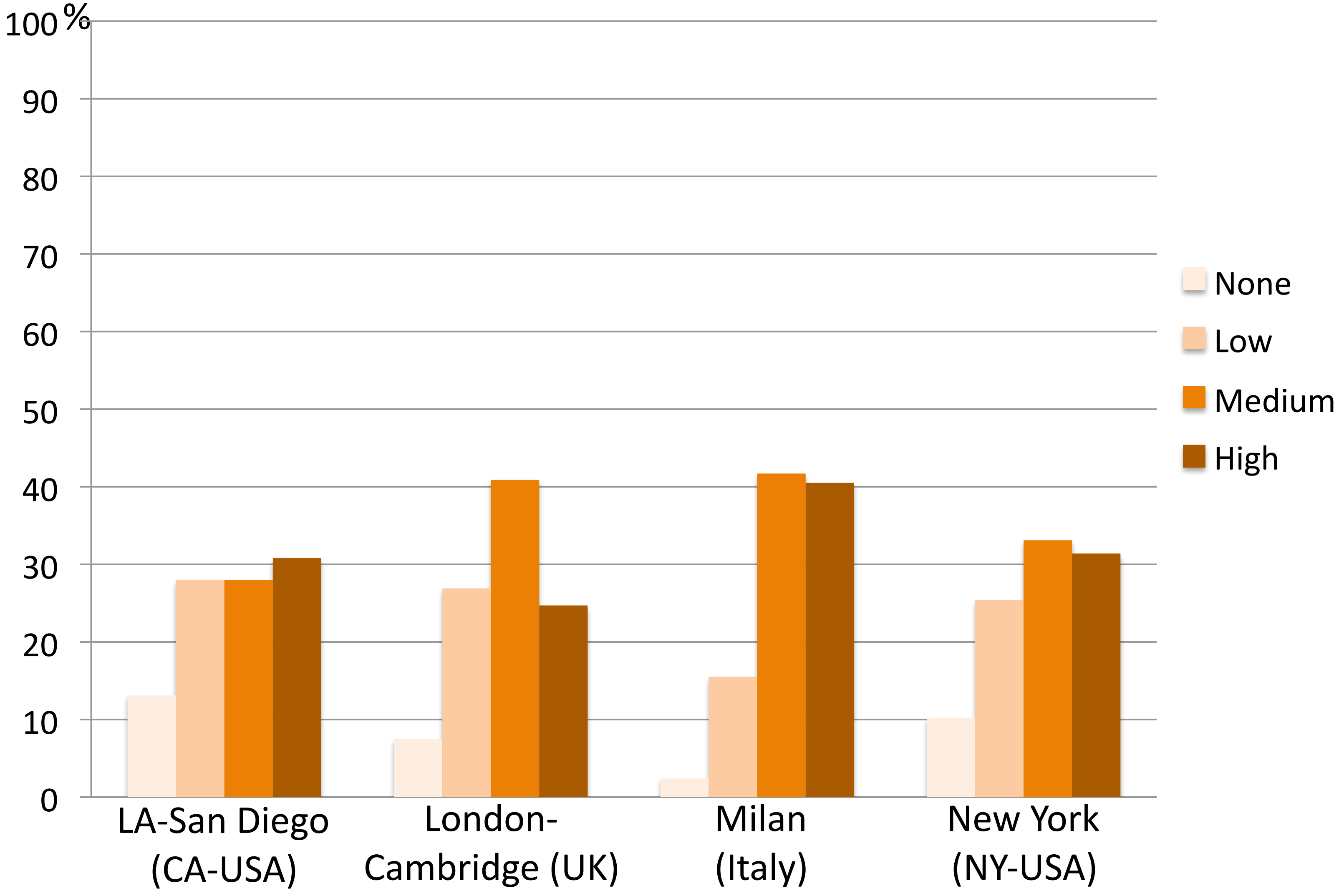

Fig. S26d

	None	Low	Medium	High	Total	
**LA-SD (CA-USA)**	13.1	28	28	30.8	100	
**London-Camb. (UK)**	7.5	26.9	40.9	24.7	100	0-100%
**Milan (Italy)**	2.4	15.5	41.7	40.5	100	Basic Research
**NYC (NY-USA)**	10.2	25.4	33.1	31.4	100	
Total	8.7	24.4	35.3	31.6	100	

	None	Low	Medium	High	Total	
**LA-SD (CA-USA)**	12.7	31	29.6	26.8	100	
**London-Camb. (UK)**	3.7	25.9	44.4	25.9	100	
**Milan (Italy)**	4.3	30.4	39.1	26.1	100	81-100%
**NYC (NY-USA)**	5.4	25.7	32.4	36.5	100	Basic Research
Total	7.2	27.9	35.1	29.7	100	

Fig. S26d

E. Favorability for "Have ethics consultation services for scientists inside research institutes, with easily accessible information about these services"
Principal Investigators ordered by geographical location

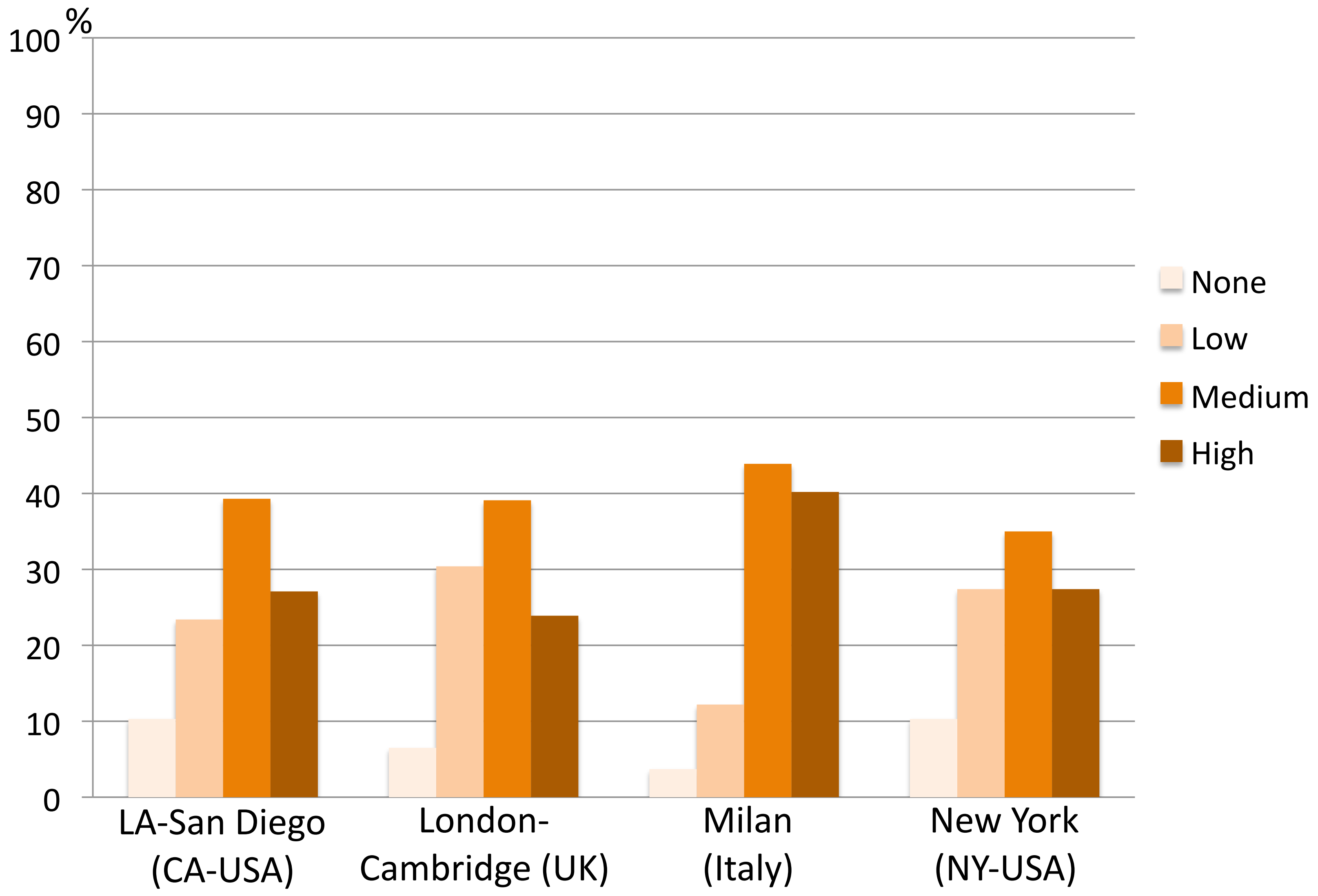

Fig. S26e

0-100% Basic Research

	**None**	**Low**	**Medium**	**High**	Total
**LA-SD (CA-USA)**	10.3	23.4	39.3	27.1	100
**London-Camb. (UK)**	6.5	30.4	39.1	23.9	100
**Milan (Italy)**	3.7	12.2	43.9	40.2	100
**NYC (NY-USA)**	10.3	27.4	35	27.4	100
Total	8	23.9	38.9	29.1	100

81-100% Basic Research

	**None**	**Low**	**Medium**	**High**	Total
**LA-SD (CA-USA)**	11.4	24.3	37.1	27.1	100
**London-Camb. (UK)**	5.6	37	37	20.4	100
**Milan (Italy)**	5	5	70	20	100
**NYC (NY-USA)**	9.6	27.4	39.7	23.3	100
Total	8.8	26.7	41	23.5	100

Fig. S26e

F. Favorability for “Provide recognition to basic scientists who have contributed to acquiring key knowledge that leads to tangible health benefits by requiring a "basic bibliography" of seminal basic research articles for each new drug or other biological application”
Principal Investigators ordered by geographical location

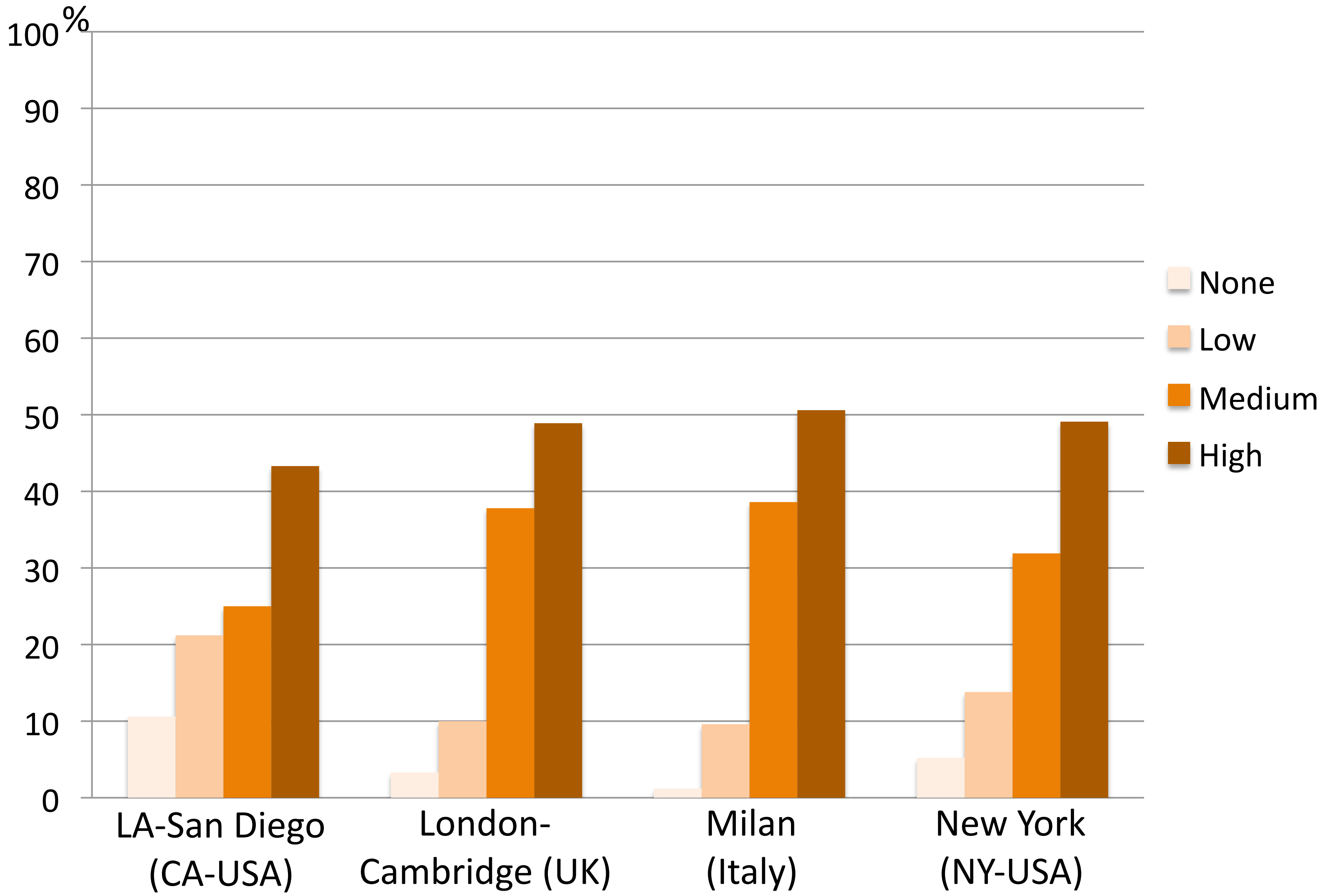

Fig. S26f

	None	Low	Medium	High	Total	
**LA-SD (CA-USA)**	10.6	21.2	25	43.3	100	
**London-Camb. (UK)**	3.3	10	37.8	48.9	100	0-100%
**Milan (Italy)**	1.2	9.6	38.6	50.6	100	Basic Research
**NYC (NY-USA)**	5.2	13.8	31.9	49.1	100	
Total	5.3	14	32.8	47.8	100	

	None	Low	Medium	High	Total	
**LA-SD (CA-USA)**	8.8	17.6	29.4	44.1	100	
**London-Camb. (UK)**	3.8	9.6	32.7	53.8	100	
**Milan (Italy)**	0	8.7	52.2	39.1	100	81-100%
**NYC (NY-USA)**	2.8	16.7	30.6	50	100	Basic Research
Total	4.7	14.4	33	47.9	100	

Fig. S26f
