# Supplementary material for: Increasing both the public health potential of basic research and the scientist satisfaction. An international survey of bio-scientists
Source: F1000Res. 2016 Jun 1;5:56. Originally published 2016 Jan 12. [Version 2] doi: 10.12688/f1000research.7683.2 (PMC4909114; doi:10.12688/f1000research.7683.2)
Supplement: Supplementary file 6 [file f1000research-5-9472-s0005.tgz › e380d2ed-f024-42c2-9f04-37257b9159f1.pdf]

Grants should not always be assigned in an 'all-or-none' fashion. Instead there should be the possibility to partially fund scientific projects/scientists so that more projects/scientists are funded even if this would decrease the numbers of projects/scientists fully funded.

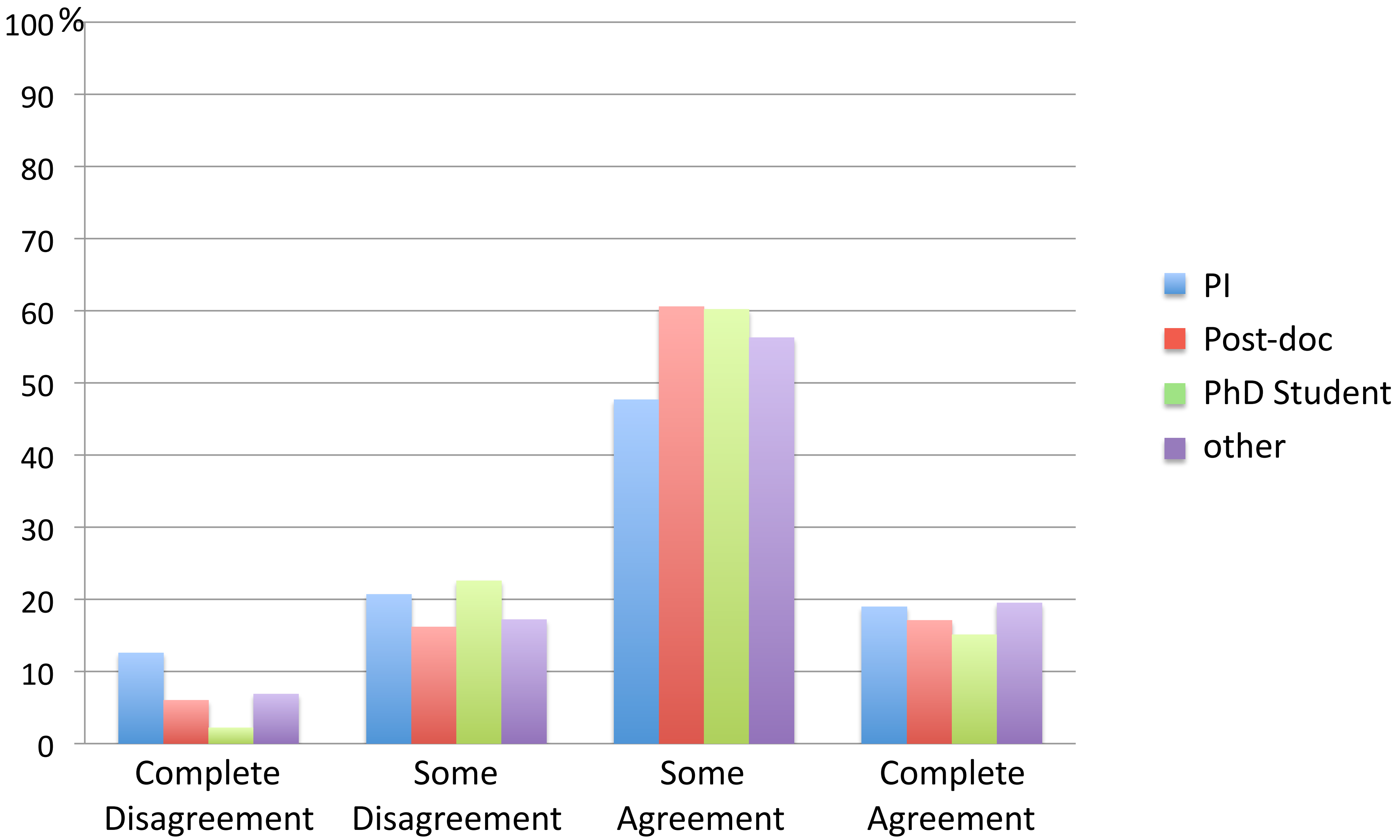

Fig. S27

	Complete disagreement	Some disagreement	Some agreement	Complete agreement	Total
**PI**	12.6	20.7	47.7	19	100
**Post-doc**	6	16.2	60.6	17.1	100
**PhD stud.**	2.2	22.6	60.2	15.1	100
**Other**	6.9	17.2	56.3	19.5	100
Total	9.2	19.4	53.2	18.1	100

Fig. S27

There should be a cap to how much a given scientist/laboratory can be funded, in order to attenuate the bias in favor of established scientists/laboratories.

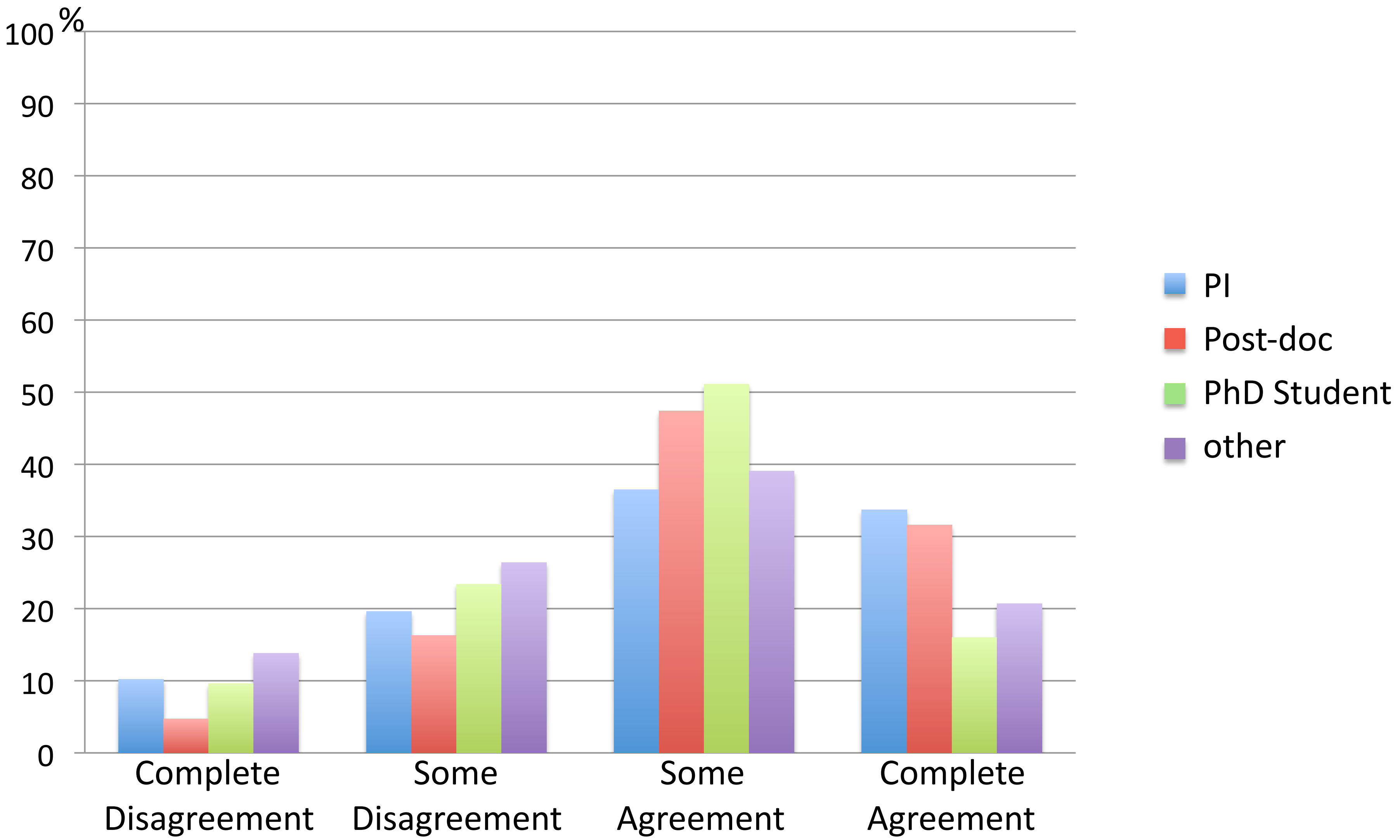

Fig. S28

	**Complete disagreement**	**Some disagreement**	**Some agreement**	**Complete agreement**	Total
**PI**	10.2	19.6	36.5	33.7	100
**Post-doc**	4.7	16.3	47.4	31.6	100
**PhD stud.**	9.6	23.4	51.1	16	100
**Other**	13.8	26.4	39.1	20.7	100
Total	9.1	19.9	41.1	29.9	100

Fig. S28

In the future, an increasing share of funding should be granted to the scientists rather than to the projects.

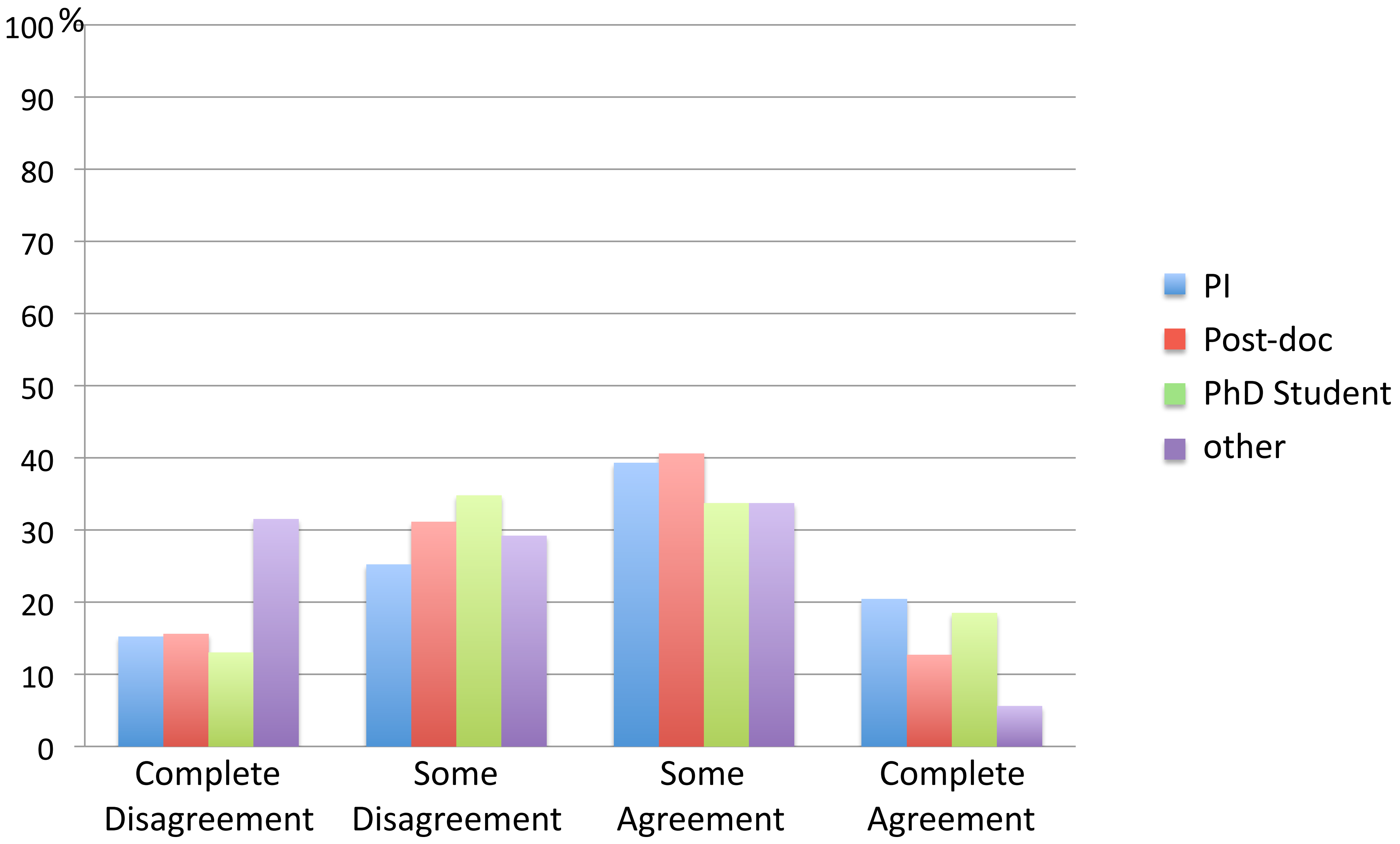

Fig. S29a

	Complete disagreement	Some disagreement	Some agreement	Complete agreement	Total
**PI**	15.2	25.2	39.3	20.4	100
**Post-doc**	15.6	31.1	40.6	12.7	100
**PhD stud.**	13	34.8	33.7	18.5	100
**Other**	31.5	29.2	33.7	5.6	100
Total	16.7	28.1	38.4	16.7	100

Fig. S29a

In the future, an increasing share of funding should be granted to the scientists rather than to the projects.

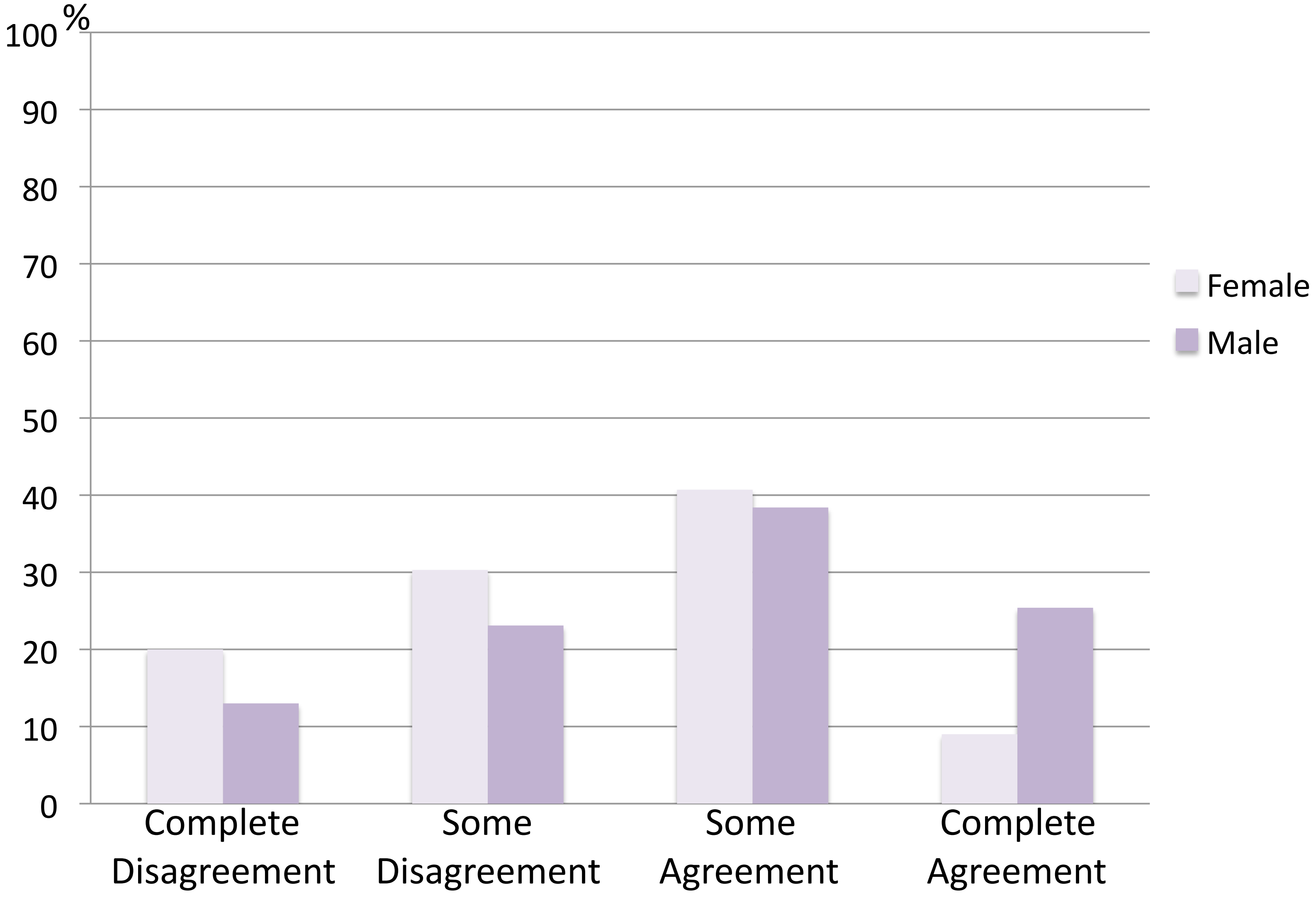

Fig. S29b

	Complete disagreement	Some disagreement	Some agreement	Complete agreement	Total
**Female**	20	30.3	40.7	9	100
**Male**	13	23.1	38.4	25.4	100
Total	15.3	25.4	39.2	20.1	100

Fig. S29b

In the future, an increasing share of funding should be granted to the scientists rather than to the projects.
Principal Investigators ordered by geographical location

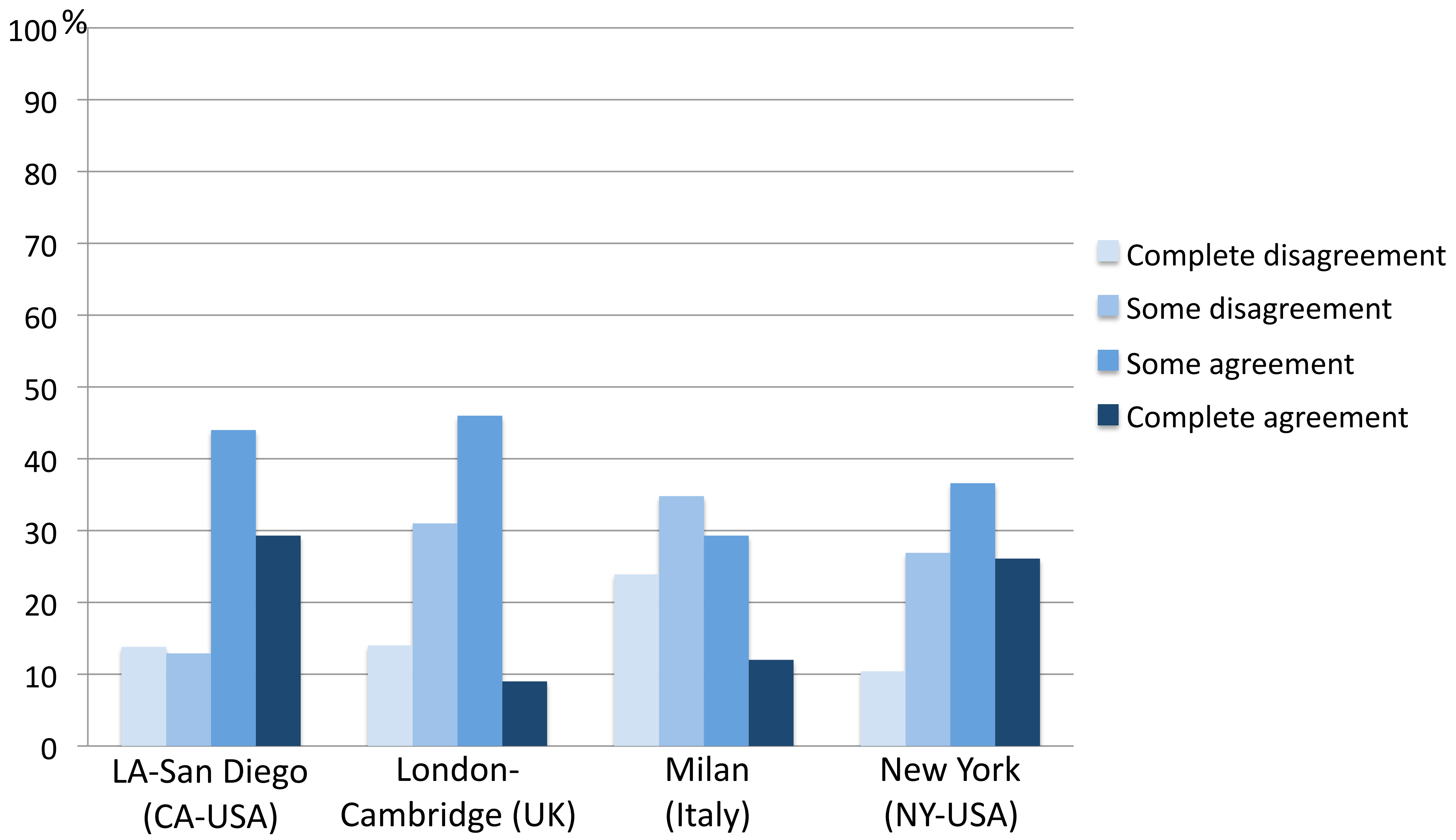

Fig. S30

	Complete disagreement	Some disagreement	Some agreement	Complete agreement	Total	
**LA-SD (CA-USA)**	13.8	12.9	44	29.3	100	
**London-Camb. (UK)**	14	31	46	9	100	0-100%
**Milan (Italy)**	23.9	34.8	29.3	12	100	Basic Research
**NYC (NY-USA)**	10.4	26.9	36.6	26.1	100	
Total	14.9	25.8	39.1	20.1	100	

	Complete disagreement	Some disagreement	Some agreement	Complete agreement	Total	
**LA-SD (CA-USA)**	15.2	11.4	45.6	27.8	100	
**London-Camb. (UK)**	17.5	28.1	47.4	7	100	
**Milan (Italy)**	19.2	34.6	38.5	7.7	100	81-100%
**NYC (NY-USA)**	7.1	28.2	38.8	25.9	100	Basic Research
Total	13.4	23.5	42.9	20.2	100	

Fig. S30
