# Supplementary material for: Increasing both the public health potential of basic research and the scientist satisfaction. An international survey of bio-scientists
Source: F1000Res. 2016 Jun 1;5:56. Originally published 2016 Jan 12. [Version 2] doi: 10.12688/f1000research.7683.2 (PMC4909114; doi:10.12688/f1000research.7683.2)
Supplement: Supplementary file 7 [file f1000research-5-9472-s0006.tgz › 1557be2e-8c81-40ae-a7b2-361172b8a16e.pdf]

	Not a motivation	Minimally important	Moderately important	Important	Very important	Total
**PI**	2.2	4.3	13.6	36.3	43.6	100
**Post-doc**	4.2	7.4	16.7	42.6	29.2	100
**PhD stud.**	4.3	8.6	28	41.9	17.2	100
**Other**	4.5	7.9	20.2	48.3	19.1	100
Total	3.1	5.9	16.6	39.7	34.6	100

Fig. 1a

	Not a motivation	Minimally important	Moderately important	Important	Very important	Total
**PI**	1.3	3.7	16.6	39.1	39.3	100
**Post-doc**	1.9	4.6	14.8	34.7	44	100
**PhD stud.**	2.2	2.2	10.8	46.2	38.7	100
**Other**	1.1	1.1	8	35.2	54.5	100
Total	1.5	3.5	14.7	38.4	42	100

Fig. 1b

	**Not a motivation**	**Minimally important**	**Moderately important**	**Important**	**Very important**	Total
**PI**	20.2	32.1	28.9	17.1	1.7	100
**Post-doc**	24.5	23.6	34.7	15.7	1.4	100
**PhD stud.**	20.4	31.2	37.6	8.6	2.2	100
**Other**	21.3	32.6	27	16.9	2.2	100
Total	21.4	29.9	31.1	15.8	1.7	100

Fig. 1c

	Not a motivation	Minimally important	Moderately important	Important	Very important	Total
**PI**	40.2	32.4	21.3	5.7	0.4	100
**Post-doc**	28.6	26.7	25.8	16.1	2.8	100
**PhD stud.**	27.5	30.8	27.5	11	3.3	100
**Other**	28.1	32.6	25.8	11.2	2.2	100
Total	34.7	30.8	23.6	9.5	1.5	100

Fig. 1d

	Not a motivation	Minimally important	Moderately important	Important	Very important	Total
**PI**	0.6	1.7	9.3	30.3	58	100
**Post-doc**	0.9	2.8	8.8	36.4	51.2	100
**PhD stud.**	0	1.1	12.9	47.3	38.7	100
**Other**	2.2	2.2	13.5	47.2	34.8	100
Total	0.8	2	10	35.4	51.8	100

Fig. 1e

	Not a motivation	Minimally important	Moderately important	Important	Very important	Total
**PI**	1.1	1.9	10.6	29.4	56.9	100
**Post-doc**	0.9	1.4	11.6	35.2	50.9	100
**PhD stud.**	1.1	4.3	15.1	36.6	43	100
**Other**	1.1	1.1	19.1	41.6	37.1	100
Total	1	2	12.2	32.9	51.9	100

Fig. 1f

	Not a motivation	Minimally important	Moderately important	Important	Very important	Total
**0-20%**	8.7	17.4	30.4	43.5	0	100
**21-40%**	0	6.7	33.3	46.7	13.3	100
**41-60%**	3.9	7.8	21.6	37.3	29.4	100
**61-80%**	1	7.9	11.9	40.6	38.6	100
**81-100%**	2	0.8	9	32.2	56.1	100
Total	2.2	4.3	13.7	36.1	43.7	100

Fig. 2a

	Not a motivation	Minimally important	Moderately important	Important	Very important	Total
**0-20%**	0	0	0	34.8	65.2	100
**21-40%**	0	0	0	40	60	100
**41-60%**	0	0	7.8	29.4	62.7	100
**61-80%**	0	1	20.8	37.6	40.6	100
**81-100%**	2.4	6.3	20.4	41.6	29.4	100
Total	1.3	3.7	16.7	38.9	39.3	100

Fig. 2b

	Not a motivation	Minimally important	Moderately important	Important	Very important	Total
**0-20%**	21.7	8.7	26.1	30.4	13	100
**21-40%**	10	46.7	33.3	10	0	100
**41-60%**	5.9	39.2	41.2	9.8	3.9	100
**61-80%**	22	35	28	14	1	100
**81-100%**	23.6	29.9	26	19.7	0.8	100
Total	20.3	32.1	28.6	17.2	1.7	100

Fig. 2c

	Not a motivation	Minimally important	Moderately important	Important	Very important	Total
**0-20%**	26.1	13	34.8	26.1	0	100
**21-40%**	33.3	36.7	26.7	3.3	0	100
**41-60%**	36	34	20	10	0	100
**61-80%**	44	29	21	6	0	100
**81-100%**	42.1	33.9	20.1	3.1	0.8	100
Total	40.5	31.9	21.4	5.7	0.4	100

Fig. 2d

	Not a motivation	Minimally important	Moderately important	Important	Very important	Total
**0-20%**	0	4.5	27.3	40.9	27.3	100
**21-40%**	0	6.7	6.7	26.7	60	100
**41-60%**	0	2	11.8	43.1	43.1	100
**61-80%**	0	1	13.9	31.7	53.5	100
**81-100%**	1.2	1.2	5.9	26.7	65.1	100
Total	0.7	1.7	9.4	30.3	58	100

Fig. 2e

	Not a motivation	Minimally important	Moderately important	Important	Very important	Total
**0-20%**	0	13	26.1	26.1	34.8	100
**21-40%**	3.3	3.3	26.7	20	46.7	100
**41-60%**	2	0	7.8	43.1	47.1	100
**61-80%**	1	4	5.9	32.7	56.4	100
**81-100%**	0.8	0.4	9.8	26.4	62.6	100
Total	1.1	2	10.7	29.2	57.1	100

Fig. 2f

Percentage (%)	**Complete disagreement**	**Some disagreement**	**Some agreement**	**Complete agreement**	Total
**PI**	0.2	4.2	31.1	64.5	100
**Post-doc**	1.5	6.4	41.4	50.7	100
**PhD student**	1.1	7.9	42.7	48.3	100
**Other**	1.2	4.7	48.2	45.9	100
Total	0.7	5.2	36.6	57.5	100

Fig. 3

Percentage (%)	Health benefit to society (not necessarily in the near future)	Pure advancement of knowledge, regardless of future applicability	Other	Total
**PI**	14	77.9	8.1	100
**Post-doc**	21.9	73.5	4.7	100
**PhD stud.**	31.2	65.6	3.2	100
**Other**	20.7	75.9	3.4	100
Total	18.6	75.2	6.2	100

Fig. 4a

Percentage (%)	**Health benefit to society (not necessarily in the near future)**	**Pure advancement of knowledge, regardless of future applicability**	**Other**	Total
**PI**	70.7	20.7	8.7	100
**Post-doc**	85.1	11.6	3.3	100
**PhD stud.**	87.1	8.6	4.3	100
**Other**	86.2	5.7	8	100
Total	77.7	15.6	6.8	100

Fig. 4b

	**Complete disagreement**	**Some disagreement**	**Some agreement**	**Complete agreement**	Total
**0-20%**	0	4.8	52.4	42.9	100
**21-40%**	0	3.3	40	56.7	100
**41-60%**	0	12	40	48	100
**61-80%**	1	4	31.7	63.4	100
**81-100%**	0	2.8	26.5	70.7	100
Total	0.2	4.2	31.3	64.3	100

Fig. 5

	**Health benefit to society (not necessarily in the near future)**	**Pure advancement of knowledge, regardless of future applicability**	**Other**	Total
**0-20%**	57.1	42.9	0	100
**21-40%**	16.7	70	13.3	100
**41-60%**	22	70	8	100
**61-80%**	12.1	82.8	5.1	100
**81-100%**	8.7	81.8	9.5	100
Total	13.7	78.1	8.2	100

Fig. 6a

	**Health benefit to society (not necessarily in the near future)**	**Pure advancement of knowledge, regardless of future applicability**	**Other**	Total
**0-20%**	100	0	0	100
**21-40%**	76.7	13.3	10	100
**41-60%**	78.4	9.8	11.8	100
**61-80%**	82	13	5	100
**81-100%**	61.3	28.5	10.3	100
Total	70.7	20.6	8.8	100

Fig. 6b

	**Complete disagreement**	**Some disagreement**	**Some agreement**	**Complete agreement**	Total
**PI**	11.1	20.4	50.2	18.3	100
**Post-doc**	6	16.7	57.2	20	100
**PhD stud.**	5.4	20.4	59.1	15.1	100
**Other**	5.7	14.8	53.4	26.1	100
Total	8.6	18.9	53.3	19.2	100

Fig. 7

	**Complete disagreement**	**Some disagreement**	**Some agreement**	**Complete agreement**	Total
**PI**	27.2	30.5	36.2	6.1	100
**Post-doc**	16.3	27.9	40.9	14.9	100
**PhD stud.**	16.1	31.2	33.3	19.4	100
**Other**	17	25	43.2	14.8	100
Total	22.2	29.4	37.8	10.6	100

Fig. 8

	Complete disagreement	Some disagreement	Some agreement	Complete agreement	Total
**PI**	26	33.6	31.2	9.1	100
**Post-doc**	17.9	36.3	37.7	8	100
**PhD stud.**	18.5	52.2	21.7	7.6	100
**Other**	24.4	33.7	36	5.8	100
Total	23	36.3	32.3	8.3	100

Fig. 9a

	...no...	...a few...	...most...	...all...	Total
**PI**	26.4	36.3	32.7	4.6	100
**Post-doc**	18.7	55.6	22.9	2.8	100
**PhD stud.**	31.5	43.5	19.6	5.4	100
**Other**	38.4	45.3	16.3	0	100
Total	26.2	42.9	27.2	3.8	100

Fig. 9b

	Complete disagreement	Some disagreement	Some agreement	Complete agreement	Total
**0-20%**	4.3	4.3	56.5	34.8	100
**21-40%**	3.4	24.1	44.8	27.6	100
**41-60%**	2	23.5	49	25.5	100
**61-80%**	11	15	56	18	100
**81-100%**	14.5	23.1	48.2	14.1	100
Total	11.1	20.5	50.2	18.1	100

Fig. 10

	Complete disagreement	Some disagreement	Some agreement	Complete agreement	Total
**0-20%**	8.7	8.7	65.2	17.4	100
**21-40%**	13.3	36.7	46.7	3.3	100
**41-60%**	14	34	42	10	100
**61-80%**	28.3	21.2	42.4	8.1	100
**81-100%**	32.5	34.5	29	3.9	100
Total	27.1	30.4	36.3	6.1	100

Fig. 11

	Complete disagreement	Some disagreement	Some agreement	Complete agreement	Total
**0-20%**	56.5	39.1	4.3	0	100
**21-40%**	36.7	46.7	10	6.7	100
**41-60%**	33.3	31.4	25.5	9.8	100
**61-80%**	25.7	34.7	30.7	8.9	100
**81-100%**	20.9	31.1	37.8	10.2	100
Total	26.1	33.3	31.4	9.2	100

Fig. 12a

	**...no...**	**...a few...**	**...most...**	**...all...**	Total
**0-20%**	54.5	36.4	4.5	4.5	100
**21-40%**	43.3	46.7	10	0	100
**41-60%**	31.4	45.1	21.6	2	100
**61-80%**	25.8	33	37.1	4.1	100
**81-100%**	20.9	34.4	38.7	5.9	100
Total	26.3	36.2	32.9	4.6	100

Fig. 12b

	None	Low	Medium	High	Mean
Effectiveness (societal benefit potential)	5.4%	26.6%	40.2%	27.7%	2.9
Effectiveness (scientist quality of work and satisfaction)	8.6%	27.8%	35.1%	28.5%	2.8
Feasibility	2.9%	28.8%	48.3%	20.0%	2.9
Favorability for the policy	10.4%	23.8%	36.3%	29.6%	2.9
Mean	6.83%	26.75%	39.98%	26.45%	

Fig. 13a

	None	Low	Medium	High	Mean
Effectiveness (societal benefit potential)	1.5%	13.0%	39.7%	45.8%	3.3
Effectiveness (scientist quality of work and satisfaction)	4.5%	20.9%	42.3%	32.2%	3
Feasibility	1.4%	19.2%	52.9%	26.5%	3
Favorability for the policy	3.8%	15.3%	40.5%	40.4%	3.2
Mean	2.8%	17.1%	43.9%	36.2%	

Fig. 13b

	None	Low	Medium	High	Mean
Effectiveness (societal benefit potential)	2.3%	15.9%	38.3%	43.5%	3.2
Effectiveness (scientist quality of work and satisfaction)	3.8%	22.6%	39.6%	34.0%	3
Feasibility	1.4%	18.1%	48.9%	31.6%	3.1
Favorability for the policy	3.9%	17.9%	34.2%	44.0%	3.2
Mean	2.9%	18.6%	40.3%	38.3%	

Fig. 13c

	None	Low	Medium	High	Mean
Effectiveness (societal benefit potential)	5.5%	23.8%	38.7%	31.9%	3
Effectiveness (scientist quality of work and satisfaction)	6.2%	24.5%	40.8%	28.5%	2.9
Feasibility	3.5%	20.8%	48.3%	27.5%	3
Favorability for the policy	7.0%	22.9%	37.0%	33.0%	3
Mean	5.6%	23.0%	41.2%	30.2%	

Fig. 13d

	None	Low	Medium	High	Mean
Effectiveness (societal benefit potential)	4.4%	24.7%	40.7%	30.2%	3
Effectiveness (scientist quality of work and satisfaction)	6.8%	26.9%	39.3%	27.0%	2.9
Feasibility	3.1%	18.1%	49.0%	29.9%	3.1
Favorability for the policy	6.6%	21.7%	38.8%	32.9%	3
Mean	5.2%	22.9%	42.0%	30.0%	

Fig. 13e

	None	Low	Medium	High	Mean
Effectiveness (societal benefit potential)	4.1%	17.1%	37.8%	41.0%	3.2
Effectiveness (scientist quality of work and satisfaction)	2.8%	10.4%	31.6%	55.3%	3.4
Feasibility	2.6%	17.3%	40.3%	39.7%	3.2
Favorability for the policy	4.1%	12.8%	33.3%	49.9%	3.3
Mean	3.4%	14.4%	35.8%	46.5%	

Fig. 13f

	**None**	**Low**	**Medium**	**High**	Total
**PI**	11.7	25.9	36.4	25.9	100
**Post-doc**	9.4	18.8	38.2	33.5	100
**PhD stud.**	7.9	20.2	39.3	32.6	100
**Other**	9	29.5	25.6	35.9	100
Total	10.4	23.9	36.1	29.5	100

Fig. 14a

	None	Low	Medium	High	Total
**PI**	4.4	19.1	40.8	35.7	100
**Post-doc**	2.1	12.4	42	43.5	100
**PhD stud.**	2.2	6.7	35.6	55.6	100
**Other**	6.7	12	38.7	42.7	100
Total	3.8	15.4	40.3	40.5	100

Fig. 14b

	**None**	**Low**	**Medium**	**High**	Total
**PI**	5.2	21.2	31.5	42.1	100
**Post-doc**	3.1	14.6	35.4	46.9	100
**PhD stud.**	1.1	11.2	41.6	46.1	100
**Other**	2.6	13.2	38.2	46.1	100
Total	4	17.6	34.3	44.1	100

Fig. 14c

	**None**	**Low**	**Medium**	**High**	Total
**PI**	8.6	24.6	35.3	31.5	100
**Post-doc**	6.4	21.4	33.2	39	100
**PhD stud.**	2.3	21.8	47.1	28.7	100
**Other**	5.4	17.6	44.6	32.4	100
Total	7	22.8	37	33.1	100

Fig. 14d

	**None**	**Low**	**Medium**	**High**	Total
**PI**	8.2	23.4	39.3	29.2	100
**Post-doc**	4.8	18.1	41.5	35.6	100
**PhD stud.**	5.7	20.5	37.5	36.4	100
**Other**	3.9	24.7	28.6	42.9	100
Total	6.6	21.9	38.5	32.9	100

Fig. 14e

	None	Low	Medium	High	Total
**PI**	5.1	13.9	33.4	47.6	100
**Post-doc**	2.7	11.5	33.3	52.5	100
**PhD stud.**	2.3	16.1	32.2	49.4	100
**Other**	2.7	6.7	33.3	57.3	100
Total	4	12.8	33.2	49.9	100

Fig. 14f

	**None**	**Low**	**Medium**	**High**	Total
**0-20%**	0	4.8	42.9	52.4	100
**21-40%**	3.6	21.4	32.1	42.9	100
**41-60%**	8.2	16.3	40.8	34.7	100
**61-80%**	7.2	21.6	41.2	29.9	100
**81-100%**	16	32	33.8	18.2	100
Total	11.5	25.8	36.6	26.1	100

Fig. 15a

	**None**	**Low**	**Medium**	**High**	Total
**0-20%**	9.5	14.3	28.6	47.6	100
**21-40%**	3.7	18.5	44.4	33.3	100
**41-60%**	6.4	21.3	40.4	31.9	100
**61-80%**	2	13.3	44.9	39.8	100
**81-100%**	4.7	21.8	39.7	33.8	100
Total	4.4	19.2	40.7	35.6	100

Fig. 15b

	**None**	**Low**	**Medium**	**High**	Total
**0-20%**	9.5	19	19	52.4	100
**21-40%**	0	19.2	38.5	42.3	100
**41-60%**	2.1	27.7	27.7	42.6	100
**61-80%**	4.1	17.5	25.8	52.6	100
**81-100%**	6	22	34.9	37.1	100
Total	5	21.3	31.4	42.3	100

Fig. 15c

	None	Low	Medium	High	Total
**0-20%**	15	20	40	25	100
**21-40%**	10.7	17.9	35.7	35.7	100
**41-60%**	12.8	23.4	38.3	25.5	100
**61-80%**	6.5	20.4	33.3	39.8	100
**81-100%**	7.4	27.5	35.4	29.7	100
Total	8.4	24.5	35.5	31.7	100

Fig. 15d

	None	Low	Medium	High	Total
**0-20%**	0	20	35	45	100
**21-40%**	7.1	14.3	39.3	39.3	100
**41-60%**	8.7	28.3	28.3	34.8	100
**61-80%**	6.3	17.9	42.1	33.7	100
**81-100%**	9.4	26.3	40.6	23.7	100
Total	8	23.5	39.2	29.3	100

Fig. 15e

	**None**	**Low**	**Medium**	**High**	Total
**0-20%**	5	25	35	35	100
**21-40%**	7.4	14.8	37	40.7	100
**41-60%**	4.3	10.9	37	47.8	100
**61-80%**	6.5	10.8	30.1	52.7	100
**81-100%**	4.5	14.9	32.9	47.7	100
Total	5.1	14	33.1	47.8	100

Fig. 15f

	**None**	**Low**	**Medium**	**High**	Total	
**Female**	10.8	30.9	33.8	24.5	100	0-100%
**Male**	12.1	23.4	37.6	27	100	Basic Research
Total	11.6	25.9	36.3	26.1	100	

	**None**	**Low**	**Medium**	**High**	Total	
**Female**	11	39	30.5	19.5	100	81-100%
**Male**	18.6	27.6	36.6	17.2	100	Basic Research
Total	15.9	31.7	34.4	18.1	100	

Fig. 16a

	None	Low	Medium	High	Total	
**Female**	1.4	15.2	43.5	39.9	100	0-100%
**Male**	5.7	21.6	39.6	33.2	100	Basic Research
Total	4.3	19.5	40.9	35.4	100	

	None	Low	Medium	High	Total	
**Female**	1.2	19.8	43.2	35.8	100	81-100%
**Male**	6.1	23.6	38.5	31.8	100	Basic Research
Total	4.4	22.3	40.2	33.2	100	

Fig. 16b

	None	Low	Medium	High	Total
**Female**	3	18.5	33.3	45.2	100
**Male**	6	22.6	30.7	40.6	100
Total	5	21.3	31.6	42.1	100

0-100%
Basic Research

	None	Low	Medium	High	Total
**Female**	3.8	22.5	33.8	40	100
**Male**	6.8	22.3	35.1	35.8	100
Total	5.7	22.4	34.6	37.3	100

81-100%
Basic Research

Fig. 16c

	**None**	**Low**	**Medium**	**High**	Total	
**Female**	5.2	22.2	37	35.6	100	0-100% Basic Research
**Male**	10.5	25.3	34.7	29.6	100	
Total	8.7	24.3	35.4	31.6	100	

	**None**	**Low**	**Medium**	**High**	Total	
**Female**	5.1	21.5	39.2	34.2	100	81-100% Basic Research
**Male**	8.9	29.5	33.6	28.1	100	
Total	7.6	26.7	35.6	30.2	100	

Fig. 16d

	None	Low	Medium	High	Total	
**Female**	4.5	20.5	44.7	30.3	100	0-100% Basic Research
**Male**	9.8	24.6	36.6	29	100	
Total	8.1	23.3	39.2	29.4	100	

	None	Low	Medium	High	Total	
**Female**	4.1	28.4	50	17.6	100	81-100% Basic Research
**Male**	11.6	25.3	35.6	27.4	100	
Total	9.1	26.4	40.5	24.1	100	

Fig. 16e

	None	Low	Medium	High	Total	
**Female**	5.3	9.9	29	55.7	100	
**Male**	4.8	16.1	35.2	44	100	0-100% Basic Research
Total	5	14.1	33.2	47.8	100	

	None	Low	Medium	High	Total	
**Female**	5.3	9.2	28.9	56.6	100	
**Male**	3.5	18.2	35	43.4	100	81-100% Basic Research
Total	4.1	15.1	32.9	47.9	100	

Fig. 16f
