# Supplementary material for: Increasing both the public health potential of basic research and the scientist satisfaction. An international survey of bio-scientists
Source: F1000Res. 2016 Jun 1;5:56. Originally published 2016 Jan 12. [Version 2] doi: 10.12688/f1000research.7683.2 (PMC4909114; doi:10.12688/f1000research.7683.2)
Supplement: Questions and responses of the survey — “QUESTIONNAIRE.pdf” contains the questions of the questionnaire. “QUESTIONNAIRE DATA.csv” contains the responses of the questionnaire. “FINAL REPORT.pdf” contains the summary of the responses of the questionnaire. [file f1000research-5-9472-s0000.tgz › e2cce3ea-006d-4143-befe-10b5dd86c3e2_Data.zip!/Data/FINAL REPORT.pdf]

My Report

Last Modified: 11/19/2015

1. Q1 About you...Please indicate which of the following best describes you:

Answer	Bar	Response	%
Principal investigator		464	53.6%
Post-doc		219	25.3%
PhD student		94	10.9%
Other (please specify)		89	10.3%
Total		866	

Other (please specify)
Research Administration
staff scientist
staff scientist
Staff scientist
Clinical lecturer (50% clinical, 50% research)
Assistant Professor, Co-PI
Reaserch and teaching associate
Postgraduate research student
Research Assistant
Staff Scientist
Staff Scientist
Research Assisstant
Senior Research Scientist
Retired, but still active
Emeritus Professor
Research Associate
Research student
senior scientist
Technician
Research assistant
Technician
Head of unit
Research fellow
student of master'degree
Student
Emeritus Professor
Lab Manager
Research specialist
Student Researcher
researcher
Bachelor of science
technologist
Group Leader
MD
student
scholarship holder
Master's degree student
senior researcher
Researcher
consultant
3 years fellowship

STUDENT
Post-Graduate Student
Head of lab
research fellow
master student
Senior scientist
Associate Research Scientist
Attending student
co-principal investigator
Senior Clinician Scientist
Research Associate
VP data sciences
Core staff member and lecturer
Dean
non-tenure track faculty
Research Assistant
Clinical Lecturer/PostDoc researcher
Senior scientist
Postgraduate student
MD
Animal Compliance Coordinator/Voluntary Assoc. Prof.
Research Scientist
associate (non tenure track faculty)
thesys student
associate scientist
Research assistant
unit leader
Research Scientist and Program Manager
Research Faculty
Guest
Assistant Professor
Staff scientist
thesys student
science writer
MSc graduate student
Recently retired
Retired researcher
P.I. until recently
senior scientist
Lecturer
Staff scientist
Independent fellow
Sr. Research Associate
Honorary lecturer
Senior Resercher-staff scientist
Associate Scientist
Research Associate
Research assistant

Statistic	Value
Min Value	1
Max Value	4
Mean	1.78
Variance	1.01
Standard Deviation	1.00
Total Responses	866

2. Your gender:

#	Answer	Bar	Response	%
1	Female		359	41.8%
2	Male		500	58.2%
	Total		859	

Statistic	Value
Min Value	1
Max Value	2
Mean	1.58
Variance	0.24
Standard Deviation	0.49
Total Responses	859

3. Your age:

#	Answer	Min Value	Max Value	Average Value	Standard Deviation	Responses
1	Years:	20.00	93.00	43.41	13.39	841

4. Q2 In which of the following geographical areas do you work most of the time?

Answer	Total Responses
LA-San Diego (CA-USA)	202
London-Cambridge (UK)	180
Milan (Italy)	223
New York (NY-USA)	238
Total	885

Statistic	
Most Common	New York (NY-USA) (28.23%)
Total Responses	843

5. Q3 Approximately, what percentage of your research do you consider to be basic?

#	Answer	Min Value	Max Value	Average Value	Standard Deviation	Responses
1	%	0.00	100.00	78.02	24.85	862

6. Q4 Grants should not always be assigned in an ‘all-or-none’ fashion. Instead there should be the possibility to partially fund scientific projects/scientists so that more projects/scientists are funded even if this would decrease the numbers of projects/scientists fully funded.

#	Answer	Bar	Response	%
1	Complete disagreement		79	9%
2	Some disagreement		169	20%
3	Some agreement		456	53%
4	Complete agreement		155	18%
	Total		859	

Statistic	Value
Min Value	1
Max Value	4
Mean	2.80
Variance	0.71
Standard Deviation	0.84
Total Responses	859

7. Q5 There should be a cap to how much a given scientist/laboratory can be funded, in order to attenuate the bias in favor of established scientists/laboratories.

#	Answer	Bar	Response	%
1	Complete disagreement		78	9%
2	Some disagreement		172	20%
3	Some agreement		353	41%
4	Complete agreement		257	30%
	Total		860	

Statistic	Value
Min Value	1
Max Value	4
Mean	2.92
Variance	0.86
Standard Deviation	0.93
Total Responses	860

8. Q6 In the future, an increasing share of funding should be granted to the scientists rather than to the projects.

#	Answer	Bar	Response	%
1	Complete disagreement		143	17%
2	Some disagreement		242	28%
3	Some agreement		330	38%
4	Complete agreement		143	17%
	Total		858	

Statistic	Value
Min Value	1
Max Value	4
Mean	2.55
Variance	0.92
Standard Deviation	0.96
Total Responses	858

9. Q7 Basic scientists can ponder about the future indirect practical benefits of their research without losing their "basic status".

#	Answer	Bar	Response	%
1	Complete disagreement		6	1%
2	Some disagreement		43	5%
3	Some agreement		304	36%
4	Complete agreement		481	58%
	Total		834	

Statistic	Value
Min Value	1
Max Value	4
Mean	3.51
Variance	0.40
Standard Deviation	0.63
Total Responses	834

10. Q8 Your personal motivations as a scientist are from:

#	Question	Not a motivation	Minimally important	Moderately important	Important	Very important	Total Responses	Mean
1	Pure advancement of knowledge, regardless of future applicability	27	51	144	344	299	865	3.97
2	Health benefit to society (not necessarily in the near future)	13	30	126	332	363	864	4.16
3	Gain of prestige	185	258	269	136	15	863	2.46
4	Gain of money (for personal purposes)	297	267	203	81	13	861	2.12
5	Satisfaction of your curiosity	7	17	87	307	447	865	4.35
6	Satisfaction from solving puzzling problems	9	17	106	285	447	864	4.32

Statistic	Pure advancement of knowledge, regardless of future applicability	Health benefit to society (not necessarily in the near future)	Gain of prestige	Gain of money (for personal purposes)	Satisfaction of your curiosity	Satisfaction from solving puzzling problems
Min Value	1	1	1	1	1	1
Max Value	5	5	5	5	5	5
Mean	3.97	4.16	2.46	2.12	4.35	4.32
Variance	1.03	0.82	1.10	1.08	0.65	0.71
Standard Deviation	1.01	0.90	1.05	1.04	0.80	0.84
Total Responses	865	864	863	861	865	864

11. Q9 What should the most important goal of publicly funded basic BIOLOGICAL (not biomedical) research be?

#	Answer	Bar	Response	%
1	Health benefit to society (not necessarily in the near future)		158	18%
2	Pure advancement of knowledge, regardless of future applicability		643	75%
3	Other (please specify):		54	6%
	Total		855	

Other (please specify):
Advancement of knowledge that "speaks" to general public, even if it is not directly benefiting public health.
benefit to societies currently and in future - this might not be health focused but could be sustainability for example
These two are not exclusive. In fact, advancement of knowledge is the first step to any health benefits. How do you fix car if you don't even know what a car is, how it works, and why it breaks down? Basic research is getting those most basic information that is absolutely necessary for battling any diseases or health issues.
Advancement of knowledge - but with applications in mind - not necessarily health benefits
development of sustainable technologies; solutions to environmental problems and existential risks
I think the focus on pure advancement of knowledge is most likely to lead to ultimate health benefits
Advancement of knowledge and health benefit both should be a goal as dictated by budget restriction, one may be more important than the other depending upon stated goals of work
Sustainable advances to benefit not only human health but health of other species and ecosystems as a whole.
Advancement of knowledge, with regard to future applicability
Both goals are important and can and should be considered when funding a project.
Pure advancement of knowledge with a potential benefit to society
Balance of both
General benefit to the society (developed and developing) but also training of new scientists
Both
Serious advancements of biological knowledge always brings, sooner or later, benefits to human health
I resist the forced choice of a single goal. Both are important
Advancement in any knowledge - pure (80 %) or health (20 %). The health benefit can come form the insight of others
Improving understanding of basic processes which are taken for granted or overlooked by applied research
Getting people in science. Make discoveries not war.
It should be a balance between both pure advancement of knowledge and health benefit to society. The biggest issue is that the scientific system is overloaded
It is hard to differentiate the two statements above. With advancement of knowledge comes benefits to society though not necessarily in the near future.
both of the statements mention above
advancement with a potential to benefit society, not necessarily a health benefit for humans, but for the planet in total
also applicability
Fundamentally wrong to phrase it as a binary question
Health benefits come from basic research, not mutually exclusive
both
I do not think that you can have an either or on the two categories. Both are important because one does not know how pure advancement of knowledge will be useful in the future but is often the basis for health benefits.
both
Pure advancement of knowledge, with some path to future health benefit to society
I believe that increases in basic information are very important for future advances that will bring health benefits
I think that they should be considered equally important. I do not see this clear-cut separation as I'm not sure the first point can be easily predicted
public funds should be used for the eventual benefit of the public in some fashion.
Both the above
both are important and not incompatible
both should be considered
A combination of both
Training future generations of scientists
Both
there are so many societal benefits of research besides health and pure knowledge, this is an artificial duality!
Basic science should focus more on questions that in the short or long term will advance both knowledge and heatlh
Equal parts of both
Health benefit to society (not necessarily in the near future) and Pure advancement of knowledge, regardless of future applicability
Both are important. health benefits to society accrue more from unexpected results of basic research than targeted bomedical research.

You are presenting a false dichotomy. Knowledge always leads to benefits. The only thing that can be applied is knowledge.
mix of the two. You have no idea what knowledge might be a benefit. PS Health should be defined in a broad sense of health of the plant and all things on it.
It has to be a combination of the two. Many basic scientists, and myself in particular, believe that basic understanding of biology is needed for future translational work. So the short term goal is basic, but much of the long term goal is translational.
Broad benefit in multiple areas for society
Patient-centered research
Not sure. Favor #1 but a balance between #1 and #2 is needed since #2 often can feed into #1 eventually.
advancement of knowledge in biological processes that can then eventually be translated to biomedical applications.
None - the notion that there is a "most important goal" is nonsense. There should be diversity, even in "pure" biological research
shold always be a combination of the above

Statistic	Value
Min Value	1
Max Value	3
Mean	1.88
Variance	0.23
Standard Deviation	0.48
Total Responses	855

12. Q10 What should the most important goal of publicly funded basic BIOMEDICAL research be?

#	Answer	Bar	Response	%
1	Health benefit to society (not necessarily in the near future)		667	78%
2	Pure advancement of knowledge, regardless of future applicability		134	16%
3	Other (please specify):		58	7%
	Total		859	

Other (please specify):
A mix
a balance of the two
It should be a mix between health benefit to society and advancement of knowledge
both of the above. Health benefits require investment in curiosity driven basic research
Both
Both
Health benefit AND advancement of knowledge
Health benefits to society that are considered in a more holistic manner, taking into account their longer term impacts and potential negative consequences.
Health benefit to society in the near future (which distinguishes biomedical from basic biological research).
Projects that may involve human health but are not directly monetizable
Both goals are important and can and should be considered when funding a project.
Both
Both are important
Health advancement should be a motivation for biomedical research, but the most important and far reaching consequences come from unexpected discoveries, so targetting research should not be the be-all and end-all
Both
should be both advancement of knowledge, giving importance to the health as well as cultural benefit to the society
both
Understanding of living systems and improvement of human well-being
To me, "biomedical research" by definition is aimed towards improving medicine, so this question is tautological and my answer would contain no information.
Advancement in any knowledge - pure (20 %) or health (80 %). The health benefit can come form the insight of others
In my opinion, if it's biomedical, by definition is not "basic", but already oriented to application....
uld be a balance between both pure advancement of knowledge and health benefit to society. The biggest issue is that the scientific system is overloaded
Both work hand-in-hand though one before the other. Similar to mathematics and physics.
both of the statements mention above
There should be more flexibility than direct clinical applicability. Often very important biomedical discoveries stem from very basic fundamental scientific research.
a mix of basic science and applied science
Again, need both
HEALTH BENEFIT TO SOCIETY IN NEAR FUTURE AND LONG-TERM
I do not think that you can have an either or on the two categories. Both are important because one does not know how pure advancement of knowledge will be useful in the future but is often the basis for health benefits.
An understanding of disease mechanisms, in addition to directly translational work
Both health benefit and advancement of knowledge
As in #9 it is both because basic research is the necessary first component in bringing new health benefits.
Needs a balance of basic knowledge and health benefit - sometimes a benefit isn't obvious for decades but the basis must be laid down for anything to grow
I think that they should be considered equally important. I do not see this clear-cut separation as I'm not sure the first point can be easily predicted.
potential future applicability
again - both
a mix
Some combination of the two. Basic research is and always will be the driver of any health benefits.
A mix of both, depending on the potential outcomes
both should be considered
Both
The problem is that reasonable science will advance knowledge, one way or another. But, its affect on health is harder to predict. So, I think this dicotomizatoin of the problem does not do the question justice.
There should be a bakance between advancement of knowledge and potential health benefits.
there are so many societal benefits of research besides health and pure knowledge, this is an artificial duality!

some balance between the two. Not always easy to predict where the basic advances will be useful for health sciences
Equal parts of both
a mixture of the two options
The answer is complicated because health benefits to society only emerge from knowledge, but the public does not undertstand knowledge and only understands application. "Give them an inch and they'll take a mile."
See Q9
See my answer above.
see above
See above, it is both. No reason for one or the other, exclusively
This should cover both areas to different degrees
both of the above - you can't separate knowledge advancement form health benefits because you can't know how a basic doscovery will impact health in the future.
Patient-centered research
This question is impossible to answer, as most often BASIC research actually ends up driving advancements in translational medicine
None - the notion that there is a "most important goal" is nonsense. There should be diversity, even in basic biomedical research
Equally important: (a) answering a question of wide interest to many people and (b) general benefit to society, not just health

Statistic	Value
Min Value	1
Max Value	3
Mean	1.29
Variance	0.34
Standard Deviation	0.58
Total Responses	859

13. Q11 Although it is difficult to assess the potential future health benefits to society from basic biological/biomedical research as described in written proposals, some degree of estimation is always possible.

#	Answer	Bar	Response	%
1	Complete disagreement		74	9%
2	Some disagreement		163	19%
3	Some agreement		457	53%
4	Complete agreement		166	19%
	Total		860	

Statistic	Value
Min Value	1
Max Value	4
Mean	2.83
Variance	0.70
Standard Deviation	0.84
Total Responses	860

14. Q12 Written proposals about basic biological/biomedical research generally contain a section discussing potential future health benefits. These sections increase the likelihood that a project benefits future public health.

#	Answer	Bar	Response	%
1	Complete disagreement		192	22%
2	Some disagreement		252	29%
3	Some agreement		323	38%
4	Complete agreement		92	11%
	Total		859	

Statistic	Value
Min Value	1
Max Value	4
Mean	2.37
Variance	0.89
Standard Deviation	0.95
Total Responses	859

15. Q13 Writing the sections discussing potential future health benefits takes too much time.

#	Answer	Bar	Response	%
1	Complete disagreement		197	23%
2	Some disagreement		309	36%
3	Some agreement		276	32%
4	Complete agreement		73	9%
	Total		855	

Statistic	Value
Min Value	1
Max Value	4
Mean	2.26
Variance	0.83
Standard Deviation	0.91
Total Responses	855

16. Q14 The sections discussing potential future health benefits should be eliminated for .... grants.

#	Answer	Bar	Response	%
1	...no...		223	26%
2	...a few...		364	43%
3	...most...		232	27%
4	...all...		32	4%
	Total		851	

Statistic	Value
Min Value	1
Max Value	4
Mean	2.09
Variance	0.68
Standard Deviation	0.82
Total Responses	851

17. Timing

#	Answer	Average Value	Standard Deviation
1	First Click	130.34	1,799.98
2	Last Click	723.28	6,269.16
3	#QuestionText, TimingPageSubmit#	732.56	6,269.51
4	#QuestionText, TimingClickCount#	28.33	11.19

18. Q15 Please evaluate the following policy: “Locate more basic research laboratories inside or in close proximity of hospitals”

Question	None	Low	Medium	High	Mean
Effectiveness (societal benefit potential)	5.4%	26.6%	40.2%	27.7%	2.9
Effectiveness (scientist quality of work and satisfaction)	8.6%	27.8%	35.1%	28.5%	2.8
Feasibility	2.9%	28.8%	48.3%	20.0%	2.9
Favorability for the policy (i.e. are you in favor of this policy?)	10.4%	23.8%	36.3%	29.6%	2.9

Statistic	Effectiveness (societal benefit potential)	Effectiveness (scientist quality of work and satisfaction)	Feasibility	Favorability for the policy (i.e. are you in favor of this policy?)
Min Value	1	1	1	1
Max Value	4	4	4	4
Mean	2.90	2.84	2.85	2.85
Variance	0.75	0.88	0.58	0.93
Standard Deviation	0.87	0.94	0.76	0.96
Total Responses	793	794	791	791

19. Q16 Please evaluate the following policy: “Organize more educational and discussion meetings between scientists and the general public or patient associations. Acknowledge participating scientists during grant assignments, promotion, hiring etc.”

Question	None	Low	Medium	High	Mean
Effectiveness (societal benefit potential)	1.5%	13.0%	39.7%	45.8%	3.3
Effectiveness (scientist quality of work and satisfaction)	4.5%	20.9%	42.3%	32.2%	3.0
Feasibility	1.4%	19.2%	52.9%	26.5%	3.0
Favorability for the policy (i.e. are you in favor of this policy?)	3.8%	15.3%	40.5%	40.4%	3.2

Statistic	Effectiveness (societal benefit potential)	Effectiveness (scientist quality of work and satisfaction)	Feasibility	Favorability for the policy (i.e. are you in favor of this policy?)
Min Value	1	1	1	1
Max Value	4	4	4	4
Mean	3.30	3.02	3.05	3.18
Variance	0.56	0.71	0.51	0.68
Standard Deviation	0.75	0.84	0.71	0.82
Total Responses	795	794	792	792

20. Q17 Please evaluate the following policy:“Promote more seminars and academic discussion concerning the purpose of scientific research and the role of scientists in the society. Acknowledge participating scientists during grant assignments, promotion, hiring etc."

Question	None	Low	Medium	High	Mean
Effectiveness (societal benefit potential)	2.3%	15.9%	38.3%	43.5%	3.2
Effectiveness (scientist quality of work and satisfaction)	3.8%	22.6%	39.6%	34.0%	3.0
Feasibility	1.4%	18.1%	48.9%	31.6%	3.1
Favorability for the policy (i.e. are you in favor of this policy?)	3.9%	17.9%	34.2%	44.0%	3.2

Statistic	Effectiveness (societal benefit potential)	Effectiveness (scientist quality of work and satisfaction)	Feasibility	Favorability for the policy (i.e. are you in favor of this policy?)
Min Value	1	1	1	1
Max Value	4	4	4	4
Mean	3.23	3.04	3.11	3.18
Variance	0.63	0.72	0.54	0.74
Standard Deviation	0.80	0.85	0.74	0.86
Total Responses	793	791	788	787

21. Q18 Please evaluate the following policy: “Promote more seminars and academic discussion about the concept and definition of basic research. Acknowledge participating scientists during grant assignments, promotion, hiring etc.” (For example, should basic research be conceptualized as purely curiosity-driven, or could basic scientists also consider future indirect practical benefits of their research?).

Question	None	Low	Medium	High	Mean
Effectiveness (societal benefit potential)	5.5%	23.8%	38.7%	31.9%	3.0
Effectiveness (scientist quality of work and satisfaction)	6.2%	24.5%	40.8%	28.5%	2.9
Feasibility	3.5%	20.8%	48.3%	27.5%	3.0
Favorability for the policy (i.e. are you in favor of this policy?)	7.0%	22.9%	37.0%	33.0%	3.0

Statistic	Effectiveness (societal benefit potential)	Effectiveness (scientist quality of work and satisfaction)	Feasibility	Favorability for the policy (i.e. are you in favor of this policy?)
Min Value	1	1	1	1
Max Value	4	4	4	4
Mean	2.97	2.92	3.00	2.96
Variance	0.78	0.77	0.62	0.84
Standard Deviation	0.88	0.88	0.79	0.92
Total Responses	777	775	775	772

22. Q19 Please evaluate the following policy: “Have ethics consultation services for scientists inside research institutes, with easily accessible information about these services”

Question	None	Low	Medium	High	Mean
Effectiveness (societal benefit potential)	4.4%	24.7%	40.7%	30.2%	3.0
Effectiveness (scientist quality of work and satisfaction)	6.8%	26.9%	39.3%	27.0%	2.9
Feasibility	3.1%	18.1%	49.0%	29.9%	3.1
Favorability for the policy (i.e. are you in favor of this policy?)	6.6%	21.7%	38.8%	32.9%	3.0

Statistic	Effectiveness (societal benefit potential)	Effectiveness (scientist quality of work and satisfaction)	Feasibility	Favorability for the policy (i.e. are you in favor of this policy?)
Min Value	1	1	1	1
Max Value	4	4	4	4
Mean	2.97	2.86	3.06	2.98
Variance	0.72	0.80	0.60	0.81
Standard Deviation	0.85	0.89	0.78	0.90
Total Responses	774	774	770	773

23. Q20 Please evaluate the following policy: “Provide recognition to basic scientists who have contributed to acquiring key knowledge that leads to tangible health benefits by requiring a "basic bibliography" of seminal basic research articles for each new drug or other biological application” [e.g. http://www.the-scientist.com/?articles.view/articleNo/38689/title/Recognizing-Basic-Science-Contributions/]

Question	None	Low	Medium	High	Mean
Effectiveness (societal benefit potential)	4.1%	17.1%	37.8%	41.0%	3.2
Effectiveness (scientist quality of work and satisfaction)	2.8%	10.4%	31.6%	55.3%	3.4
Feasibility	2.6%	17.3%	40.3%	39.7%	3.2
Favorability for the policy (i.e. are you in favor of this policy?)	4.1%	12.8%	33.3%	49.9%	3.3

Statistic	Effectiveness (societal benefit potential)	Effectiveness (scientist quality of work and satisfaction)	Feasibility	Favorability for the policy (i.e. are you in favor of this policy?)
Min Value	1	1	1	1
Max Value	4	4	4	4
Mean	3.16	3.39	3.17	3.29
Variance	0.72	0.61	0.65	0.71
Standard Deviation	0.85	0.78	0.80	0.84
Total Responses	764	763	761	760

24. COMMENTSDo you have ideas about other policies that can increase both the societal benefit potential of basic research and the scientist satisfaction, without affecting the fundamental nature of basic investigation? You can use this space to tell us about them or for any additional considerations related to the themes of this survey.

Text Response
Fund the scientist
I think it is most beneficial if all PIs have access to basic "hard" fund so that they can run a small lab without constant pressure to get money. If they want, they are free to obtain more grants. But the continuous fund-raising mode which has become the norm in academia is negatively impacting creativity, mentoring quality and overall satisfaction of those involved in academic research.
I think clinicians will benefit most by talking to patient groups. Often do not ujnderstand the extent of symptoms. basic scientists will also benefit from more interactions with patients. Main goal: to decrease treatment of symptoms and to increase our understanding of disease. basic researchj tends to want to understand how things work. clinicians want to undrstand diseasej states, patients just want to feel better and also want to understand what is wrong with them.
Promote well-informed discussion of basic research discoveries and their contribution to knowledge in the media.
Discourage faculty members at "basic research" universities and institutes from spinning-off companies where the more socially relevant applied research is done. This ends up hiding the socially relevant applied research and thereby makes it appear as if it is something that "basic scientists" should not really be involved in.
Investigators have limited time, so coursework and consultations for most will not be feasible. However if outreach activities are required for certain award mechanisms (NCI Oustanding Investigator Awards, for example) it may improve the participation in outreach activities within the research community. In addition to bibliographies for drug/biologic approval applications it may be helpful to have a short historical description of the development of the therapies, and for these links in particular to be published electronically and accessible to a lay audience.
Better basic education within schools on what research is and how it can be evaluated. Much promotion of research by even highly regarded scientists does not serve the knowledge/long term interests of the public that well. A better approach that helps the public understand and ask difficult questions would reduce the overhyping that we currently have, which is an understandable collusion of a variety of folk depending on the exact work but including media, politicians, sometimes pharma, the scientists and their organisations.
Better science education in schools ...
Increase media coverage and accuracy of portrayal
Web based collaborative public exploration by scientists of how specific research might benefit society and what the big picture priorities should be
Generally, I think these discussions/policies need to start earlier in education for example as fundamental part of undergraduate research projects etc... Also I think the significant segregation between biomedical PHD and MD education contributes to these issues. I believe that MDs have key role to play in supporting/communicating basic research.
no
I believe history shows that the translational progress from important (rather than trivial) basic research results to clinical applications is too slow to be measured at the level of individual scientists.
The requirement to describe the impact of submitted grant proposals on human health is more detrimental than good. This is because basic scientists as well as the institutes they work at end up having to make outragous claims about the impact of their work. As a result the public hears on a near daily bases how cancer has been cured yet they see now benefit in the clinic. This has led to the disillusionment of the public with biological/biomedical research. Without the support of the public, funding for research, whether applied or basic, will not continue to grow and will negatively impact society. Grant funding agencies need to recognize the importance of both applied and basic research and the short and long term benefits that come from both. In addition there needs to be a concerted effort communicate this to the public by giving real life examples of how basic research has benefitted their life. In addition, grant funding agencies need to have a little more risk tolerance. It is through risky projects that we have the potential to make a huge advancements in our understanding and in the development of novel therapeutics. In the current state of funding only small incremental steps are taken, which in the end slows down progress. In order for this to happen this needs to be communicated to the public so that they are supportive and understand that their may be failures but it is a the potential of making huge gains.
Improve gender balance of scientists and improve ability to combine family life with scientific life. Family and friends is the best way to communicate scientific importance and women tend to be the ones that maintain family life irrespective of whether they are also scientists.
- Stop expecting basic research to provide IMMEDIATE societal benefit. In the long run, excellent basic research will still do this. - Lobby and educate scientific administrators about the ongoing need for basic research, to underpin societal benefits - Lobby and educate the political class in this too - Lobby and educate the general public, who are in general well disposed to high quality research
no
I think the benefit potential of basic research is heavily dependent on the attitudes and talent of individual scientists. In my experience some basic research has led to applied outcomes i.e. patents, companies, health benefits, whereas some 'applied' research is clearly not going to result in any tangible outcomes, simply because the researcher is unable or unwilling to translate that research into an outcome. If I had to offer one opinion it is not to fund research which is of poor quality or from researchers with a poor record, simply because the health issue they are investigating are of importance or popular. Good 'basic' research conducted by talented individuals should be funded because if any benefit potential is identified, these people are the ones who are likely to take it forward to a 'benefit potential'.
It is not possible before conducting basic science research to know what outcomes it might generate. This is inherent in the experimental nature of basic science. This attribute does not lend itself to a funding system that preceeds the study - ie that judges different projects on what they promise. Rather it lends itself to a 'payment' funding scheme - i.e. that judges based on what is actually delivered. With this in mind, if reseracher were paid 'royalties' for basic work now published that has led to societal benefits (maybe work that answers clearly set out key questions that the funders make publically available, along with a 'price tag' for each) then a market could be created that rewards researchers who steer their basic research towards questions with societal benefits, and conduct it in a manner that is robust and therefore useful (rather than novel and underpowered). These payments can then be used by scientists to 'experiment' into new domains without the need for up-front grant evaluations.
Politicians should be made much more aware of the importance of basic research. There is too much stratificastion into applied fields, which are important but only when balanced by fundamental research. As David Mitchell put it eloquently, 'If academic endeavour had always been vetted in advance for practicality, we wouldn't have the aeroplane or the iPhone, just a better mammoth trap.'
I feel there is already enough appreciation in society of science - biomedical or biological. I think specific funding for basic science shouldnt bother assessing impact in terms of applications, this comes naturally with further investigations. Focus of studies is a good thing and they shouldnt have to over stretch to "fit" into one category or another.
It is very important to greatly increase the profile of basic science and biomedical science in public media, newspapers, TV, radio, magazines,and to emphasize the importance of both scientific education for a scientific workforce and for education of the general public. The advantages of proper public funding of scientific research and outreach programs (internships, summer programs, work-study, trips to and from grade schools and high schools, summer transition programs from Community Colleges into University science education) should be realized by the local, state, and federal government, foundations, and civic organizations.
Involve the funders- that is, the public, always.
In last few decades, the role of basic biological science focused on such fundamental questions as the laws of nature/evolution, the mechanisms of animal/plant development, the processes involved in the initiation and progression of specific diseases, appears to be almost completely eroded and replaced by clinic-oriented studies promising quick cure or even eradication of diseases. For example, in cancer research, it feels like that it is not possible anymore to publish a basic research paper or receive funding if you do not promise to cure cancer if not today, so at least tomorrow… Unfortunately, a lot of profit-oriented companies and “pharma-affiliated” scientists are paving the road to “destine-

to-fail" clinical trials, using desperate cancer patients as test objects in hope of big money and fame. I think that this practice is unethical and should be discouraged, if not stopped. It would eliminate unnecessary trials demonstrating miserable "successes" of the majority of new cancer drugs that were "pushed" into trials. I believe that basic scientists should be allowed to have time to conduct their research and not be forced to submit "tomorrow-cure" grants in hope of survival. If basic science would be supported better, it would help to restore the value of unbiased science and to replace an avalanche of papers with non-reproducible clinic-oriented "findings" published for the sole purpose of grant funding.

Government should stand in for basic science. Highlighting of past achievements

Increase job security for scientists! While we are chiefly motivated by satisfying our curiosity rather than by a fat paycheque, this does not mean we can be treated as badly by society and taken for granted as we are now. Short-term contracts and the fear of becoming unemployed are extremely destructive and nowadays the main barrier to job satisfaction for scientists. We do have families and responsibilities, and as Science is an all-encompassing activity, there is no chance to build a more stable career on the side. Science itself suffers as scientists are forced to pick low-hanging fruit in order to publish quickly.

Focusing on scientists as personalities rather than the project proposals is a disastrous mistake. It rewards political maneuvering. It rewards a cult of personality. It takes our gaze away from what's important which is the value of the ideas that are to be considered. We should recognize that our best scientific minds often come with personalities that are disorganized lazy and unaware of deadlines. We just need to get these people admins. What we're doing right now is encouraging superstars who have great organizational capability, charm and ambition but little native sense of that new direction that is going to really pay off. We would be more successful if we gave sociophobic introverts admins to help them prepare their grants.

Have scientists educate the public on the importance of unconstrained basic science to the future of breakthrough biomedical discoveries.

The questions in this questionaire are extremely poorly phrased.

- It would be valuable to take current scientific topics in the news (mission to Pluto, Higgs Boson, GMO foods, CRISPR/Cas9, deep sequencing) and elucidate the basic research findings and the scientists who did the work that got us to these technologies/discoveries. - Describe with examples the NON-LINEAR, NON-TRANSLATIONAL nature of great scientific discoveries and their eventual applications: cell death pathway in C. elegans and cancer, timing mutants in Drosophila and circadian rhythm, and so on.

I strongly suggest the basic science of any field is important for the development of potential biomedical research for the society. Both basic and biomedical research depends on each other to expand the lifespan of human health.

For some government grants (such as the DoD), scientists are required to write an abstract for the general public that is different from their scientific peer abstract. I find that this is a fantastic exercise for both the scientist who can work on making their research more accessible, and for the layperson who gets a better understanding of what some of their tax dollars are funding. It's very frustrating as a scientist to hear the public mock research about scientists studying worms pooping or some other such over-simplified non-sense when in fact those researchers are making valuable contributions towards understanding cancer or longevity or ??? The layperson abstract is helpful to counteract this misunderstanding of science, and it also helps the scientists realize they need to be able to relate their research to all audiences.

Increase funding for NIH as a % of GDP; don't shut down the government again, causing massive cuts to NIH-funded research programs. Any policy which increases public awareness and comprehension of science is very important and more thought should be committed to policies in this vein. Only when people actually care will the first part of my statement have any hope of being fixed.

The questionnaire is very poorly put together. The questions/statements could be a lot clearer (and therefore result in data that is more reliable). I would suggest validating it in a small sample and ensuring it is reveiwed for readability before sending it to a wider audience.

To promote societal benefit: - More scientists or people with MD or PhD level scientific training in public office (legislature) - Standardize science curriculum in secondary schools to make sure all children have introduction to biology, chemistry, physics and human physiology; emphasize scientific process and benefit of scientific research - More lobbying for science funding. - Bring back Bill Nye, The Science Guy, to public television; and/or promote TV/movie with scientists as protagonists to encourage more children to pursue STEM education and careers To promote scientist satisfaction: - Increase funding for all levels of scientists, but especially for trainees, early and mid-investigators - Decrease time between paper submission and publication (editors should restrict reviewer's ability to ask for more experiments); often takes 1.5-2 years in my subfield to get something published - Decrease time between grant application deadline and when funding starts (typically ~1 year now) or give more flexibility for start date; one of the other problems is not knowing 6 months in advance whether you will have funding for your salary or the salary of people in your lab - More institutions guaranteeing bridge funding between grants to support labs during low funding times - More core facilities for expensive equipment to allow smaller labs to compete with big labs that can afford to buy fancy equipment - Reduction in fringe taken from postdoctoral and early/mid-career investigators to support department overhead, university or hospital. This is one concern about putting more basic science labs in hospitals. If clinical money supports science labs, it works. But it doesn't work to suck off money from research grants to support hospital, hospital administrators, or wider university system. Or different percent fringe based on size of grant. Why should 33% of a postdoc's $50K salary go to the department and 33% of PI's $1 million grant to go department? Significantly decreasing postdoc contribution to admin costs and minorly increasing large project grant contributions would be fairer (and allow postdoc to pay rent, eat and travel home).

Greater support, funding and career development opportunities for young, burgeoning scientists are sorely needed. Too often, younger people are easily dissuaded from pursuing a career in science because of the extreme difficulty in achieving a stable position after graduate school. The scientific community is extremely top heavy and too much emphasis is placed on 'armchair' senior researchers who contribute little to scientific advancement.

Implement basic research in both private and public sectors and/or strengthen the link between basic and translational research.

Every bit of basic research has the potential to contribute to society. It is not possible to calculate, estimate or predict for most of it. If a grant body could predict were the next revolutionary discovery is going to be they would be doing it. Society needs to accept that a certain amount of funding needs to go to research that may not seem to have direct benefit. It should come from policy makers to promote this fact, but at the end they tend to look for short-term profit and short-term reputation boosts.

My apologies, but I was unable to fathom the inclusion of the following point in some of the questions above: "Acknowledge participating scientists during grant assignments, promotion, hiring etc." The point seemed unrelated to policy being considered. My own view is that scientists already (can) spend an enormous amount of time responding to concerns of society. The recent appearance of "compliance officers" in institutions around the UK is another step in the direction of people who are not scientists (and neither understand nor care for the science) being in positions of power, acting as policemen rather than facilitating the scientists to acheive their goals. I would remove most "advisors" and "officers" from these positions (even if they have a medical, veterinary or science degree), and allow local committees of working scientists to determine their courses of action, rather than administrators (who are often people with meagre and inappropriate education, interested mostly in growing their jobs). On another subject, it is most interesting to note that the MRC has historically let scientists get on with curiosity-driven research and who have (as a result, I believe) made many noteworthy discoveries (leading to Nobel prizes and the like), whereas the Wellcome Trust has insisted on biomedical relevance and now "translational research" run mostly by clinically-trained scientists, who (as a result, I believe) never received a single Noble prize, since much of the work only follows on from the real discoveries.

I found this questionnaire problematic in that it seems based on the assumption that there is a clean distinction between basic and applied research, and that theses two research modes are somehow in competition or conflict. There is by now ample evidence that research with no specific applied goal can provide transformative benefits to society. It is also clear that someone has to do the work to make that happen. Sometimes its the same person. Sometimes its a different person. It does not matter, as long as it happens. I think generally valuing the diverse and wide range of contributions of different scientists across the research system, and far beyond papers and patents is important. This is hinted at in some of the policy suggestions above. However, there are no rules for this stuff. This is perhaps the main point. As the system has become more competitive, we have tried to invent quantitative rules for assessment in the entirely misguided hope that they would provide objectivity. While these measures may be entirely objective, they don't actually measure the characteristics of interest. Instead they drive perverse incentives to score highly against an arbitrary set of metrics. This is not research, which is a creative process characterised by everything from inspiration through serendipity to dogged determination. Judging which science to fund has to rely on qualitative judgements of peers, giving appropriate balance to a range of factors that are not equally relevant and important to all projects. Trying to ensure the maximum benefit is derived from the results requires everyone to be open minded and engaged with diverse audiences. Here again, can be inspiration, serendipity and/or dogged determination that gets the job done. The best policies for research involve creating an environment in which these characteristics can thrive. This requires maintenance of high levels of diversity and flexibility in the system, not restrictive rules.

Establish an intermediary body which acts to assign the grants to the research. This body will collect and categorize the incentives of potential benefits of research, and assign proportions of grants to various fields of basic research that they deem fit. The researchers in the departments will apply for these grants according to their field of research. This effectively creates a market for research grants, where the incentive would be directed more towards an equilibrium between the social benefit and the research interest. The balance between these two will dictate the amount of money allocated to the research field.

There is huge knowledge gap between basic science and medical or clinical research although we start our education with same knowledge on biology, chemistry etc. To tackle this problem, syllabus for basic science or medicine should have some overlapping subjects. Physiacians should be encouraged to participate in reasearch and vice versa in more numbers. Such people possibly can communicate for easily or clearly about the social benifit of potential basic research. Thanking you

The concept and importance of basic research should be addressed better at school (elementary, middle, high).

involve scientists in decision-making processes encourage participation to regional and national/international committee provide training and resources for public involvement involve researches and universities in high school programmes design personal or lab-dedicated incentives for public engagement

It should be recognized that overfunding large and established labs at the expense of a vast number of smaller but reasonably productive labs will create enclaves of excellence, while at the same time drying out the reservoir of new research personnel, ideas, talents and discoveries. And will inevitably generate a self-serving body of scientists busy awarding grants to themselves and their associates.

This survey is totally biased by the artificial division between biological or basic reserach and biomedical research. There is no formal definition of this two areas, nor any agreement on their definition in the community. Thus you leave totally to the reader to apply her/his own definition or perception on what is biological or biomedical research. This is a major methodologically minus, and I think also that it is outdated concepts. I think there is good and bad science and we should have policies to select good science regardless how far or close it is to provide a therapy. Historical evidence shows that major health breakthroughs come from good and unbiased research independently from the declared goal of the original investigation. I think you're dealing with a false problem.

the government should assign prizes to individual scientists and/or to research groups and/or to entire institutions based on their contribution to advertised and well recognized goals of public government. companies or bodies representing companies should assign prizes to individual scientists and/or to research groups and/or to entire institutions based on their contribution to advertised and well recognized goals of companies or bodies representing companies scientifi and leraned societiesshould assign prizes to individual scientists and/or to research groups and/or to entire institutions based on their contribution to science This to some extent happens in the US and some Europena countries, notably the UK, but NOT in most other countries. Italy, from this point of view, is a total disaster.

Thesis that scientific results should be obtained by group (a collective) of scientists is not absolutely true. It is necessary to give the possibility to carry out an individual (personal) research, of course this research should be estimated in advance by reviewers with high scientific potential.

The key to success of science and its application to practical matters that benefit society and the environment is that basic research should be the number one priority without any direct need to demonstrate its potential links to human health or any other practical consideration. The main reason for this assertion is that how basic concepts will be applied in the future is by nature unpredictable. What is predictable is that scientific progress on the basic front will always lead to some new innovations that do benefit humans and the earth more broadly. Support basic research and the rest will take care of itself.

The policy statements above are fraught with problems and I am not in favor of any of those policies. The only worthwhile goal of basic research is Discovery, in my opinion. These discoveries are not always earth shaking, but, a true discovery is always appreciated and admired by basic researchers. It is not possible for the public in general to appreciate basic research because this is not a profession, but a way of life. It is an obsession that inflicts a few people. I have always told my students that basic research is NOT for everyone, and there is nothing wrong with them if they do not like the process or the way of life. Trying to explain our "neurosis" to the public is not likely to change the public's mind about basic research. There are other types of research, particularly those that involve human subjects, that the public can relate to. Unfortunately, those types of research do not make discoveries, they are "studies", not "research". The policy stated in this survey can improve the relationship between scientists and the public on those "studies", but, they will not likely to have any impact on the practice or the public support for basic research. I have personally done a 10-year experiment with my career by moving my basic research lab into a cancer hospital. The result of that experiment is, unfortunately, negative. My objection to the polices stated here is based on data, not opinion. I wasted 10 years of my professional life trying to explain my basic research to clinicians and the public. With the budget shortfall that most Western countries are facing, there will be less and less support for basic research. However, I believe that basic research will still be done by those who simply must find a way to live with their desire to make discoveries.

The emphasis on basic science should be at research universities. University professors should be given resources to address interesting questions regardless of their practical applications. Medical research centers and specialized research institutions should then select and follow up. In 1976 one of my applications was funded by the NSF on "Respiration-deficient Chinese hamster cells in culture". One of Senator Proxmire's underlings called me to ask why I was interested in respiration-deficient Chinese (I guess I was a candidate for a "Golden Fleeze of the Month Award". This was ten years before the first publication on human patients with mitochondrial disease, and our research was purely curiosity driven. Our cells became model systems. I have written a well received book on mitochondria and recently an invited review for the American Journal of Inherited Metabolic Disease. I have also been invited to China for six years in a row to teach graduate students about the molecular basis of mitochondrial diseases. My guess is that nowadays our application would have been instantly triaged

Scientists will write anything if they think that they can get money from it. Punish those who have been obivously lying to get the grant. Right now there is no repercussion for PIs exaggerating in their applications. Instead of looking at the impact factor of a journal look at actually useful metrics, such as citations over time etc. I want to kill myself when I see who get's funded for what. Fucking idiots.

Involvement of active scientists in the training of media-relations personnel (i.e., journalists), of both trainees in the discipline (e.g., classroom settings of journalism schools) and of active journalist (e.g., through seminars or workshops).

Reddit science AMAs are often on the front page so clearly there is an interest from the public to engage scientists. Maybe more online interactions like this where people feel safe to ask "stupid" questions.

I think that public scientific literacy is the keystone of this issue. It would be great if scientists communicated more effectively with the public, but that requires a receptive audience. I would think the most important factor in this issue is science and math education in grade school. This is probably also affected by the way that the media misrepresents scientific findings, although that might decrease or have less effect if future generations are more aware of the scientific process.

Make every politician and journalist covering any science participate every 6 month in a 2 hour meet-and-greet session with basic and bio-medical scientist.

No

I think maximal outreach to the public (both in the general community and crucially to lawmakers) about the benefits of basic research is where the emphasis should be.

Promote a Scientific Journal devoted to publish paper on health benefits of basic research

Improve K-12 science education. An unprepared mind will be unprepared for the proposed policies.

Get kids into science. That's the only way to change the mindset of the generations.

None

Substantially increase funding and funding accessibility for research

Current research is driven by end-result oriented, rather than innovation driven. More and more grants are inclined to fund who can deliver result irrespective of either the method or level of scientific imagination. Let me give an example. Let's imagine, a valuable iron ball (magnetic substance) fell into a river (known within few sq. miles area with moderate depth. Say, there is a call for application for project to recover the "iron ball" from the river. Say, 2 independent PI submitted grants on this. PI-1: Proposing to scan the whole water of the river (like genome-wide search) with net and cite the certainty of capability by using similar method to recover similar thing from similar situation. PI-2: Thought to use scientific knowdge knowing the special property of the ball and proposed to use powerful magnet to scan and recover the ball, but could not cite their prior successful attempt using this method. NIH would prefer to select the PI-1's project. But to my mind PI-2's proposal is much more scientifically driven, more satisfying and has certainty of success. Practicability of any scientific discoveries should be investigated by the clinicians (in case of biological/biomedical) or technologists/engineers for other technology products) consulting with the discoverers. Most of Credit should go to the scientist who discovered it, rather than people who got those to public use. We all know that we are successful in sending satellites to space using Newton's law of gravitation. But Newton's motivation was not due to the possibility of future use. I strongly feel that we need to enhance our power of intuition/imagination rather than the statistics generated target validation science to exercise excellence in scientific research. Another thing, use of scientific knowledge/imagination is restricted by the successful grant wirting criteria set by funding agencies. If Albert Einstein would have to do research with funding, it would have been impossible for his grant funded for his proposal on findings at patent office!!

I think that biomedical research must be mostly publicized, because more patients don't understand the real role of the bioscience for the general health. Some reason of this can be the bad publicity for animal sperimentation, for drugs in general and natural cures (that there aren't miraculous cure of cancer). This could be a real motivation to encourage young to follow this course.

One of the major problems basic science (and science in general) faces, is misrepresentation by the media and by conservative politicians. The main factor is false equivalence giving equal weight to dubious if not downright false opposing viewpoints. This issue is difficult to counter on the scale of public seminars. Seminar attendees are likely to be highly selective population that is probably already holds favorable views towards science. Scientific societies should work to educate the mainstream media to stop viewing corporate representatives or (for lack of a better term) fringe scientists as qualified scientists without significant conflicts of interest or bias. Similarly, politicians should be held more accountable. When a politician claims "I'm not a scientist..." and then espouses an anti-science viewpoint, reporters and scientific society should swiftly respond and question why these individuals would belittle or deny the validity and contributions of science to their personal well being (i.e. disease treatments, weather predictions,

computers, communications, agriculture, etc.). While it's great to have Carl Sagan or Neil deGrasse Tyson as advocates, they have limited reach and need platforms to reach much broader audiences more frequently. Scientific societies need PR and even something like political operatives to expose industry and anti-science groups for their agendas and questionable credentials. Instead of not engaging or expecting some sort of even playing field, scientific associations need to get media savy and do more to reclaim what science stands for and represents.

I think there should be basic mentoring/management/accounting/marketing training for young and older scientists. Basic business skills to improve lab-lab communications, lab budgeting, lab-clinician communications, etc.

Sometimes in the public eye basic research is not understood, and some people (even fellow scientists) may hear about a particular project and not understand its implications and be thinking "What is the point of this research?" and I think this question really needs to be addressed and in a simple manner where everyone can understand why they are doing this research and why it will be a benefit to us.

An open policy is always important. Providing the research results that led to this drug and/or clearer understanding allows society to see their tax dollars at work. Though any public funding that led to a company to capitalize should be reimbursed to the public fund, e.g. NIH. There is always a bias in grants and manuscripts to the actual person rather than the "idea". Favorably or unfavorably. Manuscripts and grants should be reviewed without the name of the authors to the reviewers. There should be a process in place so that everyone reviewing manuscripts and grants know that they are from an acceptable institution/organization and have passed the preliminaries. This will allow the ideas and data to stand on their own. That is all I have for now

The only comment I would like to include here is that it is very ineffective to tell basic scientist what to research(RFAs). I don't think it is not possible to predict in which area of research the next breakthrough will happen.

It would be nice if the next NIH Director had a better understanding of the necessity for NIH to support basic research to advance societal goals It would also be an improvement if there was an NIH requirement to retain and leverage public-funded intellectual property to ensure affordable drug/treatment availability to everyone.

Scientist should participate in networking more often

Obviously. there needs to be a multi-tiered system. Some junior scientist are project oriented and may not be fully aware of the potentials of their work. Senior scientist on the other hand can have more latitude to pursue avenues that while having potential clinical significance, are high risk and may not be able to attract funding. A senior scientist should be evaluated on their accomplishments and impact in the field rather than their perceived importance by other colleagues. Many good ideas are not done because of a lack of funding. Many attractive proposal are awarded because of the research tools used rather than the merits of the actual work. It is a tremendous waste to have established scientist cut off from funding at the time that they can best focus on the big unsolved problems.

I think the media could really help by reporting the origins of progress in terms of the basic science and scientists that initaited and brought to fruition a finding, even if it is not world-shattering progress. The public needs to be educated about the scientific method and how failed hypotheses lead to new insights that can change the world. For example, the discovery of miRNA --- how it occurred and where it has led would help people understand the value of basic research. I am a glycobiologist and have determined many functions for sugars attachd to glycoproteins. Even most scientists do not appreciate the importance of sugars on glycoproteins. Understanding how they are useful in recombinant therapeutics that treat disease would be helpful to the general public as it would be a lesson in how to think scientifically.

It is obvious that any discovery is built upon the work of others..the major limitation that basic scientists face in recognition for this is that there is no clear way to credit people for their discovery. Citations are nonsense as you need to be cited (in fact, many papers are published in the tope tier primary literature that fail to cite second or third tier work that is important for that discovery as well. There is really no way to fix this because you don't know why a paper isnot cited--it may be that the authors of the current paper did not know it, did not wish to acknowledge it, or find it to be incorrect and not well supported by the data.

educate scientists in public communication skills. Choose highly effective scientific communicators to engage with the public and media.

I think discussions between scientists and general public and/or patients will greatly benefit the advancement of science. Such discussions may stimulate motivation to the scientist and create awareness and understanding about science (especially basic science) among public.

Need more policy makers who are scientifically literature, with some experience in basic science not simply owning medical degrees.

involve public participation, especially school children.

We do need to reach out to the public more about basic science and inform them about how our research is funded and how inadequate it is.

I work at a medical institution, so my opinions about health impact of even basic research are probably a little biased.

involve scientists in communication with the public.

The tone of these questions seems to imply that basic biologists need to justify the worthiness of their work. Yet, if only translational scientists in hospitals existed, they would rapidly run out of biological knowledge to "translate" to the clinic. There needs to be a better understanding of how truly lacking our understanding of biology actually is, so that people will understand that new discoveries mostly come from people who seek a mechanistic understanding of cellular function, not an understanding of disease without an understanding of normal function.

1- Societal education (increase people awareness of the contributions of basic research to our daily lives) 2- Change the composition of policy boards and committees 3- There is a clear disconnect between funding policies and recognition. For example, while most Nobel prizes in science and related disciplines praise basic research findings, grant funding is strongly biased toward translational focus. This should be changed.

obvious factors affecting basic scientist satisfaction and research are the tightened availability of funding, and governmental scrutiny over how these funds are applied. Politicians require that federal (and state) funds be justified by awarding projects that' benefit human health'. Better education of these leaders, in addition to educating 'the public', as to the way scientific discoveries are made, and the essential role of basic research in providing the building blocks for applied research, would go a long way towards revitalizing the morale and vitality of the research community.

The basic/applied distinction has always been highly problematic and isn't helping the debate much. In answering this survey, I suspect that people have very different ideas about what it's taken to mean... To get into more productive concepts, perhaps Stokes's "Pasteur's quadrant" (1997) is a good starting point.

There needs to a better division of basic and biomedically-applicable research here in Europe. While the NIH and NSF provide clealy delineated divisions of granting, in Europe this distinction is less clearly drawn. Liekly this has become exacerbated in the recent times of waning funds available to research As such, frequently basic researchers are forced to extrapolate a medical connection to their applications. Similarly, far too much money destined for biomedical research is diverted to purely basic research which can have only a serendipitous impact upon the diseases they pretend to study. I would suggest a bolstered basic research organization, and simultaneously the strict implemenatation of short-term medical applicability terms to non-basic research funding. Realistically, even in the USA, the NSF is grossly underfunded. In this way, far too much unimaginative, hype-driven science gets performed because scientists no longer feel the freedom or capability to take risks. Therefore, everybody sequences everything and generates gene list upon gene list with little real benefit to a patient, instead of trying to develop new drugs/compounds. Another essential policy touched upon in this survey is the need to cap funds for individual groups. Gone are the days where one oligarchical lab head could oversee 50 postdocs and publish 5 Science papers a year. The research group leader community is aging, and more money needs to be divvied out to younger researchers. I see far too often big names, established, historical groups, bedrocks of their fields, issuing papers which are retracted, contain clearly untrustworthy data or unrepeatable data. Let's start slimming down the behemoths, by forcing them to produce with lower net funds, giving the difference to new researchers getting a start in academia or trying to make the jump from industry.

We should often remind people that knowledge is always power

1. Scientists are too low paid and the brightest most driven people are discouraged from doing it for this reason. 2. In the US, biomedical research institutions do not provide enough solid support (eg hard salary) for scientists, making for deep dissatisfaction and low moral in a competitive funding environment. 3. We are training too many young scientists in US graduate and postgraduate positions given the limited jobs - we should train more scientists in other area that could work in the future such as law & business.

Good survey

I don't think any of these methods gets at what I think is an important goal: to bring basic scientists together to think about health related problems. Simply having basic scientists at hospitals doesn't help if they are not actively involved with clinical problems. I believe more chances for physicians to talk with basic scientists has a potential for a discussion of problems from two very different perspectives. For real translational research, one needs communication between these groups or, at the very least, a way that basic scientists can communicate their knowledge to clinicians.

the last few questions were annoying. Each question had 2 components. Eg (1) Promote more seminars and academic discussion concerning the purpose of scientific research and the role of scientists in the society. and (2) Acknowledge participating scientists during grant assignments, promotion, hiring etc. i agreed with 1 but not 2. The second

component made it sound like a threat or a cudgel.
Most scientists know what to do without undue interference from ethicists,philosophers, lawyers, well-meaning people, government commitees, and the general public. Practical benefits are obvious, and in all cases will flow naturally out of the basic research. To facilitate that flow would be a most optimal use of governent manpower. Let me underline your gratest truth: money should go to scientists, not projects. The countries that follow that truth harvest the best science ideas.
Abolish reporting. Thery are a terriblewwaste of time. For thehonest scientist, following the publication would provide the funsing agency all the reporting it needs. For the dishonest scientist, reporting are unblieveable anyway.
Perhaps institutions and professional societies could do more societal outreach. I like ideas that make it easier to do good research (lab location definitely influences conversations & thought processes!), but am leery of anything that says "scientists need to add to their already overburdened schedules to better communicate with the general public".
-higher/decent salaries -more focus on good/solid science than publications in high impact factor journal during research quality assessment -separation of scientifically/public health interest from commercial interest
Showcasing how basic science has paved the way for translational science is key- And this should start at all levels of science/biology education.
Promote dissemination policies and visible events, possibly through collaboration among international agencies (e.g. EMBO, WHO, HFSP, NIH, ...).
**This table has more than 100 rows. Click here to view all responses**

Statistic	Value
Total Responses	153

25. Timing

#	Answer	Average Value	Standard Deviation
1	First Click	94.62	1,250.30
2	Last Click	723.38	7,093.39
3	#QuestionText, TimingPageSubmit#	746.49	7,093.31
4	#QuestionText, TimingClickCount#	27.62	10.74
