# Supplementary material for: Increasing both the public health potential of basic research and the scientist satisfaction. An international survey of bio-scientists
Source: F1000Res. 2016 Jun 1;5:56. Originally published 2016 Jan 12. [Version 2] doi: 10.12688/f1000research.7683.2 (PMC4909114; doi:10.12688/f1000research.7683.2)
Supplement: Questions and responses of the survey — “QUESTIONNAIRE.pdf” contains the questions of the questionnaire. “QUESTIONNAIRE DATA.csv” contains the responses of the questionnaire. “FINAL REPORT.pdf” contains the summary of the responses of the questionnaire. [file f1000research-5-9472-s0000.tgz › e2cce3ea-006d-4143-befe-10b5dd86c3e2_Data.zip!/Data/QUESTIONNAIRE.pdf]

FIRC INSTITUTE OF MOLECULAR ONCOLOGY

This questionnaire is for a research study conducted in partnership between IFOM (FIRC Institute of Molecular Oncology-Milan, Italy) and HSPH (Harvard T.H. Chan School of Public Health-Boston, USA). It is being sent to scientists working in biological/biomedical research in different geographical areas. Participation is completely voluntary and anonymous.
The questionnaire contains 20 questions divided in two pages. You can also choose to skip questions.
Please do not share the questionnaire web link with anybody.

If you have any questions about this survey you can contact Dr. Andrea Ballabeni (aballab@hsph.harvard.edu) or Dr. Carmen Sorrentino (carmen.sorrentino@ifom.eu)

Your input is very important to us.
Thank you very much for your time.

Q1 About you...

Please indicate which of the following best describes you:

- ○ Principal investigator
- ○ Post-doc
- ○ PhD student
- ○ Other (please specify)

Your gender:

○ Female

○ Male

Your age:

	20	30	40	50	60	70	80	90	100
Years:									

Q2 In which of the following geographical areas do you work most of the time?

Q3 Approximately, what percentage of your research do you consider to be basic?

	0	10	20	30	40	50	60	70	80	90	100
%											

Q4 Grants should not always be assigned in an 'all-or-none' fashion. Instead there should be the possibility to partially fund scientific projects/scientists so that more projects/scientists are funded even if this would decrease the numbers of projects/scientists fully funded.

○ Complete disagreement

○ Some disagreement

○ Some agreement

○ Complete agreement

Q5 There should be a cap to how much a given scientist/laboratory can be

funded, in order to attenuate the bias in favor of established scientists/laboratories.

- Complete disagreement
- Some disagreement
- Some agreement
- Complete agreement

Q6 In the future, an increasing share of funding should be granted to the scientists rather than to the projects.

- Complete disagreement
- Some disagreement
- Some agreement
- Complete agreement

Q7 Basic scientists can ponder about the future indirect practical benefits of their research without losing their "basic status".

- Complete disagreement
- Some disagreement
- Some agreement
- Complete agreement

Q8 Your personal motivations as a scientist are from:

	Not a motivation	Minimally important	Moderately important	Important	Very important
Pure advancement of knowledge, regardless of future applicability	○	○	○	○	○
Health benefit to society (not necessarily in the near future)	○	○	○	○	○
Gain of prestige	○	○	○	○	○
Gain of money (for personal purposes)	○	○	○	○	○
Satisfaction of your curiosity	○	○	○	○	○
Satisfaction from solving puzzling problems	○	○	○	○	○

Q9 What should the most important goal of publicly funded basic BIOLOGICAL (not biomedical) research be?

○ Health benefit to society (not necessarily in the near future)

○ Pure advancement of knowledge, regardless of future applicability

○ Other (please specify):

Q10 What should the most important goal of publicly funded basic BIOMEDICAL research be?

○ Health benefit to society (not necessarily in the near future)

○ Pure advancement of knowledge, regardless of future applicability

○ Other (please specify):

Q11 Although it is difficult to assess the potential future health benefits to

society from basic biological/biomedical research as described in written proposals, some degree of estimation is always possible.

- Complete disagreement
- Some disagreement
- Some agreement
- Complete agreement

Q12 Written proposals about basic biological/biomedical research generally contain a section discussing potential future health benefits. These sections increase the likelihood that a project benefits future public health.

- Complete disagreement
- Some disagreement
- Some agreement
- Complete agreement

Q13 Writing the sections discussing potential future health benefits takes too much time.

- Complete disagreement
- Some disagreement
- Some agreement
- Complete agreement

Q14 The sections discussing potential future health benefits should be eliminated for .... grants.

- ...no...
- ...a few...
- ...most...
- ...all...

Timing

These page timer metrics will not be displayed to the recipient.

First Click: *0 seconds*

Last Click: *0 seconds*

#QuestionText, TimingPageSubmit#: *0 seconds*

#QuestionText, TimingClickCount#: *0 clicks*

End of page 1.
You can now go to page 2 (please note that once you go to page 2 you cannot return to page 1)

>>

A pilot study based on a similar questionnaire submitted to basic scientists of the Harvard Medical School and affiliated institutes (Massachusetts, USA) has been recently published [http://f1000research.com/articles/3-20/v2]

In the next part of the current survey we would like your feedback on six specific policies designed to increase the societal benefit potential of basic research (these policies are discussed in the published pilot study but you do not need to read it to answer the following questions).

Q15 Please evaluate the following policy:
"Locate more basic research laboratories inside or in close proximity of hospitals"

	None	Low	Medium	High
Effectiveness (societal benefit potential)	○	○	○	○
Effectiveness (scientist quality of work and satisfaction)	○	○	○	○
Feasibility	○	○	○	○
Favorability for the policy (i.e. are you in favor of this policy?)	○	○	○	○

Q16 Please evaluate the following policy:
"Organize more educational and discussion meetings between scientists and the general public or patient associations. Acknowledge participating scientists during grant assignments, promotion, hiring etc."

	None	Low	Medium	High
Effectiveness (societal benefit potential)	○	○	○	○
Effectiveness (scientist quality of work and satisfaction)	○	○	○	○
Feasibility	○	○	○	○
Favorability for the policy (i.e. are you in favor of this policy?)	○	○	○	○

Q17 Please evaluate the following policy:
"Promote more seminars and academic discussion concerning the <u>purpose of scientific research and the role of scientists in the society</u>. Acknowledge participating scientists during grant assignments, promotion, hiring etc."

	None	Low	Medium	High
Effectiveness (societal benefit potential)	○	○	○	○
Effectiveness (scientist quality of work and satisfaction)	○	○	○	○
Feasibility	○	○	○	○
Favorability for the policy (i.e. are you in favor of this policy?)	○	○	○	○

Q18 Please evaluate the following policy:
"Promote more seminars and academic discussion about the concept and definition of basic research. Acknowledge participating scientists during grant assignments, promotion, hiring etc."
(For example, should basic research be conceptualized as purely curiosity-driven, or could basic scientists also consider future indirect practical benefits of their research?).

	None	Low	Medium	High
Effectiveness (societal benefit potential)	○	○	○	○
Effectiveness (scientist quality of work and satisfaction)	○	○	○	○
Feasibility	○	○	○	○
Favorability for the policy (i.e. are you in favor of this policy?)	○	○	○	○

Q19 Please evaluate the following policy:
"Have ethics consultation services for scientists inside research institutes, with easily accessible information about these services"

	None	Low	Medium	High
Effectiveness (societal benefit potential)	○	○	○	○
Effectiveness (scientist quality of work and satisfaction)	○	○	○	○
Feasibility	○	○	○	○
Favorability for the policy (i.e. are you in favor of this policy?)	○	○	○	○

Q20 Please evaluate the following policy:
“Provide recognition to basic scientists who have contributed to acquiring key knowledge that leads to tangible health benefits by requiring a "basic bibliography" of seminal basic research articles for each new drug or other biological application”
[e.g. http://www.the-scientist.com/?articles.view/articleNo/38689/title/Recognizing-Basic-Science-Contributions/]

	None	Low	Medium	High
Effectiveness (societal benefit potential)	○	○	○	○
Effectiveness (scientist quality of work and satisfaction)	○	○	○	○
Feasibility	○	○	○	○
Favorability for the policy (i.e. are you in favor of this policy?)	○	○	○	○

COMMENTS
Do you have ideas about other policies that can increase both the societal benefit potential of basic research and the scientist satisfaction, without affecting the fundamental nature of basic investigation? You can use this space to tell us about them or for any additional considerations related to the themes of this survey.

Timing

These page timer metrics will not be displayed to the recipient.

First Click: *0 seconds*

Last Click: *0 seconds*

#QuestionText, TimingPageSubmit#: *0 seconds*

#QuestionText, TimingClickCount#: *0 clicks*

Thank you for completing this survey and helping our research!

Just click the submission button

>>
