# Supplementary material for: Increasing both the public health potential of basic research and the scientist satisfaction. An international survey of bio-scientists
Source: F1000Res. 2016 Jun 1;5:56. Originally published 2016 Jan 12. [Version 2] doi: 10.12688/f1000research.7683.2 (PMC4909114; doi:10.12688/f1000research.7683.2)
Supplement: Peer review history

# Peer review: Referee response for version 2

**DOI:** 10.5256/f1000research.9472.r14104

**Published:** 2016-06-02

**Authors:** Nathan Vanderford

**Affiliations:** 1 Markey Cancer Center, Department of Toxicology and Cancer Biology, College of Medicine, University of Kentucky, Lexington, KY, USA

Sorrentino *et al.* have been highly responsive to my previous review and the article is much improved. I find the article acceptable at this stage. With that said, if the authors wish to further improve the article, I believe that it would be helpful to incorporate the details of the statistical analysis within the methods section and it would be helpful to include a table(s) summarizing all the results of the statistical analysis.

 I appreciate the authors’ responsiveness and I have enjoyed reviewing their work.

I have read this submission. I believe that I have an appropriate level of expertise to confirm that it is of an acceptable scientific standard.

# Peer review: Referee response for version 1

**DOI:** 10.5256/f1000research.8273.r13079

**Published:** 2016-04-05

**Authors:** Viviane Callier

**Affiliations:** 1 Developmental Physiology, Ronin Institute, Montclair, NJ, USA

This research paper reports the results of a survey of biomedical and biological scientists about their motivations and attitudes with respect to basic research. They found that basic scientists are mainly driven by a goal to satisfy their curiosity and to advance fundamental knowledge. They also have an aspiration to impact human health, usually in an abstract or yet-undefined way. Prestige is a motivator for some.

The thoroughly reported survey results showed that researchers feel ambivalent about the "statement of public health relevance" sections that accompany most grant proposals to biomedical funding agencies. Some favor getting rid of this section. This is important information for the scientific community and for funding agencies to consider.

This research paper is timely because the importance of basic research has been on the mind of the leaders at NIH, the largest biomedical funding agency in the world, as shown in their recent letter to *Science*. In it, the NIH leadership says that the research community erroneously believes that the "statement of public health relevance" means that the agency does not value basic research, when in fact, the NIH leadership says it does. However, at several agencies including NINDS and NCI, the proportion of basic science proposals received is going down, while the proportion of "hybrid" or applied/clinical research proposals is going up. This means that many investigators are shifting their application strategy towards applied research because they think it is more likely to gain funding.

Ultimately, it is the scientists on review panels, not just NIH leadership, who determine which research gets funded. So, if the agency and the research community values basic research, there needs to be a change in culture so that scientifically sound and meritorious proposals gain funding even if they do not have specific clinical applications. Reviewers in basic science study sections should be instructed to evaluate proposals on experimental design and novelty but not clinical relevance.

Future research on how the tight funding climate has impacted attitudes towards basic research, and ideas on how to reverse the trend of decreasing numbers of basic research proposals, would be very interesting to funding agencies and policy makers.

This is an interesting and well written paper. I have a few suggestions to improve the paper. There are many figures and the information contained in these figures could be consolidated into fewer figures to make it easier to interpret. Statistical analysis of the data would also make the conclusions stronger. All in all, the findings make intuitive sense and it is nice to have the data to confirm these intuitions.

I have read this submission. I believe that I have an appropriate level of expertise to confirm that it is of an acceptable scientific standard.

# Response

**Published:** 2016-05-13

**Authors:** Andrea Ballabeni

**Affiliations:** Harvard School of Public Health, USA

We would like to thank Dr. Viviane Callier for taking the time to read and review our manuscript. We have appreciated reading her thoughts on these topics.

# Peer review: Referee response for version 1

**DOI:** 10.5256/f1000research.8273.r13139

**Published:** 2016-04-01

**Authors:** Nathan Vanderford

**Affiliations:** 1 Markey Cancer Center, Department of Toxicology and Cancer Biology, College of Medicine, University of Kentucky, Lexington, KY, USA

Scita *et al*. present survey research findings on basic scientists’ perceptions of the public health relevance of their work as well as their attitudes toward their own personal satisfaction. This work is interesting especially as it relates to scientists’ perceptions of their work versus the public’s consumption of that work and the associated policies around that interaction.

There are several issues with the current version of the article that lead me to approving it with reservations. 

1. The current title could be improved and clarified. The part on scientists’ satisfaction seems to be an afterthought as written.
2. The specific research questions and/or hypotheses for the study should be clearly stated.
3. Clarification is needed to allow readers to better understand the differences and similarities between “study 1” versus “study 2.” What is the overlap of the study design and findings? A better explanation as to how “study 1” informed “study 2” would be helpful.
4. The data on the respondent demographics and response rates should be clarified (the current figures and text are not clear). Adding this information into the main text, in table format, may be useful.
5. The clarity of the article would benefit from simplifying the presentation of the data: 16 figures in the main text is overwhelming. Can some of the data be summarized and collapsed into a couple of tables and/or can some of the data be included as supplemental material?
6. The findings would be greatly enhanced by including some statistical testing. Related to that, what is the primary outcome variable(s) of the study? What are the independent and dependent variables? Testing for association of the outcome(s) with items such as career stage (eg, PI, postdoc, etc.), gender, age, and geographic location would be very interesting and informative.
7. At the beginning of the soft policies section on page 12 of the PDF, the statement “[w]e tested scientists’ opinions…”  should be demonstrated through statistical analysis.
8. Without statistical analysis, the conclusions should not be written to suggest negative or positive correlations.
9. The results of this study should be discussed in context of the related literature in the “conclusions and discussion” section. Perhaps it would be best to have a separate conclusion and discussion section.
10. Why is it that the text in some of the figure legends is underlined and in other figure legends the text is not underlined?

I look forward to reviewing a revised version of the article.

I have read this submission. I believe that I have an appropriate level of expertise to confirm that it is of an acceptable scientific standard, however I have significant reservations, as outlined above.

# Response

**Published:** 2016-05-13

**Authors:** Andrea Ballabeni

**Affiliations:** Harvard School of Public Health, USA

We would like to thank Dr. Nathan Vanderford for reviewing our manuscript and for providing helpful suggestions and comments. We have revised the paper according to his feedback.

 We summarize here our responses, point by point

1. We have changed the title to better emphasize that we aim at increasing both the public health potential and the work satisfaction.
2. We have now inserted a new sentence in the abstract to clearly define the goals of the study.
3. In the Introduction section we have edited the previous wording and this is the new wording: “We used a slightly modified version of the Study 1 questionnaire. In particular, we added a few questions asking respondents to evaluate current policies used to evaluate/increase the public health potential as well as six new soft policies that we developed after analyzing the results of Study 1.” In the Discussion section we have changed the text and this is the new wording: “The current study substantially confirmed what we had discovered in Study 1. In particular, the new study confirmed what we had previously observed with regard to basic scientists’ motivations and conceptualization of basic research. Moreover, it further reinforces the notion that, while some estimate of the public health potential of basic investigations is always possible, basic scientists believe that the requirement of discussing this potential in research proposals is not effective”. Moreover, in the Introduction section we have also added the following revised text: “Study 1 showed that increasing people’s health and personal prestige are some of the strongest motivations for basic scientists. Moreover, it showed that basic scientists strongly support the idea of non-mandatory policies based on soft incentives to increase public health potential and work satisfaction. Based on these and other findings of Study 1, we designed this second study (Study 2)…”.
4. We would prefer to have the figures about the invitations (Figures S1-S6) and about the response rates (Figures S7-S11) in the “Supplementary material” section because not the main goals of our research. The manuscript appear already dense in information and we would like to include in the main text only the most relevant  set of information related to the analysis of the responses. In addition, the “Overview of the sample” paragraph in the “Results” section already provides a summary of the sample and response rates. However, if the reviewer thinks that some additional information should necessarily be placed in the main text we would be happy to consider it.
5. We agree with the reviewer that 16 figures are more than the conventional research articles contain and this is also the reason why we would avoid adding more figures or tables (e.g. for sample description and response rates) in the main text. The flexible format offered by F1000Research actually encourages the inclusion of figures and graphs. Based on these guidelines, we chose to show the data and convey the messages more through the figures than through the text. Indeed, even if the figures (which are not particularly dense in information and relatively easy-to-read bar/column charts) are more than in the standard average research article, the length of the text is probably shorter than in the average published manuscript.
6. We have now performed statistical analysis on the association of the motivations (Figure 1) with the role of the scientists (PIs, post-docs, students). We have introduced the following new text: “For the motivations we also performed analyses based on the roles of the scientists. We observed that some differences were statistically significant. In particular, PIs are more motivated from “Pure advancement of knowledge” (p = 0.0042) and less motivated from “Gain of money” (p<0.0001) in comparison to post-docs. Moreover, PIs are more motivated from “Pure advancement of knowledge” (p<0.0001) and “Satisfaction of curiosity”(p = 0.0068) and less motivaed from “Gain of money” (p = 0.0142) in comparison to students.”
7. We are not sure about the problem but, after careful consideration we think that the use of the term “tested” is appropriate in this context.
8. There was indeed one point in which we suggested a negative correlation without providing statistical analysis. We have now performed the statistical analysis and amended the text as it follow: “For PIs, in terms of degree of involvement in basic research, there were substantial differences in favorability only in relation to policy A, for which there was a small negative correlation (R2 = 0.986) ( Figures 15a–f)”
9. As suggested by the reviewer, we have separated the Conclusions from the Discussion section. Since the length of the manuscript and the emphasis on figures and results, we would prefer not add more text. However, we have added a note in the Discussion section to inform that an in-depth analysis of the relevant literature as well as a discussion on the conceptual framework are present inside the manuscript describing the results of the first study recently published (i.e. Study 1) (ref. 20).
10. In some of the figure legends the text is underlined because we wanted to emphasize the specific policies. However, we agree that in this way the text is not easily readable. Therefore, we have accordingly modified the figure legends of Figures 13-16.

We hope we have addressed all the reviewer’s comments and suggestions.

 We would like to thank Dr. Nathan Vanderford one more time for taking the time to review the manuscript and provide his useful feedback and we look forward to hearing from Dr. Vanderford.
